# Biogeography influences plant–microbe interactions and natural soil suppressiveness to black root rot disease of tobacco

**DOI:** 10.1186/s13059-025-03911-0

**Published:** 2025-12-28

**Authors:** Alix Catry, Danis Abrouk, Nicolas Fierling, Ana Isabel Serrano Mendoza, Marjolaine Rey, Pilar Vesga, Clara M. Heiman, Daniel Garrido-Sanz, Marie-Lara Bouffaud, François Buscot, Adriana Giongo, Kornelia Smalla, Gilles Comte, Christoph Keel, Daniel Muller, Yvan Moënne-Loccoz

**Affiliations:** 1 Université Claude Bernard Lyon 1, CNRS, INRAE, VetAgro Sup, UMR5557 Ecologie Microbienne, 43 Bd du 11 Novembre 1918, Villeurbanne, F-69622 France; 2 Department of Fundamental Microbiology, University of Lausanne, Quartier UNIL-Sorge, Lausanne, CH-1015 Switzerland; 3 Department of Soil Ecology, Helmholtz Centre for Environmental Research - UFZ, Theodor-Lieser-Str. 4, Halle/Saale, D-06120 Germany; 4 Institute for Epidemiology and Pathogen Diagnostics, Julius Kühn Institute (JKI) – Federal Research Centre for Cultivated Plants, Messeweg 11-12, Brunswick, D-38104 Germany; 5 Institut Universitaire de France (IUF), Paris, F-75005 France; 6 Present address: Department of Biology, Autonomous University of Madrid, Darwin 2, Madrid, 28049 Spain; 7 Present address: Leibniz Institute of Vegetable and Ornamental Crops (IGZ), Theodor-Echtermeyer-Weg 1, Großbeeren, 14979 Germany; 8 Present Address: Plant Protection Products Unit, National Institute of Agricultural and Food Research and Technology (INIA-CSIC), Ctra. De La Coruña, Km. 7.5, Madrid, 28040 Spain

**Keywords:** Rhizosphere microbiome, Root disease, Black root rot disease, Disease-suppressive soil, Metagenomics, Metabarcoding, Soil metabolome, Plant metabolome, Biogeography

## Abstract

**Background:**

In disease-suppressive soils, the rhizosphere microbiota protects plants from root disease(s). However, the soil microbiome follows distinct spatial patterns, and the biogeographic factors shaping plant–microbe interactions and soil suppressiveness remain poorly understood. Here, we use Swiss and Savoie soils suppressive or conducive to *Thielaviopsis basicola*-mediated black root rot of tobacco, to test the hypothesis that plant–microbe interactions and suppressiveness are influenced by both the geological origin and geographic positioning of soils. Soils are compared based on tobacco health, soil physicochemistry and organic matter profiles, taxonomic and functional microbial diversity, and plant physiological responses.

**Results:**

Soil physicochemistry and metabolomic profiling of soil organic matter show differences based on suppressiveness status, soil geology and geography. The taxonomic (metabarcoding of prokaryotes and fungi) and functional (metagenomics) diversity of the tobacco rhizosphere reveals that the microbiota is influenced by geography and geology which, in turn, affects suppressiveness. Additionally, shoot metabolomics shows that tobacco responses are impacted by soil geography and geology, particularly in Savoie soils regarding two nicotinic derivatives.

**Conclusions:**

Overall, suppressiveness is influenced by both the geological origin and geographic positioning of the soils, with distinct patterns in the two regions. In Swiss soils, suppressiveness is primarily associated with major differences in rhizosphere microbiota composition and functions between suppressive and conducive soils. In contrast, in Savoie soils, suppressiveness is linked to distinct plant physiological responses (pointing to induced systemic resistance) rather than strong microbial shifts. This study highlights the importance of considering the biogeographic features shaping disease-suppressive soils and their microbiota-plant interactions.

**Supplementary Information:**

The online version contains supplementary material available at 10.1186/s13059-025-03911-0.

## Background

Certain plant pathogens cause extensive damage to crop roots, and they are notoriously difficult to control. However, in the soil system, these phytopathogens interact with the broader microbial community, and such interactions often limit the ability of soil-borne pathogens to infect roots. In certain soils, termed disease-suppressive soils, microbial interactions are sufficiently effective to protect plants significantly from specific soil-borne pathogens, thereby minimizing plant damage [1–3].

Specific disease suppressiveness may be due to particular pedo-climatic conditions, in which case it is typically maintained despite crop rotations and fluctuations in cropping practices [4–6], but with the possibility that the magnitude of suppressiveness may vary in time [6]. This suppressiveness has been documented for various diseases, including tobacco black root rot caused by the fungal pathogen *Thielaviopsis* (syn. *Berkeleyomyces*) *basicola* [7, 8], and Fusarium wilt diseases of flax [9], cape gooseberry [10] and wheat [11]. Both suppressive and non-suppressive (i.e. disease-conducive) soils typically coexist within the same landscape, resulting in significant differences in plant health between these soils despite similar climatic and agronomic conditions [5]. These differences in disease incidence are primarily attributed to particularities of the soil microbiota, but often the underlying differences in physicochemical soil properties that shape microbial communities and plant–microbe interactions are poorly understood. In addition, microbial fluctuations related to microbial biogeographic patterns are typically not considered. In this context, we hypothesized that plant–microbe interactions and natural disease suppressiveness likely depend on both geology (i.e. the type of rock substratum from which the soil is derived, so geology will determine the type of soil present and its physicochemical properties) and geography (i.e. field location in the landscapes or regions). The latter encompasses geographic variations regarding a given type of soil, the cropping system and microbial diversity [12].

This issue related to soil geology/geography is of prime relevance for natural suppressiveness to *T. basicola*-mediated black root rot of tobacco in Switzerland and France, for four main reasons. First, black root rot suppressiveness is documented for Swiss and French soils that are cambisols (i.e. same soil type), and soil properties associated to suppressiveness have been largely characterized [13–15]. Previous studies highlighted difference in clay mineralogy between cambisols derived from morainic deposits and those formed on sandstone bedrocks, leading to variation in iron availability in the rhizosphere [13, 16]. This highlights how geological differences drive pedological variations. Second, suppressiveness to black root rot has been identified in distinct geographic areas, such as the region of Morens in Switzerland [13] and two regions of Savoie (near Seyssel and Albens) in France [14]. Interestingly, the relationship between geology and suppressiveness varies: in Morens, morainic soils are suppressive and sandstone conducive, whereas the opposite pattern is observed in Savoie. This contrast likely arises from differences in the geological composition of morainic and sandstone materials between Switzerland and Savoie, leading to different microbiota [17–19]. Furthermore, the clay mineralogy of Savoie soils differs from that of Morens soils [13, 14]. Third, other factors beyond geology influence soil suppressiveness. For instance, cropping systems differ between Switzerland and Savoie: rotations are dominated by wheat in Switzerland and by maize in Savoie. Additionally, the role of tobacco in these rotations varies: tobacco is still cultivated in Morens, albeit in decline, whereas it has not been grown in Savoie for approximately 30 years. Fourth, the contrasting effects of morainic and sandstone soils on suppressiveness between the two regions suggest that distinct phytoprotection mechanisms are at play in Switzerland and Savoie. Accordingly, significant taxonomic differences in bacterial community were documented between Swiss soils [8, 15] but not between Savoie soils [14].

The objective was to test the hypothesis that plant–microbe interactions and natural disease suppressiveness depend on the geological origin and geographic positioning of the soils, using natural suppressiveness to *T. basicola*-mediated black root rot of tobacco in Switzerland and France. Thus, to test the relevance of geography and geology for plant–microbe interactions and suppressiveness, eight soils previously identified as suppressive or conducive were collected from Morens and Savoie and used to grow tobacco plants, either inoculated or not with the black root rot pathogen *T. basicola*. Disease severity levels were recorded to confirm soil suppressiveness status. Soil physicochemical data and soil metabolomics data were taken into account for a more comprehensive appraisal of soil conditions. In addition, microbial communities were characterized by metabarcoding and metagenomics to consider both taxonomic and functional particularities. Finally, plant metabolic profiles were determined to assess plant responses in relation to pathogen and suppressiveness conditions.

## Results

### Plant health analysis

**Fig. 1:**
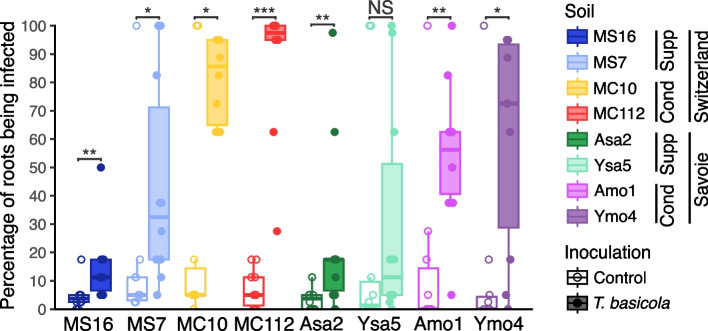
Black root rot severity for tobacco plants grown in the eight soils used in this study. Boxplots represent disease severity under control conditions (unfilled boxplots and circles) and *Thielaviopsis basicola* inoculated conditions (filled boxplots and circles). For each condition (*n* = 10 individual plants), disease severity was assessed three weeks after transplantation using a black root rot symptom scale representing the proportion of infected roots, ranging from 0% (no symptoms, white healthy roots) to 100% (dead plant). Data for Swiss soils originate from Harmsen et al*.* [15]. Disease severity in control conditions (mean ± standard error) was 4.50 ± 1.57 for MS16, 16.25 ± 9.44 for MS7, 24.25 ± 12.72 for MC10, 7.25 ± 2.15 for MC112, 3.37 ± 1.15 for Asa2, 21.87 ± 13.07 for Ysa5, 15.00 ± 9.91 for Amo1 and 12.50 ± 9.87 for Ymo4. In inoculated conditions, these values amounted to 15.13 ± 4.20 for MS16, 43.62 ± 11.60 for MS7, 81.13 ± 4.65 for MC10, 87.50 ± 7.58 for MC112, 25.13 ± 9.72 for Asa2, 31.25 ± 12.62 for Ysa5, 55.00 ± 8.24 for Amo1 and 60.38 ± 12.13 for Ymo4. For each soil, significant differences (Wilcoxon tests) between inoculated and control treatments are indicated by * (*P* < 0.05), ** (*P* < 0.01) or *** (*P* < 0.001). NS, not significant. For each soil, the suppressiveness status ('Cond' for conducive, 'Supp' for suppressive) and sampling region ('Savoie' or 'Switzerland') are indicated

Plant health was assessed for tobacco grown in the eight soils, with and without pathogen inoculation. For individual soils, black root rot severity reached 3–24% (based on means) and 0–5% (for medians) of infected roots in the absence of pathogen addition (Fig. 1). Disease severity was significantly higher when plants were inoculated with *T. basicola* for all soils (Wilcoxon tests, *P* < 0.05), except for Savoie sandstone soil Ysa5. In the Swiss soils, *T. basicola* inoculation increased mean disease severity from 4% to only 16% in MS16 (pointing to a suppressive status based on previous findings [5]), 16% to 43% in MS7 (moderately-suppressive status), but 24% to 81% in MC10 (conducive status) and 7% to 87% in MC112 (conducive status). In the Savoie soils, *T. basicola* inoculation enhanced mean disease severity from 3 to 25% in Asa2 and had no significant effect in Ysa5 (22% in the control and 31% after inoculation) (Fig. 1), pointing to a suppressive status [14]. In contrast, disease increased from 15 to 55% in Amo1 and 12% to 60% in Ymo4, in accordance with their conducive status [14]. Pathogen inoculation decreased (i) fresh root biomass in Swiss soils MS16 (by −77%; Wilcoxon, *P* = 1.8 × 10^–4^), MS7 (by −57%; *P* = 0.021) and MC112 (by −79%; *P* = 0.0076), and in conducive Savoie soil Ymo4 (by −65%; *P* = 0.013), (ii) dry root biomass in MS16 (by −61%; *P* = 0.0068), MC112 (by −72%; *P* = 0.036) and Ymo4 (by −72%; *P* = 0.018), (iii) the numbers of green leaves in MS16 (by −27%; *P* = 0.049), MC112 (by −69%; *P* = 0.0014) and Ymo4 (by −25%; *P* = 0.028), as well as (iv) the numbers of all leaves (by −71%; *P* = 6.9 × 10^–4^) and the proportion of plants alive (Chi-squared with Yate’s correction, *P* = 0.0024) only in the conducive Swiss soil MC112 (by −78%;) (Additional file 1: Figs. S1 and S2).

When considering all soils together, most plant health-related variables (i.e., black root rot severity, fresh and dry root biomass, fresh shoot biomass, numbers of all leaves and green leaves) varied significantly depending on individual soil, inoculation conditions and the interaction between the two (Kruskal–Wallis, all *P* < 0.05) (Fig. 1, Additional file 1: Figs. S1 and S2). This was also the case for (i) site geology, i.e. morainic or sandstone material, (ii) soil geography, i.e. Switzerland or Savoie (except for the proportion of plants alive and for dry root biomass), and (iii) the soil geography × geology interaction (except for dry root biomass) (Kruskal–Wallis, all *P* < 0.05). We found no significant differences due to site geology or suppressiveness status alone for any of the plant health data. All these plant health data were significantly correlated (Spearman correlation, with BH correction for multiple testing; Additional file 1: Fig. S2). In summary, the suppressiveness status of the 8 soils was confirmed, and we found significant effects of geology, geography and their interaction.

### Soil composition and soil metabolome analyses

The soils of the eight sites were characterized by standard physicochemical analyses and soil metabolome analysis. For physicochemical properties of the soils, the first two axes of the Principal Component Analysis (PCA; 72% of the variance, Additional file 1: Fig. S3A) separated conducive from suppressive soils, based on total nitrogen and organic matter contents. However, the overall differences in soil physicochemical profiles according to suppressiveness status (suppressive vs conducive), site geology or geography were not significant based on Permutational Analyses of Variances (PERMANOVA; *P* > 0.05). The first and third axes of the PCA (59% of the variance, Additional file 1: Fig. S3B) showed a separation between Swiss and Savoie soils, with significantly higher total iron contents in Savoie soils (Student’s *t*-test, *P* = 0.035). The first two axes of the Principal Least Square Discriminant Analysis (PLS-DA) accounted for 70% of the variance, and total N and organic matter contents (both significantly higher in conducive soils; *t*-tests with *P* = 0.026 for each; Additional file 1: Fig. S3D) contributed the most to the construction of the first axis (Additional file 1: Fig. S3C).

**Fig. 2:**
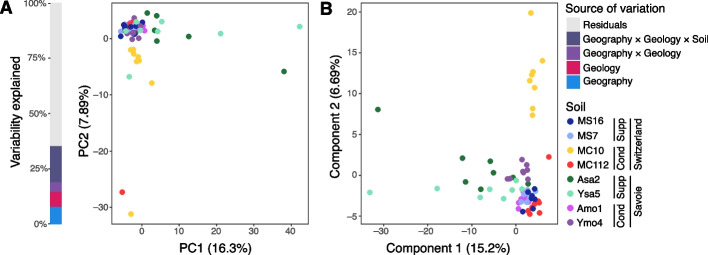
Principal Component Analysis (PCA) and Partial Least Square-Discriminant Analysis (PLS-DA) of soil metabolomes characterized from field soil used in the growth chamber experiment. Features obtained by GC–MS were used as variables. Each point represents a metabolome sample, colored by soil (*n* = 8 metabolome samples per soil). **A** PCA plot and stacked bar chart showing the percentage of metabolome variability explained by four sources of variation (computed by PERMANOVA): Geography (i.e. Switzerland vs Savoie), Geology (i.e. moraine vs sandstone bedrock), Geology × Geography, and Geography × Geology × Soil (i.e. the eight individual soils). The variability that could not be explained by these factors (65%) is represented in gray. **B** PLS-DA plot based on the metabolome matrix, supervised by Soil. For each soil, the suppressiveness status ('Cond' for conducive, 'Supp' for suppressive) and sampling region ('Savoie' or 'Switzerland') are indicated

Soil metabolomes depended significantly on the individual soils (PERMANOVA;* P* ≤ 1 × 10^–4^), and their geography (*P* ≤ 1 × 10^–4^), geology (*P* ≤ 1 × 10^–4^) and suppressiveness status (*P* = 0.0014) (Fig. 2, Additional file 2: Table S1). Most of the variation was explained by each soil individually (η^2^ = 16.4%), then by geography and geology (η^2^_Geography_ = 7.83%, η^2^_Geology_ = 6.81%; Fig. 2A, Additional file 2: Table S1), as well as suppressiveness status (η^2^_Status_ = 4.37%). The PLS-DA supervised by soil segregated samples according to geology in Savoie. The second axis (6.7% of the variance) separated Albens vs Seyssel within Savoie and also distinguished MC10 from the other Swiss soils. In summary, the soil metabolome was related to geography and suppressiveness status, as were N and organic matter contents.

### Metabarcoding of the fungal community in the tobacco rhizosphere

**Fig. 3:**
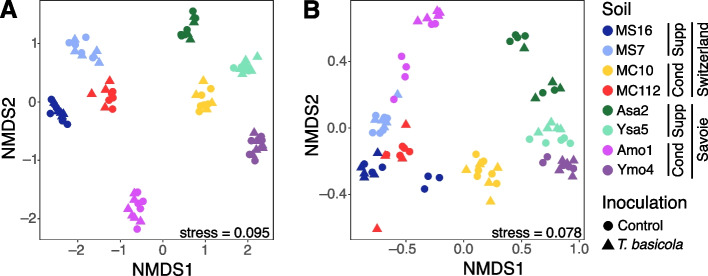
Non-metric MultiDimensional Scaling (NMDS) plots based on ITS (**A**) and 16S rRNA (**B**) amplicon sequencing data from rhizosphere DNA. Each sample is represented by a point; plants were grown in the eight soils (represented by different point colors) and either inoculated with *T. basicola* (triangle) or left as controls (circles). Stress values (indicating the quality of the ordination adjustment) are indicated. For each soil, the suppressiveness status ('Cond' for conducive, 'Supp' for suppressive) and sampling region ('Savoie' or 'Switzerland') are indicated

The fungal community in the tobacco rhizosphere was assessed by ITS metabarcoding. Both pathogen inoculation (PERMANOVA,* P* ≤ 1 × 10^–4^) and individual soils (*P* ≤ 1 × 10^–4^) had a significant effect on fungal community structure in the rhizosphere, but the individual soils themselves explained most of this variability (R^2^ = 0.68 vs 0.03) (Fig. 3A, Additional file 2: Table S2). The same analysis conducted without *Thielaviopsis* reads also showed a large, significant effect of the individual soils (R^2^_Soil_ = 0.70, *P* ≤ 1 × 10^–4^), but *P* for the inoculation effect was 0.051 only. Concerning our working hypothesis, the corresponding PERMANOVA models indicated that the effects of suppressiveness ‘Status’ (R^2^_Status_ = 0.09, *P* ≤ 1 × 10^–4^) and ‘Geology’ (R^2^_Geology_ = 0.11, *P* ≤ 1 × 10^–4^), as well as their interaction (R^2^_Geology × Status_ = 0.15, *P* ≤ 1 × 10^–4^) were significant, and the same was true for the effect of ‘Geography’ alone (R^2^_Geography_ = 0.15, *P* ≤ 1 × 10^–4^) or combined with ‘Geology’ (R^2^_Geography × Geology_ = 0.08, *P* ≤ 1 × 10^–4^). The correlation between geographic distance and fungal community dissimilarity was not significant (Mantel’s r = 0.28, *P* = 0.08), except when correcting by the soil’s physicochemical dissimilarity (partial Mantel’s r = 0.52, *P* = 0.028).

**Fig. 4:**
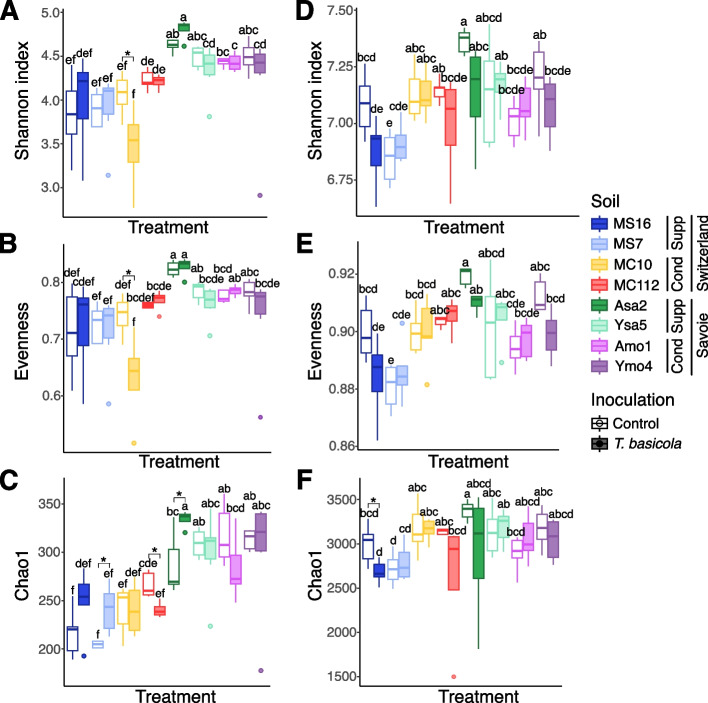
Diversity indices calculated from ITS (**A**, **B** and **C**) and 16S rRNA (**D**, **E** and **F**) amplicon sequencing data from rhizosphere DNA. Shannon diversity, Pielou’s evenness and Chao1 index were computed with R functions from the ‘vegan’ package. Boxplots (*n* = 4 to 6 plants) are colored according to the soil plants were grown. Filled boxes and circles represent samples with *T. basicola* inoculation. Letters a-f indicate statistical relationship between the 16 conditions (LSD tests, *P* < 0.05), while stars denote significant difference between inoculated samples and controls for any given soil, based on another analysis (Wilcoxon tests, *P* < 0.05). For each soil, the suppressiveness status ('Cond' for conducive, 'Supp' for suppressive) and sampling region ('Savoie' or 'Switzerland') are indicated

The analysis of alpha-diversity indices (Shannon’s diversity, Pielou’s evenness, and Chao1’s richness) computed using fungal ITS sequences showed that the effects of individual soils were significant (ANOVA with permutation test, 9,999 permutations, *P* ≤ 1 × 10^–4^ for all indices), as well as the interaction effect of ‘Soil’ and ‘Inoculation’ for Chao1 only (*P* = 0.022). When conducting the same analysis without *Thielaviospsis* reads, the same finding was observed for individual soils (PERMANOVA, *P* ≤ 1 × 10^–4^ for all indices) and the interaction between ‘Soil’ and ‘Inoculation’ for Chao1 (*P* = 0.023) and Pielou (*P* = 0.023). Pathogen inoculation resulted in higher Chao1 index in suppressive soil Asa2 (Wilcoxon tests, *P* = 0.038) and moderately-suppressive soil MS7 (*P* = 0.03), but lower Chao1 index in conducive soil MC112 (*P* = 0.016) and lower Shannon (*P* = 0.038) and Pielou indices (*P* = 0.038) in soil MC10 (Fig. 4A, B and C). All three indices were higher in Savoie soils than in Swiss soils.

**Fig. 5:**
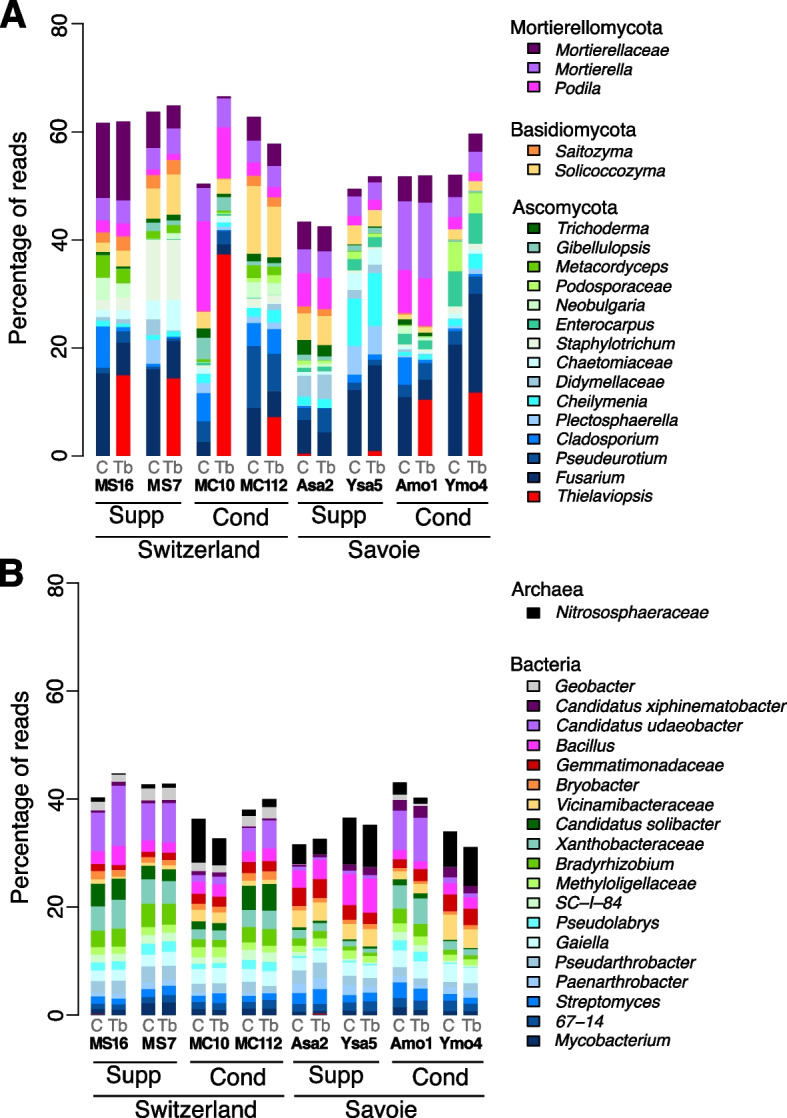
Relative abundance of taxa based on fungal (**A** ITS sequencing) and prokaryotic (**B** 16S rRNA gene sequencing) ASVs from rhizosphere DNA. The stacked barplots show the percentage of reads for the twenty most abundant taxa across the eight soils. On the X-axis, ‘Tb’ refers to samples inoculated with *T. basicola*, while ‘C’ denotes non-inoculated controls. For each soil, the suppressiveness status ('Cond' for conducive, 'Supp' for suppressive) and sampling region ('Savoie' or 'Switzerland') are indicated

The fungal community was dominated by *Ascomycota*, *Basidiomycota* and *Mortierellomycota* (Fig. 5A). Geography and suppressiveness status of the soil did not have a significant effect on the relative amount of *Thielaviopsis* reads, but the inoculation treatment did (Kruskal–Wallis tests, *P* = 4.78 × 10^–15^). In inoculated soils (but not in non-inoculated soils), the number of *Thielaviopsis* reads was significantly higher in conducive soils than in suppressive soils in Switzerland (Kruskal–Wallis test, *P* = 0.0015) but not in Savoie. The number of *Thielaviopsis* reads was significantly higher after inoculation than in the control (Wilcoxon test, all *P* < 0.05) in every soil except suppressive soil Asa2. We also found that the number of *Thielaviopsis* reads correlated to disease severity (Spearman correlation on ranks; *ρ* = 0.63, *P* = 1.02 × 10^–10^). With non-inoculated soils, we identified 35 fungal taxa indicative of suppressiveness in Savoie (i.e. more prevalent in all suppressive soils than in all conducive soils; Additional file 1: Fig. S4A) and 18 in Switzerland (Additional file 1: Fig. S4C), but none were common to both (Additional file 1: Fig. S4C). The orders most represented among these indicators were the *Pleosporales*, *Pezizales*, *Hypocreales* and *Glomerellales* in Savoie soils and the *Helotiales* and *Pleosporales* in Swiss soils. In inoculated soils, 39 suppressiveness indicator taxa were found for Savoie and 13 others for Switzerland, especially among the *Pleosporales*, *Hypocreales* and *Sordariales* (Additional file 1: Fig. S4). Within Savoie, 28 suppressiveness indicator taxa were evidenced in both control and inoculated conditions, plus 11 and 7 others unique to inoculated conditions or to the controls, respectively. For the Swiss soils, there were 10 suppressiveness indicators common to inoculated and control conditions, plus 4 unique to inoculated conditions and 6 to the controls. In summary, individual soils mattered most, yet Geography, Geology, and Status (suppressiveness) had a significant effect on fungal community structure but not on diversity or taxonomic composition, and indicator taxa (all country-specific) of suppressiveness were identified.

### Metabarcoding of the bacterial and archaeal communities in the tobacco rhizosphere

The prokaryotic community in the tobacco rhizosphere was assessed by 16S rRNA gene metabarcoding. Individual soils (‘Soil’ effect), but not inoculation treatment (‘Inoculation’), had a significant effect on the prokaryotic community in the rhizosphere (PERMANOVA, *P* ≤ 1 × 10^–4^, R^2^ = 0.63), and the interaction between ‘Soil’ and ‘Inoculation’ was significant (R^2^ = 0.05 and *P* = 0.049; Fig. 3B, Additional file 2: Table S3). However, when considering each soil separately, the ‘Inoculation’ effect was significant for MS16 (R^2^_Inoculation_ = 0.17, *P* = 0.034), Ysa5 (R^2^_Inoculation_ = 0.15, *P* = 0.046) and Amo1 (R^2^_Inoculation_ = 0.17, *P* = 0.030). We also observed differences between (i) suppressive and conducive soils (R^2^_Status_ = 0.05, *P* = 9 × 10^–4^), (ii) moraine and sandstone soils (R^2^_Geology_ = 0.10, *P* ≤ 1 × 10^–4^) and (iii) Swiss and Savoie soils (R^2^_Geography_ = 0.19, *P* ≤ 1 × 10^–4^). Additionally, the interaction between suppressiveness status and soil geology had a significant impact (R^2^_Geology × Status_ = 0.19, *P* ≤ 1 × 10^–4^), as did site geography and geology (R^2^_Geography × Geology_ = 0.05, *P* = 2 × 10^–4^). The simple Mantel test revealed no significant correlation between geographic distance and prokaryotic community dissimilarity in soils (Mantel’s r = 0.28, *P* = 0.086) but the partial Mantel test (correcting for differences in physicochemistry between soils) did show a significant relationship (partial Mantel’s r = 0.53, *P* = 0.031).

As with the fungal community, the alpha diversity of the prokaryotic community (16S rRNA gene) was not necessarily influenced by the inoculation treatment. When inoculation had an effect (Wilcoxon test), it resulted in lower diversity for MS16 (Chao1 index, *P* = 0.030; Fig. 4D, E and F).

The archaeal community was dominated by *Nitrososphaerota* and the bacterial community by *Actinomycetota*, *Pseudomonadota*, *Acidobacteriota*, *Bacillota*, *Gemmatimonadota* and *Verrucomicrobiota* (Fig. 5B). We found 14 prokaryotic taxa indicators of suppressiveness in both non-inoculated and inoculated Savoie soils (out of 37 and 31 indicator taxa, respectively) (Additional file 1: Fig. S5). In Swiss soils, there were 10 and 13 suppressiveness indicator taxa unique to the control or inoculated conditions, respectively, with 4 others common to both. Therefore, only *Bosea* was indicative of suppressiveness in both Savoie and Swiss soils (but solely under non-inoculated conditions; Additional file 1: Fig. S5). In summary, individual soils had the largest effect on prokaryotic community data, with yet a significant effect of Geography, Geology, and Suppressiveness status on prokaryotic community structure but not diversity or taxonomic composition, and suppressiveness indicator taxa (mostly country-specific) were found.

### Metagenome-based taxonomy and functional potential of the tobacco rhizosphere microbiota

**Fig. 6:**
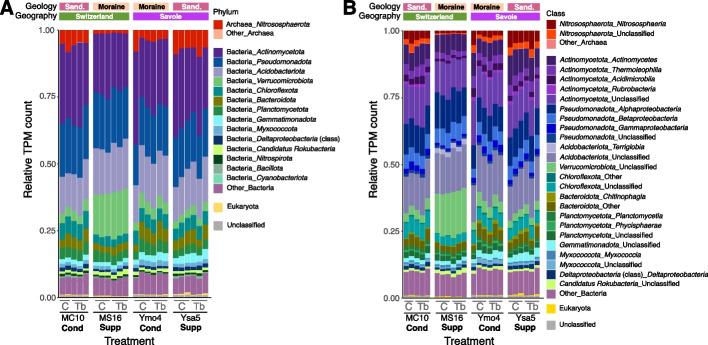
Stacked barplots showing relative TPM values for taxonomic groups in the metagenome. TPM values were calculated using SCGs (as described [20]) by summing the TPMs for genes assigned to SCGs and grouping them by (**A**) phylum and (**B**) class. Archaeal taxa are displayed in red and pink, bacterial taxa in purple, blue, and green, and eukaryotic taxa in yellow. The 'Unclassified' category includes genes not assigned to the analyzed taxonomic levels. Top bar annotations indicate 'Geology' (soil developed on moraine or sandstone) and 'Geography' (soil sampled in Savoie or Switzerland). Bottom bar annotations specify ‘Inoculation’ (C for control and Tb for inoculated *T. basicola*) and ‘Status’ ('Supp' for suppressive and 'Cond' for conducive)

The tobacco rhizosphere microbiota was assessed by metagenomics. At least 89% of the Transcripts Per Million (TPMs) were Single-Copy Genes (SCGs) assigned to bacteria especially *Actinomycetota* (28% of bacteria), *Acidobacteriota* (17%) and *Pseudomonadota* (20%) (Fig. 6). Many taxonomic groups, but not the same in Savoie and Switzerland, differed in abundance between suppressive and conducive soils (Fig. 6, Additional file 2: Tables S4 and S5).

**Fig. 7:**
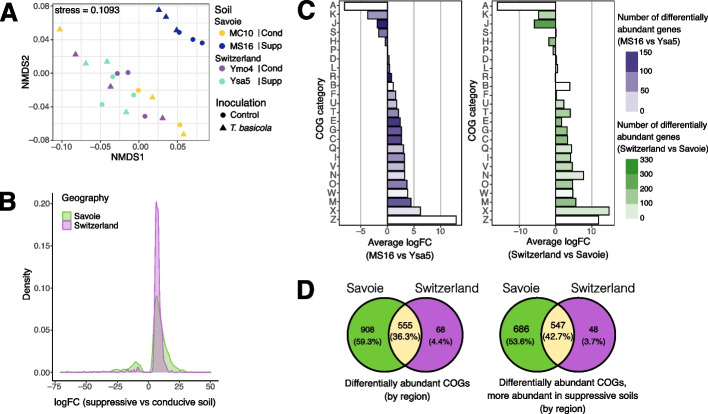
Functional analysis of the tobacco rhizosphere metagenomes. **A** NMDS plot based on COG relative abundance (TPM counts) in the rhizosphere metagenome. Each point represents a sample, with colors indicating the four soils and shapes showing inoculation conditions: circles for controls and triangles for *T. basicola*-inoculated samples. For each soil, the suppressiveness status ('Cond' for conducive, 'Supp' for suppressive) and sampling region ('Savoie' or 'Switzerland') are indicated. **B** PERMANOVA results assessing the functional potential of the rhizosphere community. PERMANOVA was performed on read counts summed by COGs. Models included the effects of ‘Geography’ (Savoie or Switzerland), ‘Inoculation’ (*T. basicola* added or not), ‘Status’ (conducive or suppressive soil) and ‘Geology’ (moraine or sandstone). Significant effects (*P* < 0.05) are highlighted with bold text and stars: (*) for *P* < 0.05, (**) for *P* < 0.01, (***) for *P* < 0.001. ‘R2’ represents the proportion of explained variability, and ‘F’ the pseudo-F statistic. **C** Barplot showing the average log fold change (logFC) of genes in core COG functional categories. Comparisons include (left) the Swiss suppressive soil (MS16) vs Savoie suppressive soil (Ysa5), and (right) Switzerland vs Savoie soils. Bars are ordered by increasing MS16 vs Ysa5 logFC, with colors representing the number of differentially abundant (DA) genes per core COG category (calculated using 'metagenomeSeq'). **D** Venn diagrams of DA COGs in suppressive vs conducive soils for each region. Diagrams show (i) all DA COGs and (ii) only those more abundant in suppressive soils

The total number of unique ORFs assigned to a COG was 3,389,800. From that, we identified 4,539 COGs in the co-assembled metagenome, when considering all samples together. The overall functional composition of the samples, as defined by the abundance for each COG, varied significantly (PERMANOVA) with ‘Geography’ (R^2^_Geography_ = 0.23, *P* = 5 × 10^–4^), ‘Suppressiveness status’ (R^2^_Status_ = 0.13, *P* = 0.0076), and the ‘Geography × Status’ interaction (R^2^_Geography × Status_ = 0.15, *P* = 0.0040) (Additional file 2: Table S6). Using ‘Geography × Geology × Inoculation’ model, the functional profiles were significantly influenced by ‘Geography’ (R^2^ = 0.23, *P* = 3 × 10^–4^), ‘Geology’ (R^2^ = 0.15, *P* = 0.0053), and ‘Geography × Geology’ (R^2^ = 0.14, *P* = 0.0064). A clear separation between Swiss soils was observed on the Non-metric MultiDimentional Scaling (NMDS) plot, but not for the Savoie soils, which grouped with conducive Swiss soil MC10 (Fig. 7A). Mantel test indicated a correlation between geographic distance and dissimilarity of rhizosphere functional profiles (Mantel’s r = 0.83, *P* = 0.041). Pathogen inoculation alone or combined with other factors had no significant impact on functional profiles (Fig. 7B).

There were 1,463 COGs differentially abundant between Savoie soils Ysa5 (suppressive) and Ymo4 (conducive), across 24 of the 26 main COG categories (Additional file 2: Table S7), and 1,233 of them were more abundant in Ysa5. For the Swiss soils, we found only 623 differentially-abundant COGs between MS16 (suppressive) and MC10 (conducive) (615 of them overrepresented in MS16), but with generally higher log fold-changes (Fig. 7B). A total of 555 differentially-abundant COGs (for suppressive vs conducive soils) were common to Savoie and Switzerland (Fig. 7D), 547 of them overrepresented in suppressive soils. We also identified, in the same 24 categories, 1487 and 3121 differentially-abundant COGs between (i) suppressive soils MS16 and Ysa5 and (ii) all Savoie soils and all Swiss soils, respectively (Fig. 7C, Additional file 2: Table S7).

Since secondary metabolites are key for disease control, we focused on the Secondary metabolites biosynthesis, transport and catabolism (Q) COG category. It was more represented in suppressive soils for both Savoie (logFC = + 2.28, *P* = 3.67 × 10^–6^) and Swiss soils (logFC = + 2.19, *P* = 5.52 × 10^–8^). Spearman’s correlation coefficient between soil clay content and the abundance of COGs belonging to the Q category reached 1 (Additional file 1: Fig. S6). COGs more abundant in suppressive vs conducive soils (Wilcoxon rank sum tests, FDR correction) amounted to 35 in Switzerland (including protein domains involved in polyketide biosynthesis [e.g. COG3321, COG3315 and COG3319] or non-ribosomal peptide synthesis [e.g. COG3319, COG1020, COG1021]) and 7 COGs in Savoie (Additional file 1: Fig. S7). Four of these COGs were common to both regions, i.e. COG3960 (glyoxylate carboxylase), COG5490 (polyhydroxyalkanoate inclusion-associated protein PhaP/PhaF, phasin family), COG3315 (O-methyltransferase for polyketide biosynthesis), and COG1775 (benzoyl-CoA reductase/2-hydroxyglutaryl-CoA dehydratase subunit, BcrC/BadD/HgdB). COGs overrepresented in suppressive vs conducive soils, (i) both in Savoie and Switzerland gave a Spearman correlation coefficient of −1 with CEC (COG3960, glyoxylate carboligase), (ii) only in Switzerland a ρ value of −1 with CEC (3 COGs), total iron content (5 COGs), organic matter and total nitrogen contents (3 COGs), clay content (3 COGs), P content, CEC saturation and pH (7 COGs), and (iii) only in Savoie a ρ value of 1 with P content, CEC saturation and pH (COG3486, lysine/ornithine *N*-monooxygenase; COG1535, isochorismate hydrolase), total iron content (COG3320, thioester reductase domain of alpha aminoadipate reductase Lys2 and NRPSs) (Additional file 1: Fig. S6).

The Q COG category was overrepresented in Swiss soils compared with Savoie soils, both for all soils (logFC_Geography_ = + 4.43, *P* = 9.68 × 10^–8^) and suppressive soils only (logFC_Geography_ = + 3.01, *P* = 2.74 × 10^–7^), with 68 and 7 differentially-abundant COGs, respectively (Additional file 2: Table S8). Among these seven COGs, six were more abundant in MS16 than Ysa5, namely COG4181 (predicted ABC-type transport system involved in lysophospholipase L1 biosynthesis, ATPase component), COG2366 (acyl-homoserine lactone acylase PvdQ), COG4242 (cyanophycinase and related exopeptidases), COG3320 (thioester reductase domain of alpha-aminoadipate reductase Lys2 and Non-Ribosomal Peptide Synthetases [NRPSs]), COG1942 (phenylpyruvate tautomerase PptA, 4-oxalocrotonate tautomerase family) and COG4829 (muconolactone delta-isomerase), and one was less abundant, i.e. COG4457 (virulence factor SrfB-related protein) (Additional file 2: Table S8).

In summary, rhizosphere community metabolic potential depended on geographic positioning and geological origin of the soil, but suppressiveness status was also influential (especially in Switzerland). Also, COG annotation identified protein domains overrepresented in suppressive vs conducive soils (mostly region specific), notably for functions in the COG Q category (e.g. linked to NRPS, polyketide synthesis).

### Distribution and functional potential of MAGs in the tobacco rhizosphere

Metagenomic data from the tobacco rhizosphere were also assessed by considering reconstructed consensus genomes. Out of the 301 bins, we obtained 52 Metagenome-Assembled Genomes (MAGs) that were > 50% complete and < 10% contaminated (Additional file 2: Table S9). The main phyla represented were the *Actinomycetota* (12/52), *Pseudomonadota* (11/52), *Acidobacteriota* (7/52), *Bacteroidota* (6/52) and *Verrucomicrobiota* (4/52) (Additional file 1: Fig. S8, Additional file 2: Table S9). Twenty of them were more abundant (all *P* < 0.05; Wilcoxon rank sum test, FDR corrected) in Swiss suppressive vs conducive soil, and 16 in Savoie suppressive vs conducive soil, with 4 MAGs in common (1 *Arthrobacter*, 2 *Actinomycetes,* and 1 *Chloroflexota*).

In the MAGs, 5 of 87 Q-category COGs were significantly overrepresented in suppressive soils for both Switzerland and Savoie, including COG3315 (O-methyltransferase) and COG3321 (acyl transferase domain) linked to polyketide synthesis, and COG0146 (*N*-methylhydantoinase B/oxoprolinase/acetone carboxylase, alpha-subunit), COG1233 (phytoene dehydrogenase-related protein) and COG2015 (alkyl sulfatase BDS1 and related hydrolases, metallo-beta-lactamase superfamily). Some were the most abundant in MAGs enriched in suppressive soils for both regions, including 2 *Acidobacteriota* and 1 *Gemmatimonadota* for COG3315, 1 *Actinomycetes* and 1 *Actinomycetales* for COG1233, and 1 *Candidatus Rokubacteria* for COG0146. We found respectively 28 and 13 Q-category COGs overrepresented only in Swiss or in Savoie suppressive vs conducive soils (Additional file 1: Fig. S9).

**Fig. 8:**
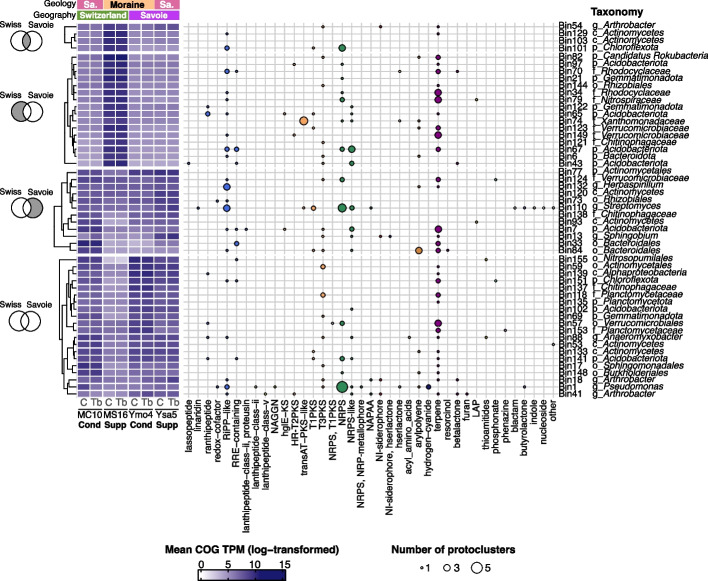
Heatmaps displaying (i) MAG abundance in the metagenome and (ii) the number of protoclusters linked to secondary metabolite production (summed by type), as predicted by antiSMASH. The heatmaps are divided into four sections based on whether MAGs were significantly more abundant (*P* < 0.05, Wilcoxon tests) in suppressive vs conducive soils in Savoie, Switzerland, both regions, or in neither (as shown in the Venn diagrams to the left; gray area indicates the category). MAG abundance is calculated as the average COG TPMs per treatment (soil and inoculation), log-transformed to minimize bias from high TPM values. MAGs are clustered according to these values, within each section. Annotations on the top of the heatmap correspond to 'Geography' (soil sampling region) and 'Geology' (soil geological origin, with ‘Sa.’ for sandstone). Annotations on the bottom of the heatmap refer to plant inoculation condition ('Tb' for *T. basicola*, 'C' for control), soil name, and soil status ('Supp' for suppressive, 'Cond' for conducive). Circle sizes on the right heatmap represent the number of protoclusters, and colors follow the antiSMASH color code. MAG taxonomy (lowest available level) is shown to the right of the heatmap

antiSMASH annotation did not reveal clear patterns for major protocluster types, but some minor protocluster types were found exclusively in MAGs more abundant in suppressive soils, i.e. lasso peptides, linear arid peptides, proteusins, heterocyst glycolipid synthase-like PKS (hglE-KS), trans-ATP PKS, linear azol(in)e-containing peptides (LAP), indoles, nucleosides, and β-lactams (Fig. 8). We also identified biosynthetic genes in MAGs more abundant in suppressive soils (Additional file 2: Table S10), e.g. for antifungal compounds (like laxaphycin lipopeptide, plipastatin, clavulanic acid or ε-poly-L-lysine), elicitor-like lipopolysaccharides and siderophores (like staphyloferrin B or desferrioxamine E).

In summary, we identified in MAGs several protein domains (i.e. COGs) overrepresented in suppressive vs conducive soils and found that MAGs that were more abundant in suppressive soils harboured functions potentially linked to suppressiveness.

### Secondary metabolism for prominent MAGs

*Pseudomonas* bacteria play a key role in Swiss suppressive soils [5]. The *Pseudomonas* MAG obtained (‘Bin 1’) was the only MAG that passed the quality thresholds (completeness > 90% and contamination < 5%), and was further investigated. This MAG was less abundant (−23%) in Swiss suppressive soil MS16 compared with conducive soil MC10 (Wilcoxon test with FDR correction, *P* = 0.0094), without any difference between the Savoie conducive and suppressive soils. This MAG corresponded to *Pseudomonas kilonensis* (TYGS analysis), with a dDDH d_4_ of 89% with type-strain *P. kilonensis* DSM 13647 (Additional file 1: Fig. S10, Additional file 2: Table S11). antiSMASH identified 7 putative protoclusters, each containing at least 25% genes matching known sequences in the database (Additional file 2: Table S10). These included 4 NRPS or NRPS-like clusters, predicted to encode antifungal compounds bicornutin A1/A2 [21] (100% similarity) and fragin [22] (37% similarity), as well as the siderophores cepaciachelin [23] (25% similarity) and azotobactin D [24] (41% similarity). Additionally, one cluster associated with hydrogen cyanide (100% similarity) synthesis, one type III polyketide synthase (T3PKS) cluster involved in biosynthesis of 2,4-diacetylphloroglucinol (DAPG; 100% similarity), and one arylpolyene cluster linked to antifungal APE Vf [25] production (40% similarity) were identified.

As suppressiveness in the Savoie soils is very poorly understood, a focus was put on prominent MAGs considering antiSMASH data and taxonomic resolution (at the genus level). Of the 12 MAGs that were more abundant in suppressive soils in Savoie only, ‘Bin 110’ (genus *Streptomyces*) was particularly interesting as it harboured antiSMASH cluster types not found in any of the other MAGs (‘β-lactam’, ‘indole’, ‘nucleoside’). It harboured 19 clusters linked to the production of secondary metabolites, including antifungals like clavulanic acid (33% similarity) and ε-poly-L-lysine (100% similarity; Additional file 2: Table S9). The most abundant Q-category COGs were COG3321 (linked to polyketide biosynthesis, as stated earlier), COG5517 (3-phenylpropionate/cinnamic acid dioxygenase, small subunit) and COG2124 (cytochrome P450, within the ‘biotin biosynthesis’ COG pathway). Another prominent MAG was ‘Bin 13’ (*Sphingobium* genus), annotated to a relatively low taxonomic level (genus) but that harboured secondary metabolite clusters of interest. It was annotated with five clusters detected by antiSMASH, including one for the carotenoid zeaxanthin (100% identity) and another for the siderophore staphylopherrin B (16% identity) (Additional file 2: Table S9). The domain COG5285 (ectoine hydroxylase-related dioxygenase, phytanoyl-CoA dioxygenase family) was particularly abundant in this MAG, and it was significantly overrepresented in MAGs when considering Savoie suppressive soil (Additional file 1: Fig. S8).

In summary, we identified a *P. kilonensis* MAG (with prominent secondary metabolism) specific to Swiss soils. Among the 12 MAGs more abundant in suppressive Savoie soils, two prominent genomes (a *Streptomyces* MAG and a *Sphingobium* MAG) stood out due to the presence of biosynthetic gene clusters for antifungal compounds and/or siderophores.

### Shoot metabolomics analysis

**Fig. 9:**
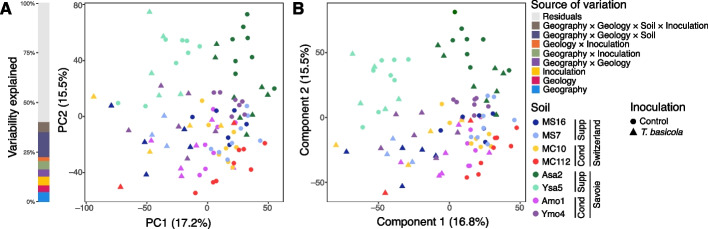
Principal Component Analysis (PCA) and Partial Least Square-Discriminant Analysis (PLS-DA) of the metabolomes of tobacco shoots. Features obtained by LC–MS were used as variables. Each point represents a metabolome sample, colored by soil (*n* = 8 metabolome samples per soil) and with symbol shapes indicating the treatment (triangle for plants inoculated with *T. basicola* and circles for controls). **A** PCA plot and stacked bar chart representing the percentage of metabolome variability explained by eight sources of variation (as computed by PERMANOVA). The remaining variability (60%) is represented in gray. **B** PLS-DA with the metabolome matrix, supervised by ‘Soil’ and ‘Inoculation’. For each soil, the suppressiveness status ('Cond' for conducive, 'Supp' for suppressive) and sampling region ('Savoie' or 'Switzerland') are indicated

The shoot metabolome of tobacco varied with individual soils (PERMANOVA, *P* ≤ 1 × 10^–4^), soil geography (*P* ≤ 1 × 10^–4^), geology (*P* = 2 × 10^–4^), and *T. basicola* inoculation (*P* ≤ 1 × 10^–4^) (Fig. 9, Additional file 2: Table S12). Accordingly, data variation was linked significantly to the individual soil (η^2^ = 12.6% of variance explained in the PERMANOVA), soil geography (5.11%), inoculation condition (4.34%), and geology (3.38%) (Fig. 9A, Additional file 2: Table S12).

With PLS-DA supervised by individual soil and inoculation treatment, the second component of the PLS-DA (which explains 15% of the variance; Fig. 9B) segregated tobacco samples according to the individual soils, especially with the Savoie sandstone soils Ysa5 and Asa2 in comparison with the other Savoie soils (Ymo4, Amo1) as well as all Swiss soils. Accordingly, the Bray–Curtis dissimilarity between suppressive and conducive soils was significantly greater for the Savoie soils than the Swiss soils (0.302 vs 0.283; *P* = 6 × 10⁻^5^; 99,999 permutations). The chemical annotation of differentially accumulated compounds in tobacco shoots pointed to two nicotinic derivatives, i.e. *N*-formylnornicotine (C_10_H_12_N_2_O; [M + H]^+^ = 177.1023) and *N*-methylanabasine or homonicotine (C_11_H_16_N_2_; [M + H]^+^ = 177.1390), which were less accumulated in plants grown in Savoie suppressive soils compared with Savoie conducive soils (*P* < 0.05). In contrast, contents in nicotinic derivatives did not differ significantly in suppressive vs conducive soils in Switzerland. For all soils, the separation along the first axis (17% of the variance explained) contrasted samples according to the inoculation condition.

## Discussion

Soil suppressiveness is an outstanding emerging property, i.e. involving synergistic interactions between plant-protecting microorganisms, in terms of plant health and agricultural system sustainability [5]. It is receiving considerable research attention in relation to soil physicochemistry [3, 26] and microbial taxa and functions underpinning disease suppressiveness [7, 8, 15, 27, 28]. Suppressive soils have been documented here and there, but their biogeography is poorly understood. Here, we used the case of soils naturally suppressive to *Thielaviopsis*-mediated black root rot disease to test the hypothesis that plant–microbe interactions and suppressiveness depend both on geology-related site-specific physicochemistry and geographic location of fields.

To this end, a prerequisite was to confirm the plant health status of the soils, and growth chamber data validated the suppressive or conducive status of Swiss and Savoie soils. This was indicated by a small effect of *T. basicola* inoculation on tobacco health (i.e. small or insignificant increase in symptoms) in the four suppressive soils, and conversely by a significant pathogen effect in the four conducive soils (along with negative effects on root and shoot biomass). This is in accordance with past [6, 8, 14, 21] and recent findings [15], and highlights the importance of soil features for suppressiveness to black root rot.

The hypothesis of geologic and geographic effects on suppressiveness was assessed in terms of soil physicochemistry, microbiota taxonomic diversity (metabarcoding) and functional potential (metagenomics), and plant physiological responses (shoot metabolomics). First, the soils studied in the two regions were characterized as cambisols and had similar physicochemistry overall, which is consistent with previous work [14, 21]. However, despite these similarities, we identified a few physicochemical properties that differed between soils according to (i) soil suppressiveness status, i.e. lower organic matter content in suppressive soils than in conducive soils (documented already [6, 14]), and (ii) soil geography, i.e. higher total iron content in Savoie than in Switzerland. In contrast, the soil metabolome was structured by suppressiveness status, geology and geography. This may implicate inter-regional differences in soil management practices and crop rotation, with likely effects on the soil microbial community [29]. In the Swiss conditions, iron bioavailability promoted transcription of DAPG biosynthetic genes [16], HCN biosynthesis and black root rot biocontrol [30] by the Morens isolate *Pseudomonas protegens* CHA0, but here the differences found between soils concerned their contents in total iron. The lower organic matter content of suppressive soils contrasts with negative effects of organic matter applications on *T. basicola* soil populations [31], and *T. basicola* populations (by qPCR) were not lower in the tobacco rhizosphere of Swiss suppressive soils in comparison with conducive soils [32]. Therefore, effects mediated by the rest of the microbial community, on fungal infection and/or the expression of plant defense mechanisms, are likely. In short, we identified an impact of both geography and geology on soil physicochemical properties.

Second, we considered the taxonomic diversity of the rhizosphere microbiota, and found that it differed mainly according to the individual field, and to a lesser extent geography, geology and suppressiveness status. Despite the effect of geography (i.e., Savoie vs Switzerland), the differences were not correlated to spatial distance (simple Mantel tests) unless the test was corrected by physicochemistry dissimilarity between soils (partial Mantel tests), indicating a microbial community shaped by environmental factors (e.g. geology and associated soil physicochemical variations) rather than dispersal alone. Geology on its own had a bigger role in structuring the rhizosphere community than suppressiveness status, which suggests that mineral characteristics [33] mattered more than plant health conditions for root selection of the rhizosphere microbial community. For some suppressive soils, the higher fungal Chao1 diversity index in the rhizosphere of inoculated vs non-inoculated plants suggests that rare species are enriched when inoculating plants with *T. basicola*, perhaps because the inoculant limited dominance effects of other taxa. Some of the suppressiveness indicator taxa contained strains with plant-beneficial traits, including *Bosea* [34], a genus recruited by the plant in the presence of a phytopathogen [35] and here the only suppressiveness indicator common to both Savoie and Swiss soils. Thus, both geography and geology effects were meaningful, and we identified taxonomic microbial markers of suppressiveness that complemented the original focus on *Pseudomonas* [6, 7] and additional taxa pointed by 16S microarray analysis [8].

Third, the functional potential of the rhizosphere microbiota evidenced by metagenomics was linked to soil suppressiveness status and geology. The differences in functional diversity were much more pronounced for Swiss soils (Fig. 7A) than for Savoie soils. This suggests that functional differences resulting from local geological variations could play a prominent role in plant–microbe interactions and suppressiveness in the case of Switzerland. In addition, there was a clear difference in the link between soil physicochemistry and secondary metabolism-related COGs overrepresented in suppressive soils compared with conducive soils, when considering Savoie only (vs Switzerland only or both regions together). This is consistent with regional features, e.g. total iron content being more abundant in Savoie than Swiss soils. It may shape soil microbial communities [12, 36] and thus the microbial guilds responsible for disease suppression [37, 38]. The metagenomic data also gave new insights into potential mechanisms in suppressive soils. MAGs more abundant in suppressive soils harbored gene clusters associated to properties relevant for biocontrol, i.e. (i) the siderophores staphyloferrin B [39] (in a *Sphingobium* MAG) and desferrioxamine E [40] (*Arthrobacter* MAG), (ii) lipopolysaccharides (*Bacteroidales* MAG), which might elicit plant defenses [41], (iii) antifungal heinamides of the laxaphycin lipopeptide family [42] (cluster in an *Acidobacteriota* MAG), (iv) antifungal plipastatin [43] (*Rhodocyclaceae* MAG), (v) antifungal clavams [44, 45] (including clavulanic acid, in a *Streptomyces* MAG) and (vi) antifungal ε-poly-L-lysine [46] (same *Streptomyces* MAG) (Additional file 2: Table S10). The genera *Streptomyces* and *Sphingobium*, for which two MAGs were more abundant in Savoie suppressive vs conducive soils, include plant-beneficial strains [47, 48] that may contribute to disease suppression (including *Streptomyces* strains reported to inhibit *Thielaviopsis* species [49]). In addition to the potential for antifungal compound production identified in the *Streptomyces* MAG, we found a siderophore-associated gene cluster in the *Sphingobium* MAG, which might contribute to phytoprotection [50]. We also identified a *P. kilonensis* MAG that contained gene clusters for production of DAPG, a polyketide antifungal compound linked to soil suppressiveness in Swiss soils [51]. This MAG concerned both conducive and suppressive soils in Switzerland and Savoie, in accordance with the occurrence of a wide range of DAPG-producing *Pseudomonas* in both [14, 21]. Gene expression conditions differ in suppressive vs conducive soils, and it is thought to account for suppressiveness conditions in Swiss soils [51]. In addition, polyketide biosynthesis was identified as a key factor in suppressiveness through metagenomic COG annotation: COG3315 and COG3321, both associated with polyketide synthesis, were significantly more abundant in the rhizosphere of suppressive soils in both Savoie and Switzerland datasets, as shown by whole metagenome and MAG analyses. The *P. kilonensis* MAG also contained gene clusters related to HCN production, an important biocontrol compound for *Pseudomonas* suppression of tobacco black root rot [52]. In brief, we identified an impact of both geography and geology, and found functional microbial patterns associated with suppressiveness, which will need to be explored using a metatranscriptomics approach.

Fourth, plant responses were monitored through shoot metabolomics. Metabolomic profiles varied depending on geography, geology, soil suppressiveness status, and pathogen inoculation. These findings suggest complex interactions between environmental factors (including geology) and rhizosphere microbial communities, which in turn influence soil suppressiveness and consequently plant metabolic response to disease pressure. Unlike in Swiss soils, plants grown in Savoie suppressive vs conducive soils exhibited clearly different physiological profiles (Fig. 9). This observation raises the possibility that suppressiveness mechanisms in Savoie soils might involve plant-mediated processes, such as the elicitation of induced systemic resistance [53] pathways in tobacco. A similar phenomenon was previously observed with the Morens biocontrol strain *P. protegens* CHA0 [54], which successfully controlled black root rot without directly affecting *T. basicola* mycelium [55]. Here, the nicotinic derivatives *N*-methylanabasine (or homonicotine) and *N*-formylnornicotine were less prevalent in tobacco metabolomes in Savoie suppressive soils compared with conducive soils (but not in Switzerland). Since these compounds are involved in plant protection from oxidative stress and possibly (via the phenylalanine ammonia lyase) from pathogens [56–58], it suggests that tobacco plants in suppressive soils were less exposed to stress related to pathogen attack. More importantly, lower nicotinic derivatives relieve repression of the jasmonic acid/ethylene signaling pathway (which underpins induced systemic resistance) at the expense of the salicylic acid-mediated signaling pathway, at least in *Arabidopsis thaliana* [58], pointing further to a role of induced systemic resistance in suppressiveness of Savoie soils. In summary, we found that geography and geology, as well as soil suppressiveness status, influenced the metabolic responses of tobacco, with a possible contribution of induced systemic resistance to the suppressiveness of Savoie soils (which deserves further research attention).

## Conclusions

To decipher the biogeography of soil suppressiveness to disease, we leveraged previous investigations at a small geographic scale (< 200 km) in Switzerland [6, 7] and Savoie [14] that identified the occurrence of black root rot-suppressive soils according to the moraine or sandstone nature of the soil substratum, with contrasting relationships observed in the two regions. This enabled us to disentangle the respective impacts of geology (i.e. the type of rock substratum from which the soil is formed) and suppressiveness, and here we clarified the relationship between geographic location, geological origin, and soil suppressiveness. We validated the hypothesis that disease suppressiveness is a property determined by both geography and geology, which have an impact on the soil microbiome and plant–microbe interactions. Findings also highlight distinct suppressive mechanisms in the two regions, as suppressiveness in Swiss soils was primarily associated with differences in taxonomic and functional diversity of the rhizosphere microbiota rather than in plant physiology. In contrast, microbial communities in Savoie soils displayed fewer identifiable differences, but plant physiological responses were significantly more variable, with a likely role of induced systemic resistance in suppressiveness. The biogeography of suppressive soils has been neglected so far, but this works highlights the importance of reconsidering suppressiveness in this framework.

## Methods

### Soils

The experiment was conducted with four Swiss soils (MS16, MS7, MC112, MC10) [6, 8, 59, 60] and four Savoie soils (Asa2, Amo1, Ysa5, Ymo4) [5, 14] (Additional file 1: Fig. S11). The Swiss soils (located near Morens) originate from molasse sandstone bedrock and are conducive to black root rot (MC112 and MC10), or from morainic material and are suppressive to the disease (MS16 and MS7). In Savoie, Albens is at 130 km of Morens and Seyssel at 120 km, with 22 km between Albens and Seyssel. Two of the Savoie soils were formed on sandstone (Asa2 near Albens; Ysa5 near Seyssel) and the two others on morainic deposits (Amo1 near Albens; Ymo4 near Seyssel). Within the Morens, Albens and Seyssel areas, the different soils are distant of 2–5 km from each other.

In each of the eight fields, soil samples were taken from 10–30 cm depth at three locations (approximately 5–10 m apart) and were mixed (about 10 kg per field). Root residues and stones were removed, and the soils were sieved (6 mm). Soil was collected in November 2021 and stored in a cold room before starting the pot experiment in January 2022. Soil physicochemical analyses were conducted by the Fruit Research Institute (Čačak, Serbia), based on standard soil analysis testing (Additional file 2: Table S13).

### Soil organic matter profiling

Soil organic matter profiling was done after the soils were lyophilized for 48 h (Alpha 1–4 LSC, Bioblock Scientific, Illkirch, France), ground using a FastPrep-24 5G machine (MP Biomedicals, Mumbai, India) and sifted with a 200 mm × 50 mm riddle (ISO 3310–1, 250 µm, Fisher Scientific, Hampton, NH, USA). A modified Bligh-Dyer 2-phase extraction [61] was used for soil metabolites. Each soil sample (5 g) was treated with 4 mL of 0.25 M ammonium bicarbonate, 10 mL of methanol and 5 mL of CHCl_3_ in 50-mL centrifuge tubes with Teflon-lined screw cap. The mix was sonicated (50–60 Hz, 550 W, FisherBrand FB15061) for 10 min and incubated 2 h with end-over-end mixing, at room temperature. After shaking, samples were centrifuged for 10 min at 1710 g (Centrifuge 5804; Eppendorf SE, Hamburg, Germany) and the solution was transferred into a new tube, which received 5 mL of H_2_O and 5 mL of CHCl_3_. Another sonication (10 min) and centrifugation (at 1710 g, 10 min) were performed, which gave an aqueous phase (top layer; discarded) and an organic phase (bottom layer; studied further). The organic phase was transferred into a conical flask and concentrated using a Rotavapor (Büchi, Flawil, Switzerland), and a CentriVap concentrator (Labconco Corporation, Kansas City, MO, USA). A specific volume of methanol was added to each dry extract, depending on their mass, to reach a final concentration of 10 mg/mL. Samples were centrifuged (1710 g, 10 min) and 100-µL aliquots were transferred into vials for analysis with an Agilent 7890 A GC System with an Agilent 7000 A GC/MS Triple Quad (Agilent Technologies, Santa Clara, CA, USA). Samples (2 µL) were introduced by spitless injection. A DB-5 ms GC analytic column (Agilent Technologies), with 60 m long × 250 µm internal diameter × 250 µm film thickness, was used. Temperatures were as follows: inlet 290ºC, oven 100ºC for 3 min, 8ºC per min to 300ºC, hold at 300ºC for 17 min, source temperature 230ºC. Data were analysed with W4M Galaxy [62] and then compared to NIST Research Library (https://www.nist.gov/nist-research-library) to annotate metabolites.

### Experimental set-up

Half of the plants were inoculated with *Thielaviopsis basicola* to apply a strong disease pressure. *T. basicola* Ferraris strain ETH D127 was grown for three weeks on malt agar medium at 20–22 °C, in the dark, as described [6]. Spores (endoconida) were recovered by scraping the surface and the spore suspension was filtered using sterile Miracloth (Merck, Lyon, France). The spore concentration (estimated with a Thoma cell) was adjusted to 10^6^ spores/mL.

Seeds of *Nicotiana glutinosa* L. were pregerminated. Prior to transfer into soil, seedlings were grown for 4 weeks in sterile vermiculite containing Knop’s solution [15], incubated in a plant growth chamber at 70% relative humidity with 16 h day (880 μmol/m^2^/s) at 22 °C and 8 h night at 18 °C. For each soil, 10 control pots and 10 inoculated pots (8 × 8 × 8 cm), each containing 510 cm^3^ of soil (about 300 g dry soil), were used (i.e. 10 replicates). Each pot received one tobacco seedling and soil surface was inoculated with 5 mL of *T. basicola* suspension (or received 5 mL of sterile water in the non-inoculated control). The pots were placed in a growth chamber for three weeks (16 h day at 22 °C, 8 h night at 18 °C, 70% relative humidity, 880 µmol/m^2^/s), with a randomized block design (10 blocks). The plants were watered every two days with distilled water, to reach 70% of water retention capacity.

### Harvest, measurements and sampling

At harvest, the assessment included (i) the number of plants alive, (ii) the presence of necrosis on roots (black root rot disease severity), (iii) root biomass, and (iv) shoot biomass. Disease severity was assessed for each plant, based on the percentage of the root surface affected by necrosis (and where chlamydospores of *T. basicola* were produced). To this end, root disease was scored visually with a previously-described eight-class symptom scale [7], using midpoints of disease severity intervals. Symptom data for Swiss soils have already been published [15].

### Metabolomic analysis of tobacco

Tobacco shoots were freeze-dried using an Alpha 1–4 LSC (Martin Christ, Osterode am Harz, Germany) and then ground (Tissue Lyser II; Qiagen, Hilden, Germany). The powder obtained was extracted twice with methanol at a rate of 1.5 mL per 100 mg. Extracts were dried and dissolved at 10 g/L in methanol. Two µL of each extract were analyzed on an Accurate-Mass Q-TOF 6546 instrument coupled with an LC 1290 Infinity II system (Agilent Technologies). The separation was carried out at 40 °C using a 120 EC-C18 column (3.0 × 100 mm × 2.7 μm; Agilent Poroshell) with water, with 0.4% formic acid (v/v) as eluent A and acetonitrile as eluent B. The elution gradient (0.7 mL/min) was run with eluent B from 1 to 57% in 8.5 min, then 93% in 7 min and finally a plateau at 100% from 16.5 to 18.5 min. Mass analyses were made in positive mode with the nebulization gas (nitrogen) at a flow of 12 L/min and 40 PSG pressure, and with a capillary tension of 3500 V. All chromatograms were explored with Mass Hunter Qualitative Analysis B.07.00 software (Agilent Technologies) and data were processed with the Galaxy platform ‘Workflow4metabolomics’ [62].

### Rhizosphere DNA extraction

The rhizosphere was sampled at 3 weeks [63] from six inoculated plants and six non-inoculated plants for each of the eight soils. Loosely adhering soil was separated from roots by shaking and discarded. Roots and their tightly-adhering rhizosphere soil were placed in liquid nitrogen, lyophilized during 48 h and stored at −20 °C. Root-adhering soil was mechanically separated (using sterile tweezers) and 0.3–0.5 g of soil was used to extract DNA, using the FastDNA SPIN kit for Soil and a FastPrep-24 machine (MP Biomedicals, Santa Ana, CA, USA), following manufacturer’s instructions. DNA was eluted in 80 µL of DNase-free water and assessed using a Qubit dsDNA High Sensitivity Assay Kit with an Invitrogen Qubit 4.0 Fluorometer (Thermo Fisher Scientific), as well as a UV spectrophotometer (NanoPhotometer NP80, Implen GmbH, Munich, Germany).

### Sequencing of 16S rRNA gene and ITS region from rhizosphere DNA

The amplicon libraries targeting the V3-V4 region of the 16S rRNA gene were prepared with the primers Uni341F/Uni806R [64–66], incorporating Illumina adaptors (Nextera XT Index Kit; Illumina, San Diego, CA, USA). Library preparation and sequencing were performed at Novogene (UK) using the Illumina MiSeq platform with v2 (2 × 250 bp) chemistry, following the manufacturer’s protocol.

The ITS2 region of fungi was amplified with primers fITS7/ITS4 [67, 68] carrying the same Illumina adaptors. For high-fidelity amplification, PCR was done using Kapa Hifi HotStart ReadyMix (KAPA Biosystems, Wilmington, MA, USA). The PCR was done in triplicate in a S1000 Thermal Cycler (Bio-Rad), with an initial denaturation at 98 °C for 3 min, followed by 30 cycles of 98 °C for 20 s, 56 °C for 20 s, 72 °C for 20 s and a final elongation step at 72 °C for 5 min. The purification of PCR products was achieved using AMPure XP beads. An index PCR was performed using the Illumina Nextera XT Index Kit and Kapa Hifi HotStart ReadyMix (KAPA Biosystems) to assign the sequences to the respective samples, according to the manufacturer’s instructions. PCR products were again purified with AMPure XP beads, libraries were pooled equimolarly, and the pools were checked with an Agilent 2100 Bioanalyzer (Agilent Technologies, Palo Alto, CA, USA). The libraries were quantified with Qubit dsDNA-HS Assay (Invitrogen), following manufacturer's instructions, and paired-end Illumina MiSeq sequencing (2 × 300 bp) was performed at UFZ-Helmholtz Centre for Environmental Research.

### Processing of metabarcoding sequence data

16S rRNA gene sequences were treated using the R package Divisive Amplicon Denoising Algorithm (DADA2) v.1.12.1 [69]. Quality filtering and trimming were carried out with the ‘FilterAndTrimmed’ function and reads shorter than 100 bp were discarded, allowing two errors per read. ITS sequences were treated with dadasnake v.10 (https://github.com/a-h-b/dadasnake) [70], which is based on DADA2. Only reads with the expected PCR primers were retained, and primer sequences were removed using cutadapt v.1.18 [71]. Fungal reads with expected error rates (maxEE) lower than 3 and a minimum base quality of 9 were further considered. For both datasets, read pairs were merged with zero mismatches and exact sequence variants were identified as Amplicon Sequence Variants (ASVs). Chimeric sequences were removed using the DADA2 'consensus' algorithm. The ASVs based on the 16S rRNA gene and ITS2 sequences were taxonomically characterized using the SILVA database v.138 [72] and the UNITE database v.9 [73], respectively, based on the mothur [74] implementation of the Bayesian Classifier. ASVs that were unclassified or corresponded to chloroplasts, mitochondria, or eukaryotes were excluded from the 16S rRNA gene dataset. The phylum nomenclature was updated to the current taxonomic classifications [75]. For ITS data, all ASVs assigned to fungi were kept. Both datasets were normalized to an even sequencing depth through rarefaction, matching the lowest number of sequences observed across all samples [76]. Rarefaction curves for both datasets approached a plateau, indicating that microbial diversity was comprehensively captured. An additional column was added to provide the lowest taxonomic information available for each ASV in metabarcoding tables of the GitLab repository.

### Metagenomic analysis

Whole-community rhizosphere DNA was sequenced by Genewiz (Leipzig, Germany) using a NovaSeq 6000, resulting in 35 to 50 million paired-end reads (2 × 150 bp) per sample. For all metagenomes, Illumina TruSeq adapter sequences (forward: AGATCGGAAGAGCACACGTCTGAACTCCAGTCA; reverse: AGATCGGAAGAGCGTCGTGTAGGGAAAGAGTGT) were removed from reads with cutadapt v.4.6, and low-quality reads were filtered out using Trimmomatic v.0.39 [77] with the following options: ‘PE LEADING:3 TRAILING:3 SLIDINGWINDOW:4:15 MINLEN:30’. The reads were then mapped to the tobacco reference genome (*Nicotiana tabacum*; genome assembly Ntab-TN90) with Bowtie2 v.1.3.1 [78], and only pairs of unmapped reads were kept, using samtools view v.1.11 [79] with options -b -f 12 -F 256. The SqueezeMeta pipeline [80] (v1.6.3, September 2023) was used for the following analysis steps. The rhizosphere metagenome reads were coassembled (regardless of the treatment) using MEGAHIT v.1.2.9 [81]. Short contigs (< 200 bp) were removed and contigs statistics computed with prinseq v.0.20.4 [82] (Additional file 2: Table S14). We used Prodigal v.2.6.3 [83] to predict ORFs, respectively, and functional annotation was performed with GenBank [84] and EggNOG [85] databases using Diamond blastp v.0.8.28 [86] for similarity searches. In addition, HMMER3 v.3.2.1 [87] was used to search predicted sequences against the Pfam database [88]. Reads were then mapped against contigs with Bowtie2 [78] and read counts were normalized based on gene length and metagenome sequencing depth, to obtain “transcripts” per million (TPMs) values.

To have an accurate vision of taxonomic differences between samples, we averaged TPMs for a set of 15 single-copy genes (SCGs), identified with 20 Pfam domains associated to ribosomal proteins [20]. Genomic bins were reconstructed with Metabat2 v.2.12.1 [89] and CONCOCT v.1.1.0 [90], and redundant bins were dereplicated and aggregated with DAS Tool v.1.1 [91]. The resulting bin statistics (completeness and contamination levels) were computed using CheckM v1.2.2 [92] (Additional file 2: Table S14). Bin sequences with > 50% completeness and < 10% contamination (meeting the criteria for medium-quality drafts [93], i.e. MAGs) were then annotated using antiSMASH v.7.0 [94] for bacteria and archaea (no fungi). Finally, we compared bins with < 5% contamination to close type strains using the Type Strain Genome Server (TYGS) [95, 96].

### Statistics

#### Statistical approach

Unless otherwise stated, all analyses (*P* < 0.05) were conducted in R v.4.4.0 and figures plotted with R package ggplot2 v.3.5.1 [97]. The statistical models considered the effects of ‘Soil’ (individual soils), ‘Inoculation’ (with/without *T. basicola* inoculation), ‘Geography’ (Switzerland or Savoie), ‘Geology’ (sandstone or moraine origin) and ‘Status’ (suppressive or conducive). Sample ordination was assessed by PCA and PLS-DA, both using R package mixOmics v.6.28.0 [98], and/or NMDS with the metaMDS function implemented in R package vegan v.2.6.8 [99]. PERMANOVA were conducted with R package vegan (9999 permutations [thus documenting probability levels down to 1/9999 = 1 × 10^–4^], Bray–Curtis dissimilarities), using the ‘adonis2’ function. We also used the R packages stats v.4.4.0 for Student *t*-tests (‘t.test’ function), Wilcoxon rank sum tests (‘wilcox.test’) and correlation tests (‘cor.test’), rstatix v.0.7.2 for Kruskal–Wallis tests (‘kruskal_test’), and vegan for Mantel tests (‘mantel’ and ‘mantel.partial’).

#### Statistics for soil physicochemical data and soil metabolome data

The data from standard soil physicochemical analyses were analysed using PCA and PLS-DA supervised by ‘Status’ (i.e. suppressiveness status). PERMANOVA were conducted with ‘Y = Status’ and ‘Y = Geography × Geology’. Correlations with disease severity variables, i.e. (i) average symptom level in the control (Dc) or the pathogen inoculation treatment (D_Tb_), and (ii) relative impact of pathogen inoculation ((D_Tb_ + D_C_)/D_C_) were computed. Each variable was also compared in suppressive vs conducive soils using Student’s *t*-tests.

For soil metabolome data, we conducted PERMANOVAs with the models ‘Y = (Geography × Geology)/Soil’ and ‘Y = Geography × Status’. We then performed a PCA and a PLS-DA supervised by ‘Soil’ to identify ordination trends between soil metabolomes.

#### Statistics for plant data

Differences in disease severity data and in plant biomass data were assessed using Wilcoxon rank sum tests (*P* < 0.05) and correlation tests (Spearman, *P* < 0.05).

Shoot metabolome data were analysed by PERMANOVA using the model ‘Y = (Geography × Geology)/Soil × Inoculation’. To identify patterns in sample ordination, we performed PLS-DAs supervised by ‘Soil’ and ‘Inoculation’. Pairwise Bray–Curtis dissimilarities between suppressive and conducive soils were computed separately for Savoie and Switzerland, and statistical appraisal was carried out by comparing to a null distribution of differences obtained by randomly permuting geographic labels 99,999 times.

#### Statistics for metabarcoding data

Samples with low number of reads (< 20,000 reads) were discarded. Alpha diversity was computed, and sequences were rarefied based on the lowest number of sequences identified among samples, with a minimum of 22,182 16S rRNA gene sequences and 38,554 ITS sequences. Alpha diversity indices were computed for each rarefied sample using the vegan R package [99]. Kruskal–Wallis tests were used, and when this led to rejecting the null hypothesis (*P* < 0.05), LSD tests with Bonferroni correction were performed to compare treatments, using the agricolae R package v.1.3.7 [100]. Wilcoxon rank sum tests (*P* < 0.05) were used to compare diversity indices between inoculated and non-inoculated conditions for each soil, with the ‘compare_means’ function in ggpubr v.0.6.0 R package [101]. The diversity indices were also treated with an ANOVA with permutation test (9,999 permutations), to compare groups using a ‘Y = Soil × Inoculation’ model.

Beta diversity was assessed using the rarefied datasets and Bray–Curtis distances. Data were treated by PERMANOVA and NMDS to compare soils and assess the inoculation effect. The relationship between geographic distance and community dissimilarity was evaluated with simple and partial (i.e. corrected for physicochemical dissimilarity between soils) Mantel tests (*P* < 0.05). Indicator taxa of soil suppressiveness (at genus level) were obtained using the function ‘multipatt’ of the indicspecies v.1.7.15 R package [102]. Indicators with *P* < 0.05 and IndVal > 0.5 were kept.

#### Statistics for metagenomics data

To identify variations in metagenome taxonomic composition, the total SCG TPMs were averaged by phylum and Wilcoxon tests (FDR correction) were used on the relative SCG TPM count (i) by phylum for every phylum that represented > 1% of the TPM counts, and (ii) by class (> 0.1% of TPM counts) – we considered that abundance differences between lower counts could not be distinguished from noise – to compare between (i) Savoie soils, (ii) Swiss soils, and (iii) suppressive soils in Savoie vs Switzerland.

For ordination of the functional potential of rhizosphere communities, we calculated relative abundances for clusters of orthologous genes (COGs) identified in the metagenomes and computed an NMDS, and identified factors (geology, suppressive status, inoculation) with a significant effect on community functions with a PERMANOVA, using both a ‘Geography × Status × Inoculation’ and a ‘Geography × Geology × Inoculation’ model. Simple and partial (i.e. corrected for physicochemical dissimilarity between soils) Mantel tests (*P* < 0.05) assessed the relationship between geographic distance and dissimilarity in functional potential. We then used a Cumulative Sum Scaling (CSS) normalization technique on read counts (implemented in the ‘cumNorm’ function in the metagenomeSeq v.1.46.0 R package)—which accounts for uneven sequencing depths [103]—and removed COGs present in less than half the effective samples. Next, we applied a Zero-Inflated Gaussian Distribution Mixture Model to these counts (‘fitZig’ function in metagenomeSeq) and performed differential abundance analysis (which returned both adjusted p-values and log_2_-transformed fold changes [or ‘logFC’] between soils) with the ‘eBayes’ function of the limma v.3.60.6 R package [104] to compare (i) suppressive vs conducive soils, separately for Savoie and for Switzerland, and (ii) Savoie vs Swiss soils, for suppressive soils only and for all soils. We also focused on COGs belonging to the Secondary metabolites biosynthesis, transport and catabolism (Q) COG main category and computed a heatmap using the ComplexHeatmap v.2.20.0 R package [105] with CSS-normalized counts and Wilcoxon tests (FDR correction) to identify COGs that were significantly more abundant in suppressive soils, separately for each region.

To identify microbial functional traits potentially associated to soil properties, we conducted correlations (Spearman) between (i) the physicochemical variables measured for the four soils investigated with metagenomics and (ii) the average CSS-normalized values of read counts of the main COG categories or the COGs of the Q category. The limited number of soils (*N* = 4) was not sufficient to compute probabilities, but a Spearman coefficient of 1 or −1 was chosen to point potential relations.

To measure MAG abundance (filtered for completeness and contamination) in each sample, the TPM count of their contigs was calculated, as some MAGs had ORFs that were not assigned to any of the Pfam domains of the previously used SCGs set. Additionally, average MAG TPM values were analyzed to identify those significantly more abundant in suppressive soils for (i) Savoie and (ii) Swiss soils. We then analyzed the functional profile of secondary metabolite biosynthesis, transport, and catabolism by summing TPM values for Q-category COGs assigned to ORFs in each MAG's contigs and generating a heatmap showing COG abundance and MAG abundance across samples (using ComplexHeatmap). TPM values were used to conduct FDR-corrected Wilcoxon tests and calculate ratios to identify COGs significantly more abundant in suppressive soils for (i) Savoie and (ii) Swiss soils. A similar analysis was performed using antiSMASH annotation results, where the number of protoclusters, grouped by type, was used to generate a heatmap alongside MAG abundance across samples.

## Supplementary Information

Additional file 1: Fig. S1. Performance of tobacco after 3 weeks of growth in soils from Savoie and Switzerland, in the presence or absence of added *Thielaviopsis basicola*. Fig. S2. Impact of *Thielaviopsis basicola* inoculation and soil status on the correlations between various metrics of tobacco performance at 3 weeks of growth in Savoie and Switzerland soils. Fig. S3. Physicochemistry of the eight soils used for the plant experiment. Fig. S4. Fungal indicator taxa for suppressiveness depending on geographic region and pathogen inoculation. Fig. S5. Prokaryotic indicator taxa for suppressiveness depending on geographic region and pathogen inoculation. Fig. S6. Correlation matrices between soil physicochemical characteristics and the average CSS-normalized read count of broad COG categories and COGs belonging to the Secondary metabolites biosynthesis, transport and catabolism COG category. Fig. S7. Heatmap of Cumulative Sum Scaling-transformed read count of COG belonging to the Q category. Fig. S8. Taxonomic distribution and abundance of MAGs based on soil and inoculation conditions, with abundance measured as the TPM sum of contigs within each MAG. Fig. S9. Heatmaps displaying MAG abundance in the metagenome and individual Q-category COG abundance within those MAGs. Fig. S10. Tree inferred with FastME 2.1.6.1 from GBDP distances calculated from genome sequences of 26 type strains closely related to the *Pseudomonas* MAG. Fig. S11. Map of the regions of Morens and Savoie where soils were collected.

Additional file 2: Table S1. PERMANOVA results for the soil metabolome, with two models. Table S2. PERMANOVA results for the ITS metabarcoding, with three models. Table S3. PERMANOVA results for the 16S metabarcoding, with all soils and three models and separate soils for which the effect of pathogen inoculation was significant. Table S4. Comparison of most abundant phyla in the metagenomes of suppressive and conducive soils. Table S5. Comparison of most abundant classes in the metagenomes of suppressive and conducive soil samples. Table S6. PERMANOVA results for the abundance of COGs in the metagenome. Table S7. Comparison of the abundance of COG main categories between soils and number of differentially abundant COGs within the categories. Table S8. Comparison of the abundance and differential abundance analysis of individual COGs belonging to the ‘Q’ main COG category. Table S9. Details on the bins reconstructed with the coassembled tobacco rhizosphere metagenomes. Table S10. AntiSMASH annotation for bins that are > 50% complete and < 10% contaminated. Table S11. Subject strains used for the comparison with the *Pseudomonas* bin 1 with dDDH values and G + C difference when compared to Bin 1. Table S12. PERMANOVA results for the metabolome of tobacco shoots. Table S13. Main physicochemical characteristics of Swiss and Savoie soils. Table S14. Quality control for the shotgun metagenome assembly and binning.

## Data Availability

Some of the data for 4 of the 8 soils in Fig. 1 have been extracted from Harmsen et al. [15]. The raw genomic data used in this study are available on the NCBI SRA database under BioProject accession number PRJNA1224598 (https://www.ncbi.nlm.nih.gov/sra?linkname=bioproject_sra_all&from_uid=1224598), which comprises raw reads from metagenomic sequencing of the tobacco rhizosphere and raw reads from 16S and ITS amplicon sequencing of the tobacco rhizosphere. These correspond to 204 SRA links, i.e. accessions SRX28092898 to SRX28109212 (https://www.ncbi.nlm.nih.gov/sra?linkname=bioproject_sra_all&from_uid=1224598) [108]. Processed data (in the form of tables) is available on a GitLab hosted at the IN2P3 center in Université Claude Bernard Lyon 1 (https://gitlab.in2p3.fr/alix.catry/sps_tobacco/) [106]. Scripts for statistical analysis and generating figures are available on a GitLab hosted at the IN2P3 center in Université Claude Bernard Lyon 1 (https://gitlab.in2p3.fr/alix.catry/sps_tobacco/-/tree/main/Scripts) [106]. Both scripts and processed data are also available in a Zenodo repository [107].
