# Supplementary material for: Biogeography influences plant–microbe interactions and natural soil suppressiveness to black root rot disease of tobacco
Source: Genome Biol. 2025 Dec 28;27:16. doi: 10.1186/s13059-025-03911-0 (PMC12857086; doi:10.1186/s13059-025-03911-0)
Supplement: Supplementary file 1 — Additional file 1: Fig. S1. Performance of tobacco after 3 weeks of growth in soils from Savoie and Switzerland, in the presence or absence of added Thielaviopsis basicola. Fig. S2. Impact of Thielaviopsis basicola inoculation and soil status on the correlations between various metrics of tobacco performance at 3 weeks of growth in Savoie and Switzerland soils. Fig. S3. Physicochemistry of the eight soils used for the plant experiment. Fig. S4. Fungal indicator taxa for suppressiveness depending on geographic region and pathogen inoculation. Fig. S5. Prokaryotic indicator taxa for suppressiveness depending on geographic region and pathogen inoculation. Fig. S6. Correlation matrices between soil physicochemical characteristics and the average CSS-normalized read count of broad COG categories and COGs belonging to the Secondary metabolites biosynthesis, transport and catabolism COG category. Fig. S7. Heatmap of Cumulative Sum Scaling-transformed read count of COG belonging to the Q category. Fig. S8. Taxonomic distribution and abundance of MAGs based on soil and inoculation conditions, with abundance measured as the TPM sum of contigs within each MAG. Fig. S9. Heatmaps displaying MAG abundance in the metagenome and individual Q-category COG abundance within those MAGs. Fig. S10. Tree inferred with FastME 2.1.6.1 from GBDP distances calculated from genome sequences of 26 type strains closely related to the Pseudomonas MAG. Fig. S11. Map of the regions of Morens and Savoie where soils were collected. [file 13059_2025_3911_MOESM1_ESM.pdf]

**Additional file 1**

# Biogeography influences plant-microbe interactions and natural soil suppressiveness to black root rot disease of tobacco

Alix Catry[1], Danis Abrouk[1], Nicolas Fierling[1], Ana Isabel Serrano Mendoza[1], Marjolaine Rey[1], Pilar Vesga[2], Clara M. Heiman[2], Daniel Garrido-Sanz[2], Marie-Lara Bouffaud[3], François Buscot[3], Adriana Giongo[4], Kornelia Smalla[4], Gilles Comte[1], Christoph Keel[2], Daniel Muller[1] and Yvan Moënne-Loccoz[1,5*]

[1]Université Claude Bernard Lyon 1, CNRS, INRAE, VetAgro Sup, UMR5557 Ecologie Microbienne, 43 bd du 11 novembre 1918, F-69622 Villeurbanne, France

[2]University of Lausanne, Department of Fundamental Microbiology, Quartier UNIL-Sorge, CH-1015 Lausanne, Switzerland

[3]Department of Soil Ecology, Helmholtz Centre for Environmental Research - UFZ, Theodor-Lieser-Str. 4, D-06120 Halle/Saale, Germany

[4] Institute for Epidemiology and Pathogen Diagnostics, Julius Kühn Institute (JKI) – Federal Research Centre for Cultivated Plants, Messeweg 11-12, D-38104 Braunschweig, Germany

[5]Institut Universitaire de France (IUF), F-75005 Paris, France

## Supplementary figures

**Fig. S1.** Performance of tobacco after 3 weeks of growth in soils from Savoie and Switzerland, in the presence or absence of added *Thielaviopsis basicola*. (A) Fresh root biomass, (B) Dry root biomass, (C) Fresh shoot biomass, (D) Number of green leaves, (E) Number of green and damaged leaves, and (F) Mortality. In all graphs, the uninoculated controls are represented by empty circles and bars and inoculated treatment by filled ones (n = 10 individual plants per condition). For each soil, significant differences (Wilcoxon tests in A-E, Chi-square tests in F) between inoculated and control treatments are indicated by * ($P < 0.05$), ** ($P < 0.01$) or *** ($P < 0.001$). NS, not significant. For each soil, the status ('Cond' for conducive, 'Supp' for suppressive) and sampling region ('Savoie' or 'Switzerland') are indicated.

**Fig. S2.** Impact of *Thielaviopsis basicola* inoculation and soil status on the correlations between various metrics of tobacco performance at 3 weeks of growth in Savoie and Switzerland soils. On the upper right of the graph, correlation coefficients (Spearman's ρ) describe the relationship between each pair of metrics, as computed by R's 'cor.text()' (pairwise Spearman correlation) for (i) all samples, (ii) only those inoculated with *T. basicola*, (iii) only non-inoculated controls. Significant correlations are indicated by * ($P_{Spearman} < 0.05$), ** ($P_{Spearman} < 0.01$) or *** ($P_{Spearman} < 0.001$). NS, not significant. Scatter plots on the lower left of the graph represent the correlation between two variables in all soils; each point is an individual replicate (n = 10 individual plants per soil and treatment) with non-inoculated controls represented by empty circles and inoculated samples by filled samples. The regression line for the non-inoculated controls only is in grey and that for the inoculated samples only is in black. The density plots on the panel diagonal represent the distribution of the metrics depending on treatment; density curves are filled in white for non-inoculated controls and grey for inoculated samples. The far-right plot column represents the distribution of the metrics depending on the treatment (unfilled circles and boxplots for controls, filled ones for inoculated samples) and status of soil where tobacco plants were grown (suppressive soils in blue, conducive soils in red).

**Fig. S3.** Physicochemistry of the eight soils used for the plant experiment, based on (A,B) Principal Component Analysis (PCA) and (C,D) Partial Least Square-Discriminant Analysis (PLS-DA) supervised by soil status, using 11 physicochemical metrics. In A and B, the 'factoMineR' package on R was used for PCA, and PCA axes 1 and 2 are represented in A and PCA axes 1 and 3 in B (85% of the total variability). Points represent the individual soils, black arrows the variables, and red arrows supplementary quantitative variables, i.e. variables that were not used to construct the PCA but correlated *a posteriori* with the others ('Symptoms_inoc' and 'Symptoms_control' represent the symptom levels in samples inoculated with *T. basicola* and controls, respectively. 'Inoc_impact' represents the impact of the inoculation, calculated as $(D_{Tb} – D_C)/D_C$, with $D_{Tb}$ the disease severity of plants inoculated with *T. basicola* and $D_C$ that of non-inoculated plants). In C, points in the PLS-DA plot supervised by soil status (components 1 and 2) represent the individual soils and black arrows the

variables. In D (loadings plot on the first two axes of the PLS-DA), the length of the bar represents the weight of the contribution of the original variable to the axis. Bars are colored in blue when the mean of the variable's value was higher in suppressive soils, in red when it was higher in conducive ones. Significant differences (Student's *t*-tests, $P < 0.05$) between suppressive and conducive soils are indicated with *. CEC, Cation Exchange Capacity; SAB, CEC saturation with adsorbed bases (see Supplementary Table 1). For each soil, the status ('Cond' for conducive, 'Supp' for suppressive) and sampling region ('Savoie' or 'Switzerland') are indicated.

**Fig. S4.** Fungal indicator taxa for suppressiveness depending on geographic region and pathogen inoculation. Indicator taxa were identified following the indicator value approach of De Cáceres & Legendre (2009), and statistical significance was evaluated using permutation tests of group affiliation by using soil suppressive status as the group. On the upper X axis are indicated the name of the soils and on the Y axis the taxa. Taxa that correspond to family names (ending in -*ceae*) denote an unclassified genus belonging to said family. The size of the dot represents the mean relative abundance of the taxa in the rhizosphere of tobacco plants. Taxa are colored according to phylum. Indicator taxa were identified (A) in Savoie soils (Amo1, Ymo4, Asa2, Ysa5), without *T. basicola* inoculation; (B) in Savoie soils, with *T. basicola* inoculation; (C) in Swiss soils (MC10, MC112, MS16, MS7), without *T. basicola* inoculation; and (D) in Swiss soils, with *T. basicola* inoculation.
De Cáceres M, Legendre P. Associations between species and groups of sites: indices and statistical inference. Ecology 2009;90:3566–74.

**Fig. S5.** Prokaryotic indicator taxa for suppressiveness depending on geographic region and pathogen inoculation. Indicator taxa were identified following the indicator value approach of De Cáceres & Legendre (2009), and statistical significance was evaluated using permutation tests of group affiliation by using soil suppressive status as the group. On the upper X axis are indicated the name of the soils and on the Y axis the taxa. Taxa that correspond to family names (ending in -*ceae*) denote an unclassified genus belonging to said family. The size of the dot represents the mean relative abundance of the taxa in the rhizosphere of tobacco plants. Taxa are colored according to phylum. Indicator taxa were identified (A) in Savoie soils (Amo1, Ymo4, Asa2, Ysa5), without *T. basicola* inoculation; (B) in Savoie soils, with *T. basicola* inoculation; (C) in Swiss soils (MC10, MC112, MS16, MS7), without *T. basicola* inoculation; and (D) in Swiss soils, with *T. basicola* inoculation.
De Cáceres M, Legendre P. Associations between species and groups of sites: indices and statistical inference. Ecology 2009;90:3566–74.

**Fig. S6.** Correlation matrices between soil physicochemical characteristics and the average CSS-normalized read count of (A) broad COG categories and (B) COGs belonging to the Secondary metabolites biosynthesis, transport and catabolism (Q) COG category. SAB = CEC saturation. In A and

B, a Spearman coefficient of -1 or 1 between two traits is indicated by a star (*). In B, the gray squares underneath the heatmap indicate the COGs of the Q category that were significantly more abundant (Wilcoxon tests, $P$ < 0.05) in the rhizosphere of plants grown in suppressive vs conducive soils, in Switzerland (MS16 vs MC10) or in Savoie (Ysa5 vs Ymo4).

**Fig. S7.** Heatmap of Cumulative Sum Scaling (CSS)-transformed read count of COG belonging to the Q category. CSS counts are normalized by row with a min-max transformation, to highlight abundance differences between soils. The COG identifier is on the right of the heatmap and the COG function description on the left. Annotations on the top of the heatmap correspond to 'Geography' (soil sampling region) and 'Geology' (sandstone vs moraine). Annotations on the bottom of the heatmap refer to plant inoculation condition ('Tb' for *T. basicola*, 'C' for control), soil name, and soil status ('Supp' for suppressive, 'Cond' for conducive). Grey squares on the right of the heatmap highlight COGs that are significantly more abundant (Wilcoxon tests, $P$ < 0.05) in the suppressive soil than in the conducive soil, for (i) the Swiss soils (‘MS16 vs MC10’) and (ii) the Savoie soils (‘Ysa5 vs Ymo4’).

**Fig. S8.** Taxonomic distribution and abundance of MAGs based on soil and inoculation conditions, with abundance measured as the TPM sum of contigs within each MAG. Colors represent different taxonomic groups, with archaeal taxa shown in red and pink, and bacterial taxa in purple, blue, and green. Annotations below the barplot indicate the plant inoculation condition ('Tb' for *T. basicola* and 'C' for control), soil name, and soil status ('Supp' for suppressive and 'Cond' for conducive).

**Fig. S9.** Heatmaps displaying: (i) MAG abundance in the metagenome and (ii) individual Q-category COG abundance within those MAGs. MAG abundance is calculated as the average COG TPM per treatment (soil and inoculation), log-transformed to reduce bias from high TPM values. Annotations at the top of the heatmap indicate 'Geography' (soil sampling region) and 'Geology' (soil geological origin), while annotations at the bottom show plant inoculation condition ('Tb' for *T. basicola*, 'C' for control), soil name, and soil status ('Supp' for suppressive, 'Cond' for conducive). At the right of the MAG abundance heatmap is the lowest available taxonomic level for each MAG, along with bars representing MAG completeness ('Cp', as a percentage) and contamination ('Ct', as a percentage), both computed by CheckM. COG abundance values are averaged for each MAG, normalized by column using a min-max transformation to emphasize differences between MAGs, with gray squares indicating the absence of a COG in a MAG.

**Fig. S10.** Tree inferred with FastME 2.1.6.1 from GBDP distances calculated from genome sequences of 26 type strains closely related to the *Pseudomonas* MAG (‘Bin1’). The branch lengths are scaled in terms of GBDP distance formula $d_5$. The numbers above branches are GBDP pseudo-bootstrap support

values > 60 % from 100 replications, with an average branch support of 94.2 %. The analyses and the figure were computed through the TYGS bioinformatics platform.

**Fig. S11.** Map of the regions of Morens (Switzerland) and Savoie (France) where soils were collected. (A) Overview showing distances between Morens (Frame 1), and Seyssel and Albens in Savoie (Frame 2) (source: BRGM). (B) Soils MS16, MS7 (suppressive), MC112, MC10 (conducive) near Morens. Molasse sandstone areas are indicated in blue and moraine deposits in light yellow. Geology information was obtained from the Lithological map of Switzerland, using Groups of rocks at 1:500000 scale (Federal Office of Topography swisstopo, Geoservices of the Federal Spatial Data Infrastructure; https://map.geo.admin.ch/). (C) Soils Ysa5 (suppressive) and Ymo4 (conducive) near Seyssel, and soils Asa2 (suppressive) and Amo1 (conducive) near Albens (Savoie). Molasse sandstone areas are indicated in yellow and moraine deposits in light grey. Geology information in Savoie was derived from the geological map available at géoportail (https://www.geoportail.gouv.fr/carte), using data from the BRGM. GPS coordinates were 46.8849N and 6.9225E (MS16), 46.8614N and 6.8983E (MS7), 46.8378N and 6.8877E (MC112), 46.8634N and 6.9243E (MC10), 45.951028N and 5.852861E (Ysa5), 45.958139N and 5.857500E (Ymo4), 45.754694N and 5.982333E (Asa2), and 45.784639N and 5.998028E (Amo1).

Fig. S1

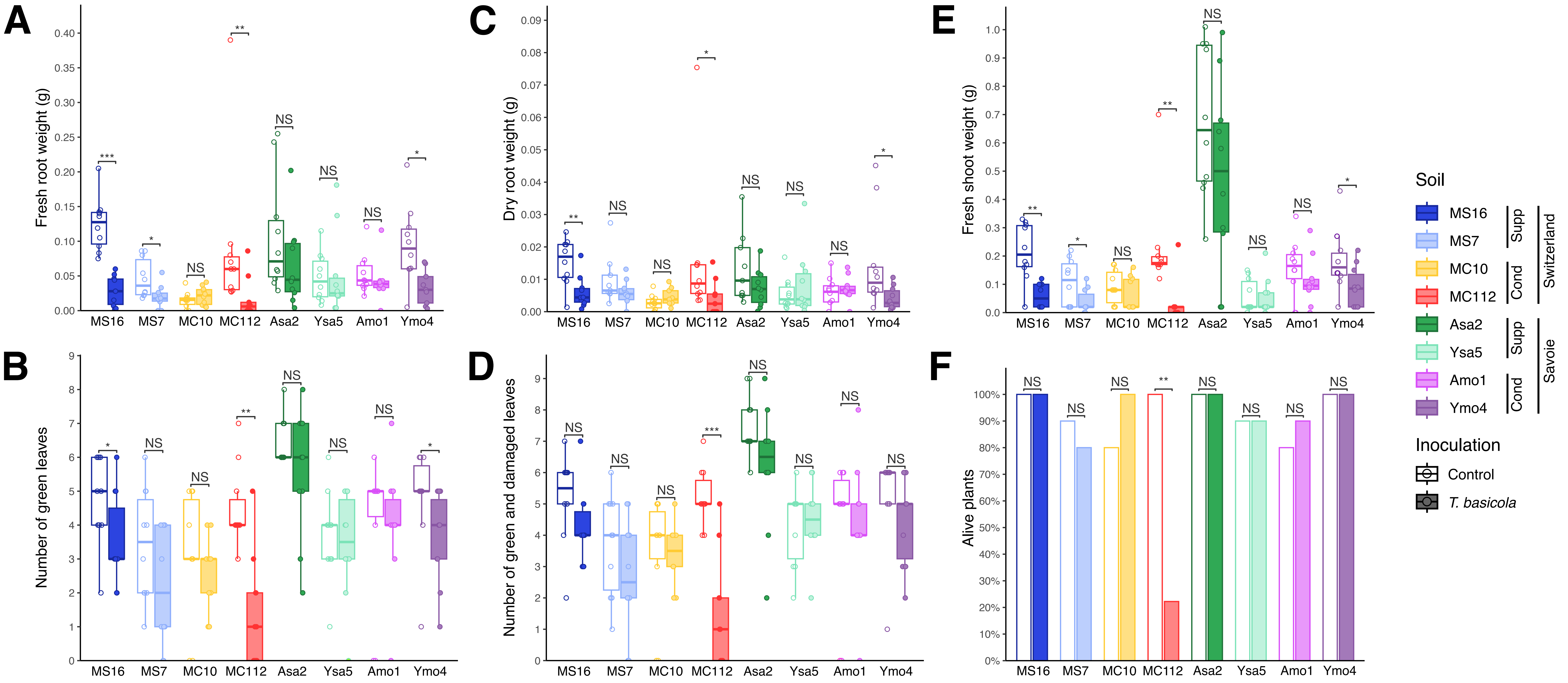

A
Fresh root weight (g)
B
Number of green leaves
C
Dry root weight (g)
D
Number of green and damaged leaves
E
Fresh shoot weight (g)
F
Alive plants
MS16
MS7
MC10
MC112
Asa2
Ysa5
Amo1
Ymo4
Soil
Supp
Cond
Switzerland
Savoie
Inoculation
Control
T. basicola

Fig. S2

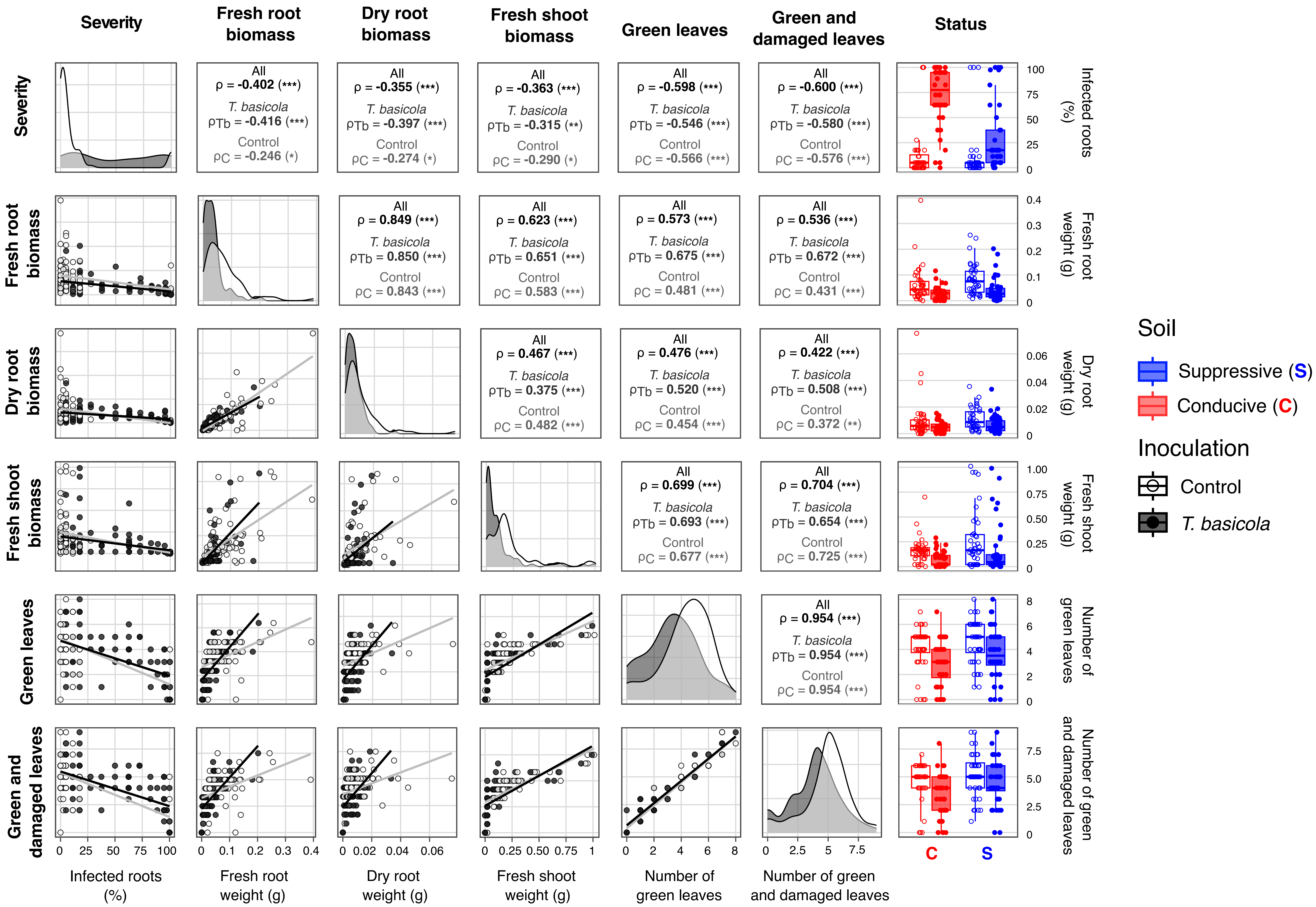

Severity
Fresh root biomass
Dry root biomass
Fresh shoot biomass
Green leaves
Green and damaged leaves
Status
All ρ = -0.402 (***) T. basicola ρTb = -0.416 (***) Control ρC = -0.246 (*)
All ρ = -0.355 (***) T. basicola ρTb = -0.397 (***) Control ρC = -0.274 (*)
All ρ = -0.363 (***) T. basicola ρTb = -0.315 (**) Control ρC = -0.290 (*)
All ρ = -0.598 (***) T. basicola ρTb = -0.546 (***) Control ρC = -0.566 (***)
All ρ = -0.600 (***) T. basicola ρTb = -0.580 (***) Control ρC = -0.576 (***)
Infected roots (%)
All ρ = 0.849 (***) T. basicola ρTb = 0.850 (***) Control ρC = 0.843 (***)
All ρ = 0.623 (***) T. basicola ρTb = 0.651 (***) Control ρC = 0.583 (***)
All ρ = 0.573 (***) T. basicola ρTb = 0.675 (***) Control ρC = 0.481 (***)
All ρ = 0.536 (***) T. basicola ρTb = 0.672 (***) Control ρC = 0.431 (***)
Fresh root weight (g)
All ρ = 0.467 (***) T. basicola ρTb = 0.375 (***) Control ρC = 0.482 (***)
All ρ = 0.476 (***) T. basicola ρTb = 0.520 (***) Control ρC = 0.454 (***)
All ρ = 0.422 (***) T. basicola ρTb = 0.508 (***) Control ρC = 0.372 (**)
Dry root weight (g)
All ρ = 0.699 (***) T. basicola ρTb = 0.693 (***) Control ρC = 0.677 (***)
All ρ = 0.704 (***) T. basicola ρTb = 0.654 (***) Control ρC = 0.725 (***)
Fresh shoot weight (g)
All ρ = 0.954 (***) T. basicola ρTb = 0.954 (***) Control ρC = 0.954 (***)
Number of green leaves
Number of green and damaged leaves
C
S
Infected roots (%)
Fresh root weight (g)
Dry root weight (g)
Fresh shoot weight (g)
Number of green leaves
Number of green and damaged leaves
Soil
Suppressive (S)
Conducive (C)
Inoculation
Control
T. basicola

Fig. S3

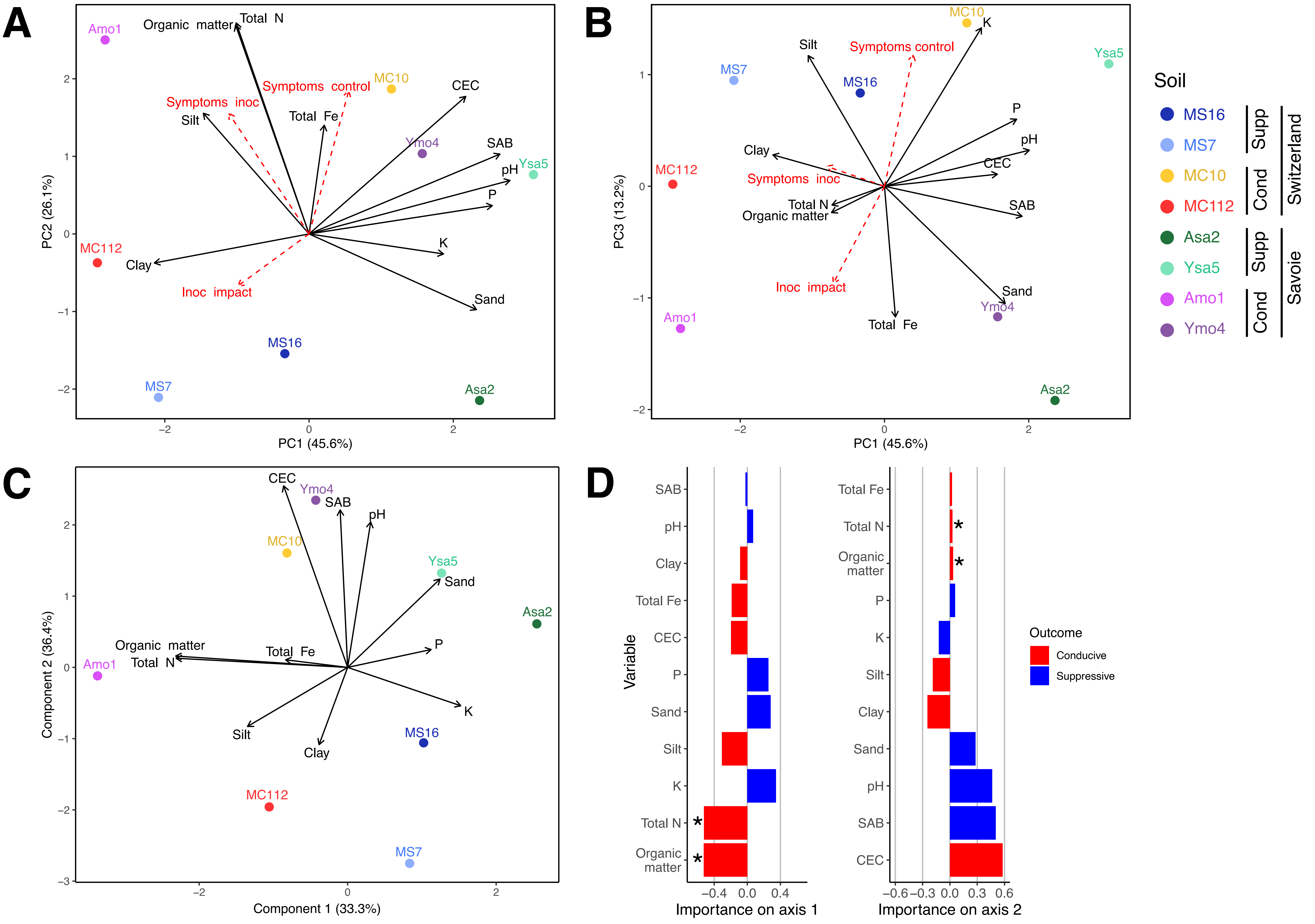
A
Amo1
Organic matter
Total N
Symptoms control
MC10
CEC
Symptoms inoc
Silt
Total Fe
Ymo4
SAB
pH
Ysa5
P
MC112
K
Clay
Inoc impact
Sand
MS16
MS7
Asa2
PC2 (26.1%)
PC1 (45.6%)
B
MC10
K
Silt
Symptoms control
Ysa5
MS7
MS16
P
pH
Clay
CEC
MC112
Symptoms inoc
Total N
SAB
Organic matter
Inoc impact
Sand
Ymo4
Amo1
Total Fe
Asa2
PC3 (13.2%)
PC1 (45.6%)
Soil
MS16
MS7
MC10
MC112
Asa2
Ysa5
Amo1
Ymo4
Supp
Cond
Switzerland
Supp
Cond
Savoie
C
CEC
Ymo4
SAB
pH
MC10
Ysa5
Sand
Asa2
Organic matter
P
Amo1
Total N
Total Fe
K
Silt
MS16
Clay
MC112
MS7
Component 2 (36.4%)
Component 1 (33.3%)
D
Variable
SAB
pH
Clay
Total Fe
CEC
P
Sand
Silt
K
Total N *
Organic matter *
Importance on axis 1
Total Fe
Total N *
Organic matter *
P
K
Silt
Clay
Sand
pH
SAB
CEC
Importance on axis 2
Outcome
Conducive
Suppressive

Fig. S4

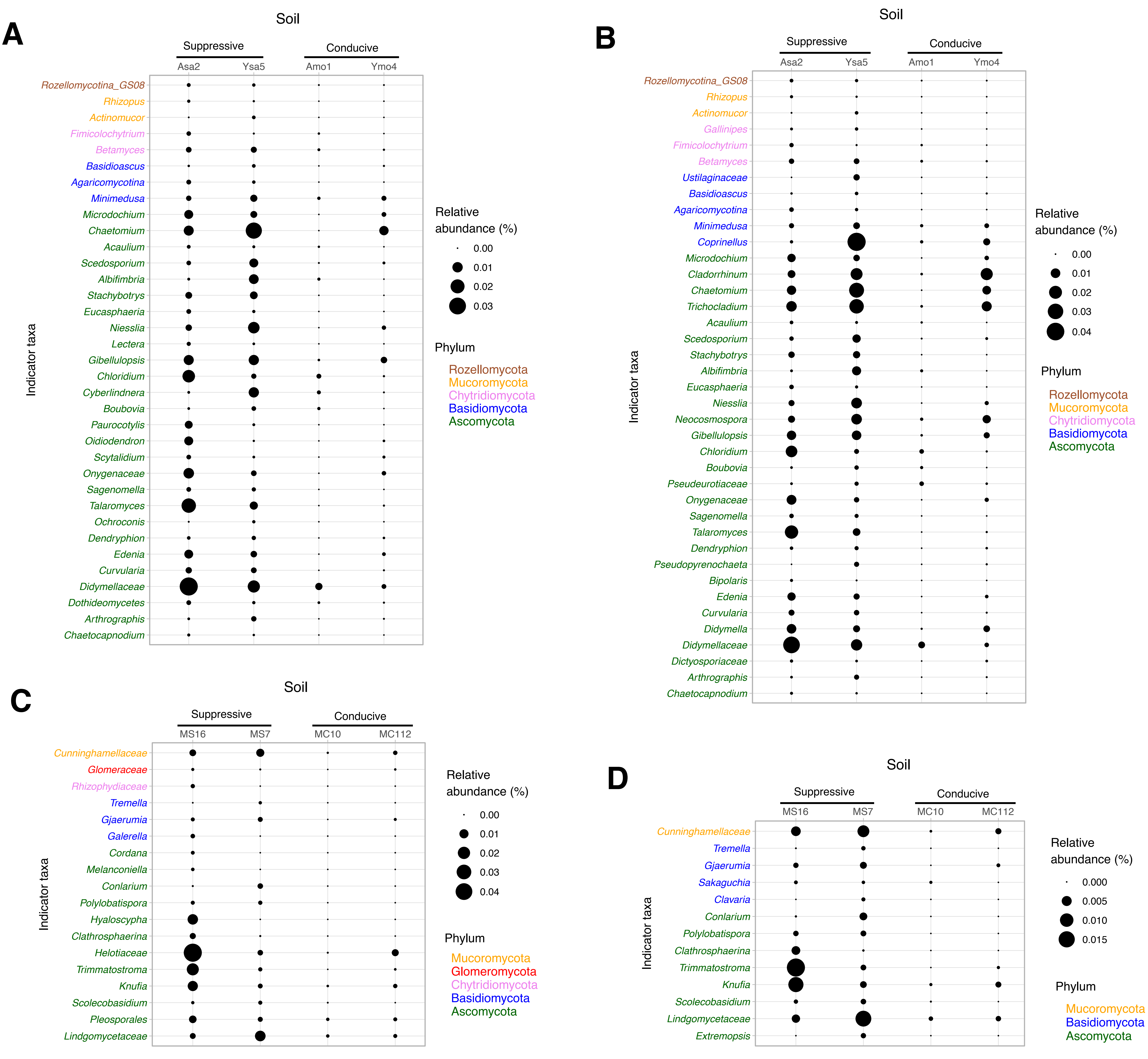

A
B
C
D
Soil
Suppressive
Conducive
Asa2
Ysa5
Amo1
Ymo4
MS16
MS7
MC10
MC112
Indicator taxa
Relative abundance (%)
Phylum
Rozellomycota
Mucoromycota
Glomeromycota
Chytridiomycota
Basidiomycota
Ascomycota
Rozellomycotina_GS08
Rhizopus
Actinomucor
Gallinipes
Fimicolochytrium
Betamyces
Ustilaginaceae
Basidioascus
Agaricomycotina
Minimedusa
Coprinellus
Microdochium
Cladorrhinum
Chaetomium
Trichocladium
Acaulium
Scedosporium
Albifimbria
Stachybotrys
Eucasphaeria
Niesslia
Neocosmospora
Lectera
Gibellulopsis
Chloridium
Cyberlindnera
Boubovia
Pseudeurotiaceae
Paurocotylis
Oidiodendron
Scytalidium
Onygenaceae
Sagenomella
Talaromyces
Ochroconis
Dendryphion
Pseudopyrenochaeta
Bipolaris
Edenia
Curvularia
Didymella
Didymellaceae
Dothideomycetes
Dictyosporiaceae
Arthrographis
Chaetocapnodium
Cunninghamellaceae
Glomeraceae
Rhizophydiaceae
Tremella
Gjaerumia
Sakaguchia
Galerella
Clavaria
Cordana
Melanconiella
Conlarium
Polylobatispora
Hyaloscypha
Clathrosphaerina
Helotiaceae
Trimmatostroma
Knufia
Scolecobasidium
Pleosporales
Lindgomycetaceae
Extremopsis
0.00
0.01
0.02
0.03
0.04
0.000
0.005
0.010
0.015

Fig. S5

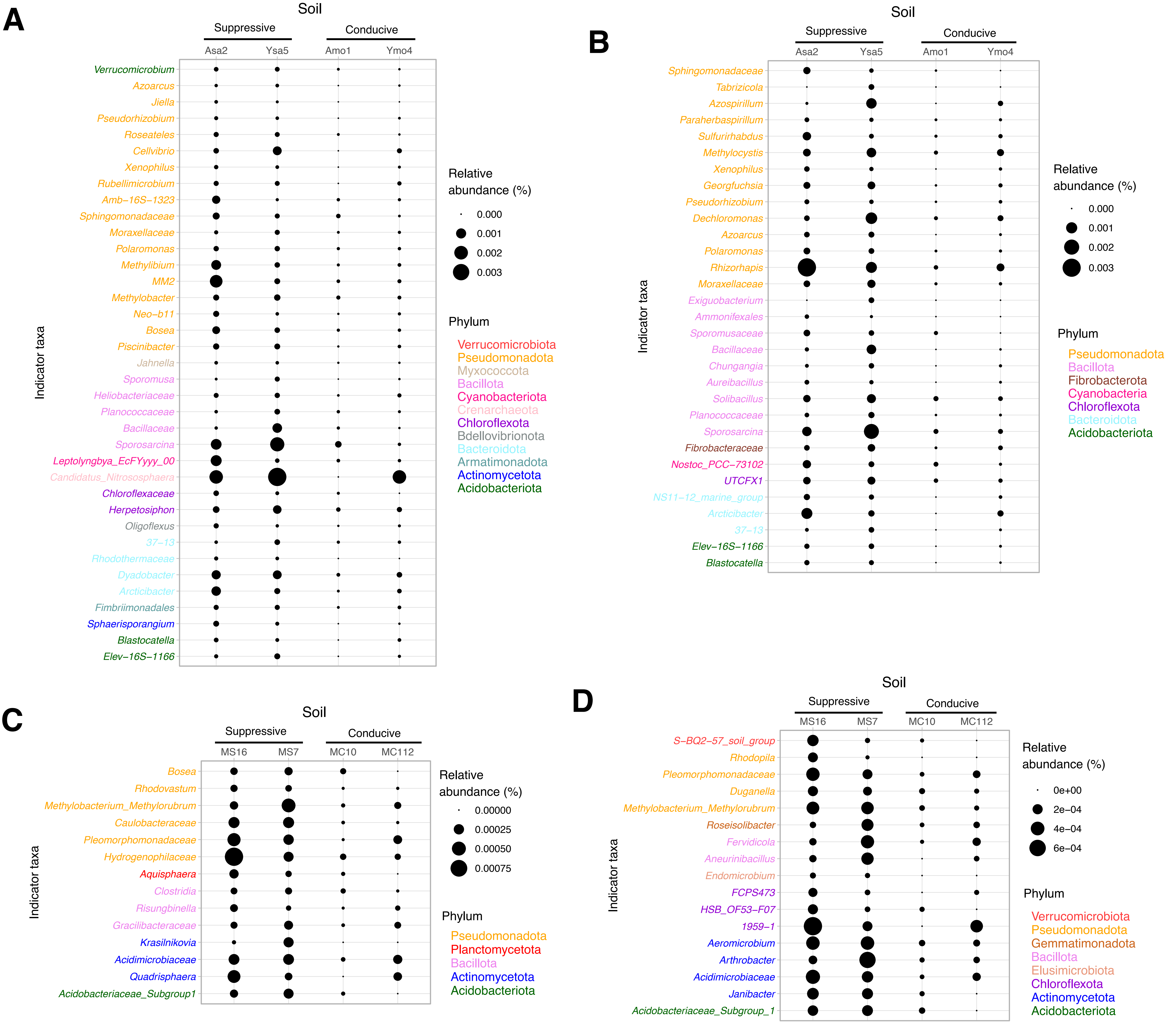
A
Soil
Suppressive
Conducive
Asa2
Ysa5
Amo1
Ymo4
Indicator taxa
Verrucomicrobium
Azoarcus
Jiella
Pseudorhizobium
Roseateles
Cellvibrio
Xenophilus
Rubellimicrobium
Amb−16S−1323
Sphingomonadaceae
Moraxellaceae
Polaromonas
Methylibium
MM2
Methylobacter
Neo−b11
Bosea
Piscinibacter
Jahnella
Sporomusa
Heliobacteriaceae
Planococcaceae
Bacillaceae
Sporosarcina
Leptolyngbya_EcFYyyy_00
Candidatus_Nitrososphaera
Chloroflexaceae
Herpetosiphon
Oligoflexus
37−13
Rhodothermaceae
Dyadobacter
Arcticibacter
Fimbriimonadales
Sphaerisporangium
Blastocatella
Elev−16S−1166
Relative abundance (%)
0.000
0.001
0.002
0.003
Phylum
Verrucomicrobiota
Pseudomonadota
Myxococcota
Bacillota
Cyanobacteriota
Crenarchaeota
Chloroflexota
Bdellovibrionota
Bacteroidota
Armatimonadota
Actinomycetota
Acidobacteriota
B
Soil
Suppressive
Conducive
Asa2
Ysa5
Amo1
Ymo4
Indicator taxa
Sphingomonadaceae
Tabrizicola
Azospirillum
Paraherbaspirillum
Sulfurirhabdus
Methylocystis
Xenophilus
Georgfuchsia
Pseudorhizobium
Dechloromonas
Azoarcus
Polaromonas
Rhizorhapis
Moraxellaceae
Exiguobacterium
Ammonifexales
Sporomusaceae
Bacillaceae
Chungangia
Aureibacillus
Solibacillus
Planococcaceae
Sporosarcina
Fibrobacteraceae
Nostoc_PCC−73102
UTCFX1
NS11−12_marine_group
Arcticibacter
37−13
Elev−16S−1166
Blastocatella
Relative abundance (%)
0.000
0.001
0.002
0.003
Phylum
Pseudomonadota
Bacillota
Fibrobacterota
Cyanobacteria
Chloroflexota
Bacteroidota
Acidobacteriota
C
Soil
Suppressive
Conducive
MS16
MS7
MC10
MC112
Indicator taxa
Bosea
Rhodovastum
Methylobacterium_Methylorubrum
Caulobacteraceae
Pleomorphomonadaceae
Hydrogenophilaceae
Aquisphaera
Clostridia
Risungbinella
Gracilibacteraceae
Krasilnikovia
Acidimicrobiaceae
Quadrisphaera
Acidobacteriaceae_Subgroup1
Relative abundance (%)
0.00000
0.00025
0.00050
0.00075
Phylum
Pseudomonadota
Planctomycetota
Bacillota
Actinomycetota
Acidobacteriota
D
Soil
Suppressive
Conducive
MS16
MS7
MC10
MC112
Indicator taxa
S−BQ2−57_soil_group
Rhodopila
Pleomorphomonadaceae
Duganella
Methylobacterium_Methylorubrum
Roseisolibacter
Fervidicola
Aneurinibacillus
Endomicrobium
FCPS473
HSB_OF53−F07
1959−1
Aeromicrobium
Arthrobacter
Acidimicrobiaceae
Janibacter
Acidobacteriaceae_Subgroup_1
Relative abundance (%)
0e+00
2e−04
4e−04
6e−04
Phylum
Verrucomicrobiota
Pseudomonadota
Gemmatimonadota
Bacillota
Elusimicrobiota
Chloroflexota
Actinomycetota
Acidobacteriota

Fig. S6

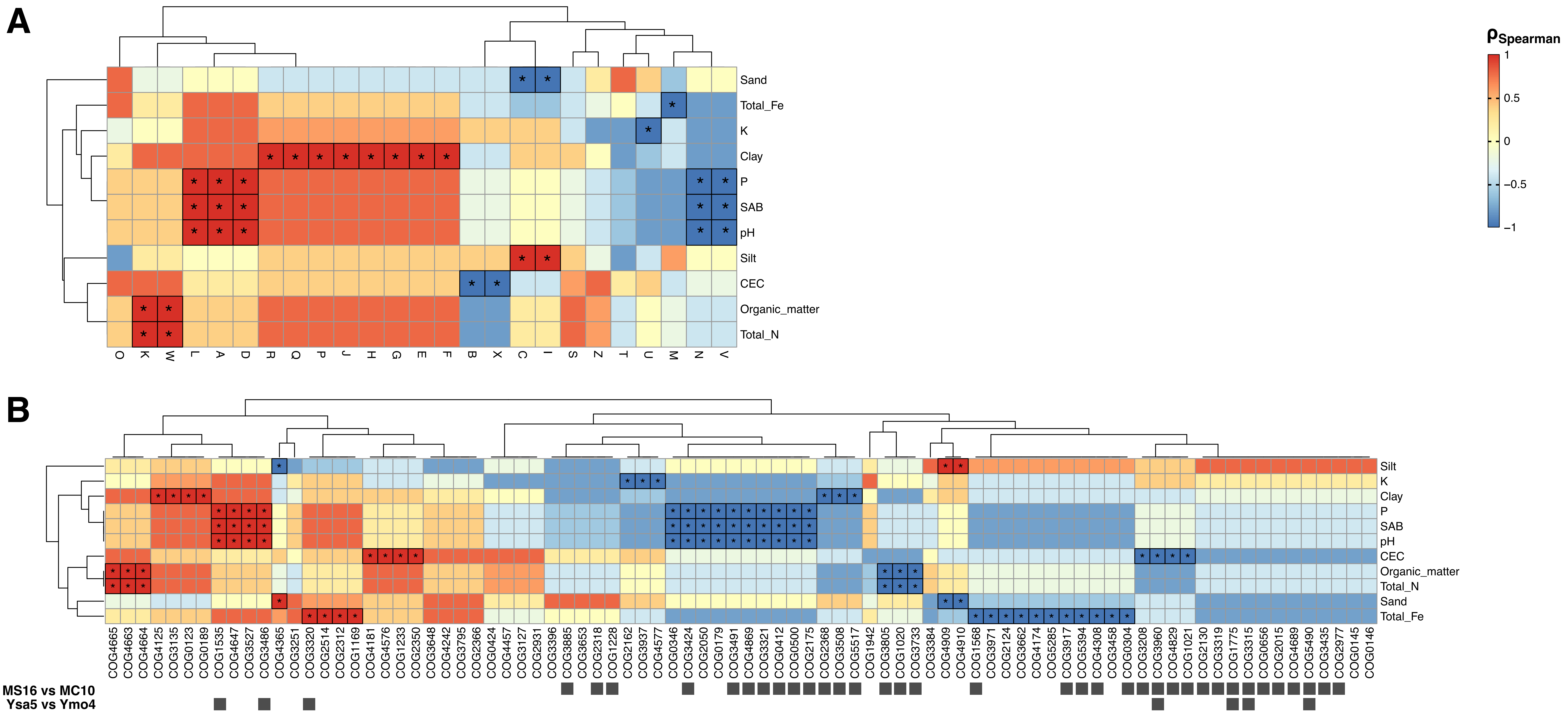
A
ρSpearman
1
0.5
0
−0.5
−1
Sand
Total_Fe
K
Clay
P
SAB
pH
Silt
CEC
Organic_matter
Total_N
O K W L A D R Q P J H G E F B X C I S Z T U M N V
B
Silt
K
Clay
P
SAB
pH
CEC
Organic_matter
Total_N
Sand
Total_Fe
COG4665 COG4663 COG4664 COG4125 COG3135 COG0123 COG0189 COG1535 COG4647 COG3527 COG3486 COG4365 COG3251 COG3320 COG2514 COG2312 COG1169 COG4181 COG4576 COG1233 COG2350 COG3648 COG4242 COG3795 COG2366 COG0424 COG4457 COG3127 COG2931 COG3396 COG3885 COG3653 COG2318 COG1228 COG2162 COG3937 COG4577 COG0346 COG3424 COG2050 COG0179 COG3491 COG4869 COG3321 COG0412 COG0500 COG2175 COG2368 COG3508 COG5517 COG1942 COG3805 COG1020 COG3733 COG3384 COG4909 COG4910 COG1568 COG3971 COG2124 COG3662 COG4174 COG5285 COG3917 COG5394 COG4308 COG3458 COG0304 COG3208 COG3960 COG4829 COG1021 COG2130 COG3319 COG1775 COG3315 COG0656 COG2015 COG4689 COG5490 COG3435 COG2977 COG0145 COG0146
MS16 vs MC10
Ysa5 vs Ymo4

Fig. S7

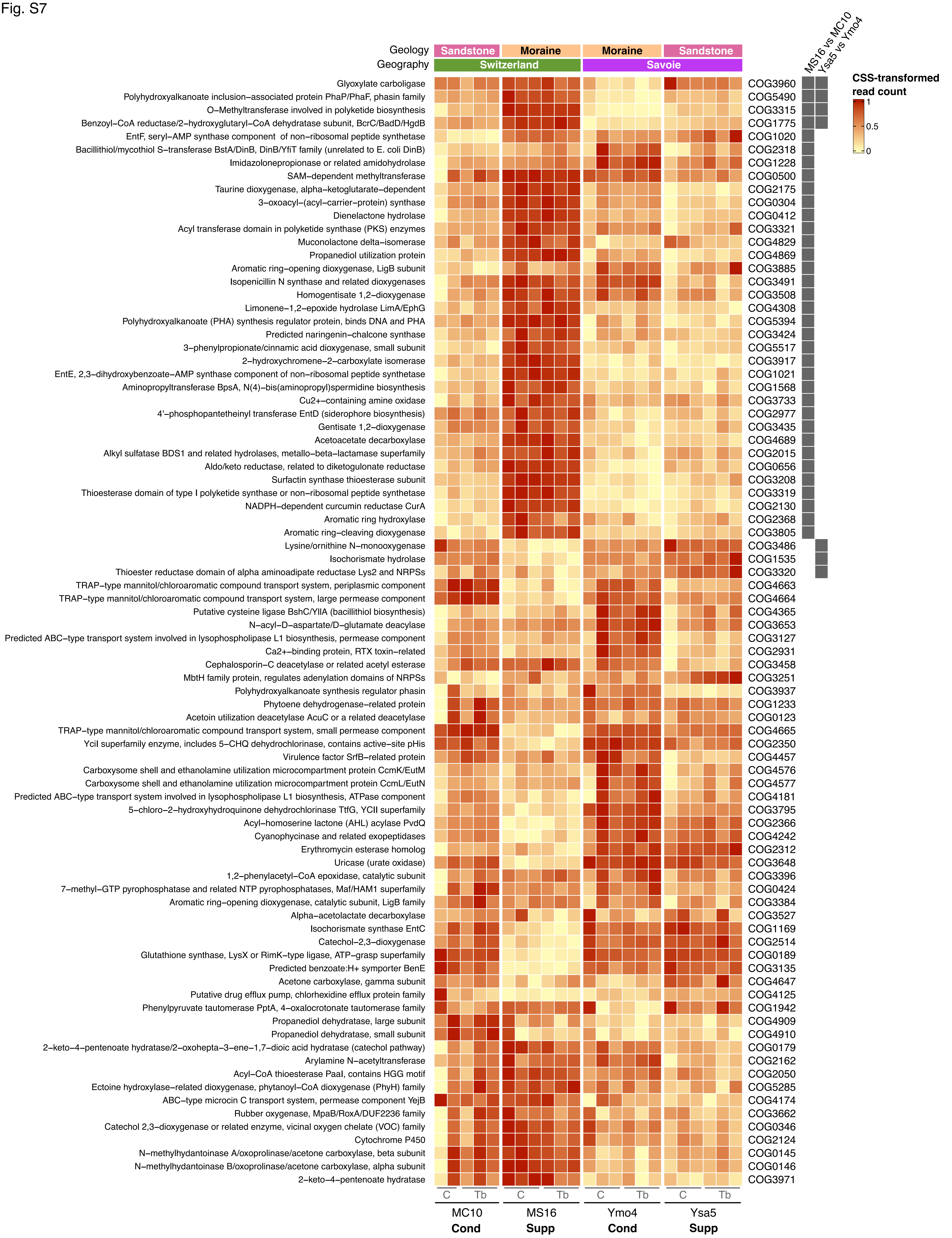

MS16 vs MC10
Ysa5 vs Ymo4
Geology
Sandstone
Moraine
Moraine
Sandstone
Geography
Switzerland
Savoie
CSS-transformed read count
1
0.5
0
Glyoxylate carboligase
Polyhydroxyalkanoate inclusion−associated protein PhaP/PhaF, phasin family
O−Methyltransferase involved in polyketide biosynthesis
Benzoyl−CoA reductase/2−hydroxyglutaryl−CoA dehydratase subunit, BcrC/BadD/HgdB
EntF, seryl−AMP synthase component of non−ribosomal peptide synthetase
Bacillithiol/mycothiol S−transferase BstA/DinB, DinB/YfiT family (unrelated to E. coli DinB)
Imidazolonepropionase or related amidohydrolase
SAM−dependent methyltransferase
Taurine dioxygenase, alpha−ketoglutarate−dependent
3−oxoacyl−(acyl−carrier−protein) synthase
Dienelactone hydrolase
Acyl transferase domain in polyketide synthase (PKS) enzymes
Muconolactone delta−isomerase
Propanediol utilization protein
Aromatic ring−opening dioxygenase, LigB subunit
Isopenicillin N synthase and related dioxygenases
Homogentisate 1,2−dioxygenase
Limonene−1,2−epoxide hydrolase LimA/EphG
Polyhydroxyalkanoate (PHA) synthesis regulator protein, binds DNA and PHA
Predicted naringenin−chalcone synthase
3−phenylpropionate/cinnamic acid dioxygenase, small subunit
2−hydroxychromene−2−carboxylate isomerase
EntE, 2,3−dihydroxybenzoate−AMP synthase component of non−ribosomal peptide synthetase
Aminopropyltransferase BpsA, N(4)−bis(aminopropyl)spermidine biosynthesis
Cu2+−containing amine oxidase
4'−phosphopantetheinyl transferase EntD (siderophore biosynthesis)
Gentisate 1,2−dioxygenase
Acetoacetate decarboxylase
Alkyl sulfatase BDS1 and related hydrolases, metallo−beta−lactamase superfamily
Aldo/keto reductase, related to diketogulonate reductase
Surfactin synthase thioesterase subunit
Thioesterase domain of type I polyketide synthase or non−ribosomal peptide synthetase
NADPH−dependent curcumin reductase CurA
Aromatic ring hydroxylase
Aromatic ring−cleaving dioxygenase
Lysine/ornithine N−monooxygenase
Isochorismate hydrolase
Thioester reductase domain of alpha aminoadipate reductase Lys2 and NRPSs
TRAP−type mannitol/chloroaromatic compound transport system, periplasmic component
TRAP−type mannitol/chloroaromatic compound transport system, large permease component
Putative cysteine ligase BshC/YllA (bacillithiol biosynthesis)
N−acyl−D−aspartate/D−glutamate deacylase
Predicted ABC−type transport system involved in lysophospholipase L1 biosynthesis, permease component
Ca2+−binding protein, RTX toxin−related
Cephalosporin−C deacetylase or related acetyl esterase
MbtH family protein, regulates adenylation domains of NRPSs
Polyhydroxyalkanoate synthesis regulator phasin
Phytoene dehydrogenase−related protein
Acetoin utilization deacetylase AcuC or a related deacetylase
TRAP−type mannitol/chloroaromatic compound transport system, small permease component
YciI superfamily enzyme, includes 5−CHQ dehydrochlorinase, contains active−site pHis
Virulence factor SrfB−related protein
Carboxysome shell and ethanolamine utilization microcompartment protein CcmK/EutM
Carboxysome shell and ethanolamine utilization microcompartment protein CcmL/EutN
Predicted ABC−type transport system involved in lysophospholipase L1 biosynthesis, ATPase component
5−chloro−2−hydroxyhydroquinone dehydrochlorinase TftG, YCII superfamily
Acyl−homoserine lactone (AHL) acylase PvdQ
Cyanophycinase and related exopeptidases
Erythromycin esterase homolog
Uricase (urate oxidase)
1,2−phenylacetyl−CoA epoxidase, catalytic subunit
7−methyl−GTP pyrophosphatase and related NTP pyrophosphatases, Maf/HAM1 superfamily
Aromatic ring−opening dioxygenase, catalytic subunit, LigB family
Alpha−acetolactate decarboxylase
Isochorismate synthase EntC
Catechol−2,3−dioxygenase
Glutathione synthase, LysX or RimK−type ligase, ATP−grasp superfamily
Predicted benzoate:H+ symporter BenE
Acetone carboxylase, gamma subunit
Putative drug efflux pump, chlorhexidine efflux protein family
Phenylpyruvate tautomerase PptA, 4−oxalocrotonate tautomerase family
Propanediol dehydratase, large subunit
Propanediol dehydratase, small subunit
2−keto−4−pentenoate hydratase/2−oxohepta−3−ene−1,7−dioic acid hydratase (catechol pathway)
Arylamine N−acetyltransferase
Acyl−CoA thioesterase PaaI, contains HGG motif
Ectoine hydroxylase−related dioxygenase, phytanoyl−CoA dioxygenase (PhyH) family
ABC−type microcin C transport system, permease component YejB
Rubber oxygenase, MpaB/RoxA/DUF2236 family
Catechol 2,3−dioxygenase or related enzyme, vicinal oxygen chelate (VOC) family
Cytochrome P450
N−methylhydantoinase A/oxoprolinase/acetone carboxylase, beta subunit
N−methylhydantoinase B/oxoprolinase/acetone carboxylase, alpha subunit
2−keto−4−pentenoate hydratase
COG3960
COG5490
COG3315
COG1775
COG1020
COG2318
COG1228
COG0500
COG2175
COG0304
COG0412
COG3321
COG4829
COG4869
COG3885
COG3491
COG3508
COG4308
COG5394
COG3424
COG5517
COG3917
COG1021
COG1568
COG3733
COG2977
COG3435
COG4689
COG2015
COG0656
COG3208
COG3319
COG2130
COG2368
COG3805
COG3486
COG1535
COG3320
COG4663
COG4664
COG4365
COG3653
COG3127
COG2931
COG3458
COG3251
COG3937
COG1233
COG0123
COG4665
COG2350
COG4457
COG4576
COG4577
COG4181
COG3795
COG2366
COG4242
COG2312
COG3648
COG3396
COG0424
COG3384
COG3527
COG1169
COG2514
COG0189
COG3135
COG4647
COG4125
COG1942
COG4909
COG4910
COG0179
COG2162
COG2050
COG5285
COG4174
COG3662
COG0346
COG2124
COG0145
COG0146
COG3971
C
Tb
C
Tb
C
Tb
C
Tb
MC10
Cond
MS16
Supp
Ymo4
Cond
Ysa5
Supp

Fig. S8

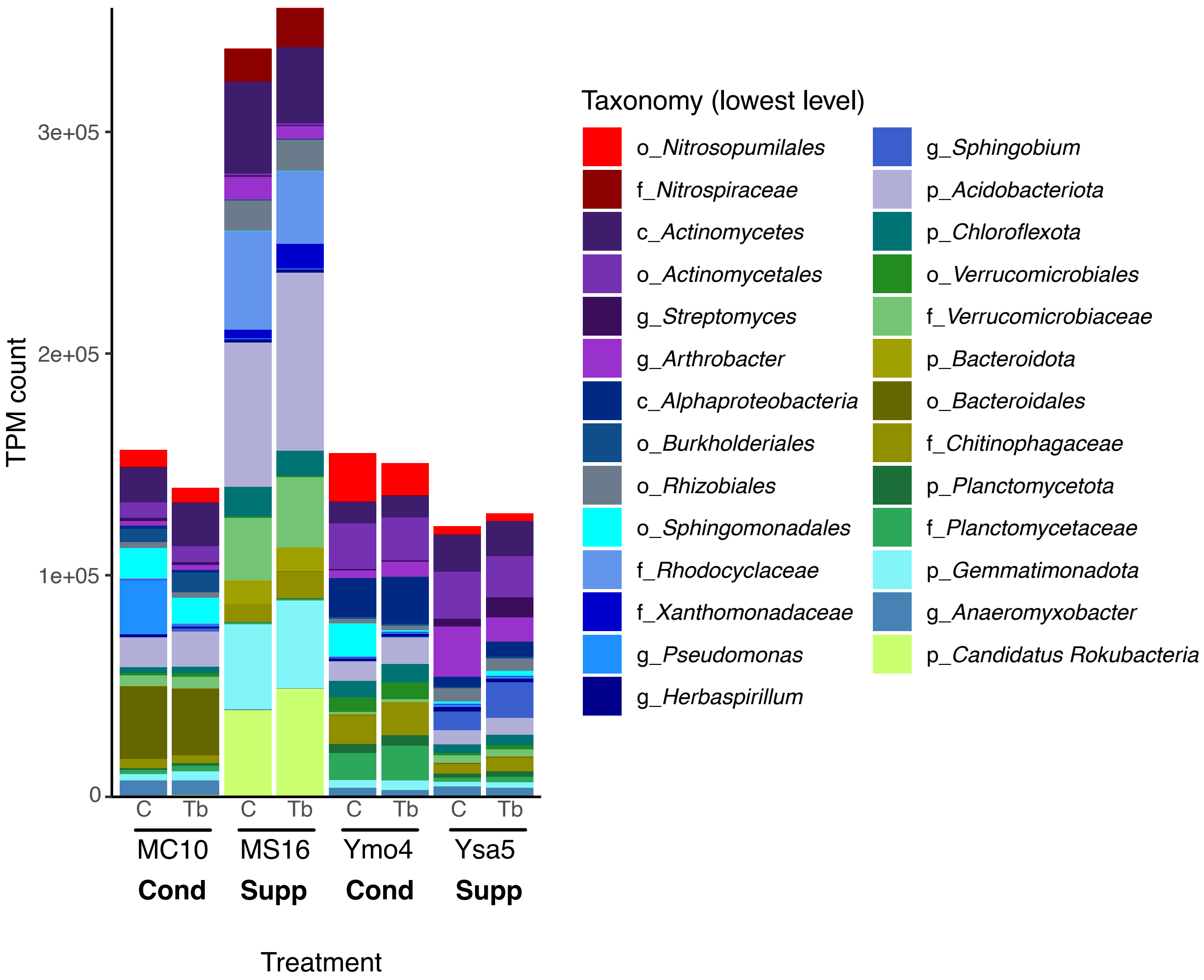

TPM count
3e+05
2e+05
1e+05
0
C
Tb
MC10
MS16
Ymo4
Ysa5
Cond
Supp
Treatment
Taxonomy (lowest level)
o_Nitrosopumilales
f_Nitrospiraceae
c_Actinomycetes
o_Actinomycetales
g_Streptomyces
g_Arthrobacter
c_Alphaproteobacteria
o_Burkholderiales
o_Rhizobiales
o_Sphingomonadales
f_Rhodocyclaceae
f_Xanthomonadaceae
g_Pseudomonas
g_Herbaspirillum
g_Sphingobium
p_Acidobacteriota
p_Chloroflexota
o_Verrucomicrobiales
f_Verrucomicrobiaceae
p_Bacteroidota
o_Bacteroidales
f_Chitinophagaceae
p_Planctomycetota
f_Planctomycetaceae
p_Gemmatimonadota
g_Anaeromyxobacter
p_Candidatus Rokubacteria

Fig. S9

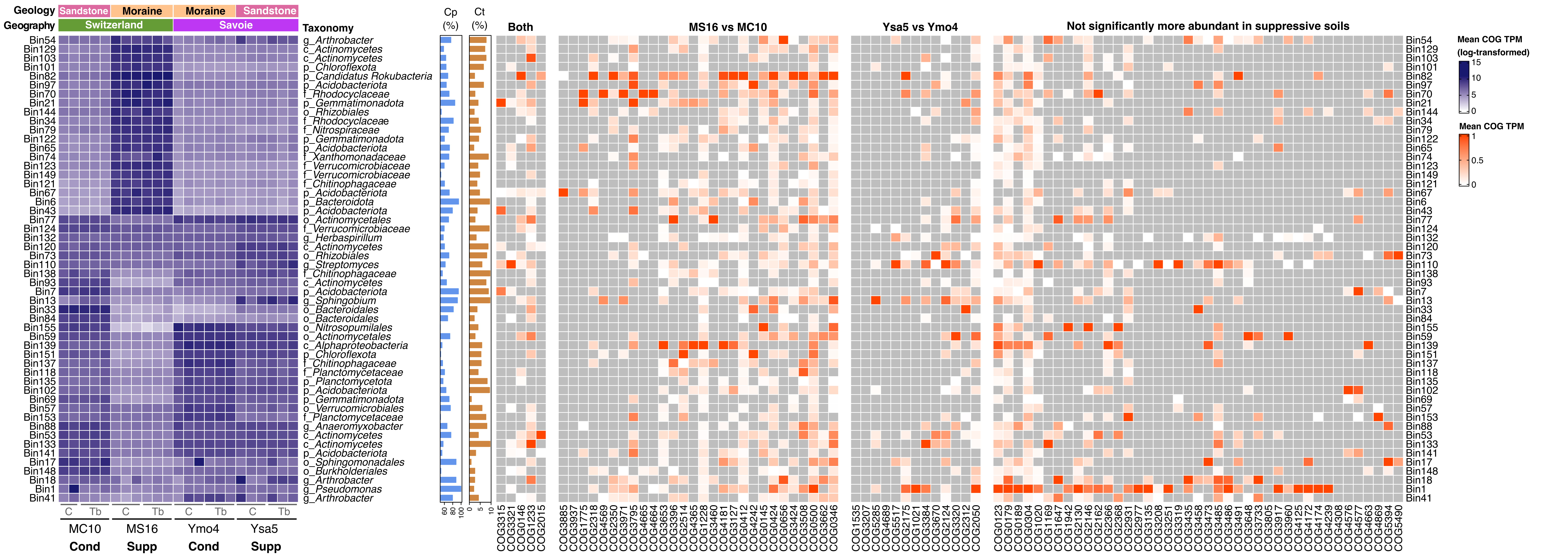

Geology
Sandstone
Moraine
Moraine
Sandstone
Geography
Switzerland
Savoie
Taxonomy
Cp (%)
Ct (%)
Both
MS16 vs MC10
Ysa5 vs Ymo4
Not significantly more abundant in suppressive soils
Mean COG TPM (log-transformed)
15
10
5
0
Mean COG TPM
1
0.5
0
Bin54 g_Arthrobacter
Bin129 c_Actinomycetes
Bin103 c_Actinomycetes
Bin101 p_Chloroflexota
Bin82 p_Candidatus Rokubacteria
Bin97 p_Acidobacteriota
Bin70 f_Rhodocyclaceae
Bin21 p_Gemmatimonadota
Bin144 o_Rhizobiales
Bin34 f_Rhodocyclaceae
Bin79 f_Nitrospiraceae
Bin122 p_Gemmatimonadota
Bin65 p_Acidobacteriota
Bin74 f_Xanthomonadaceae
Bin123 f_Verrucomicrobiaceae
Bin149 f_Verrucomicrobiaceae
Bin121 f_Chitinophagaceae
Bin67 p_Acidobacteriota
Bin6 p_Bacteroidota
Bin43 p_Acidobacteriota
Bin77 o_Actinomycetales
Bin124 f_Verrucomicrobiaceae
Bin132 g_Herbaspirillum
Bin120 c_Actinomycetes
Bin73 o_Rhizobiales
Bin110 g_Streptomyces
Bin138 f_Chitinophagaceae
Bin93 c_Actinomycetes
Bin7 p_Acidobacteriota
Bin13 g_Sphingobium
Bin33 o_Bacteroidales
Bin84 o_Bacteroidales
Bin155 o_Nitrosopumilales
Bin59 o_Actinomycetales
Bin139 c_Alphaproteobacteria
Bin151 p_Chloroflexota
Bin137 f_Chitinophagaceae
Bin118 f_Planctomycetaceae
Bin135 p_Planctomycetota
Bin102 p_Acidobacteriota
Bin69 p_Gemmatimonadota
Bin57 o_Verrucomicrobiales
Bin153 f_Planctomycetaceae
Bin88 g_Anaeromyxobacter
Bin53 c_Actinomycetes
Bin133 c_Actinomycetes
Bin141 p_Acidobacteriota
Bin17 o_Sphingomonadales
Bin148 o_Burkholderiales
Bin18 g_Arthrobacter
Bin1 g_Pseudomonas
Bin41 g_Arthrobacter
C Tb C Tb C Tb C Tb
MC10 MS16 Ymo4 Ysa5
Cond Supp Cond Supp
60 80 100
0 5 10
COG3315 COG3321 COG0146 COG1233 COG2015
COG3885 COG3937 COG1775 COG2318 COG4569 COG2350 COG3971 COG3795 COG4665 COG4664 COG3653 COG3396 COG2514 COG4365 COG1228 COG3460 COG4181 COG3127 COG0412 COG4242 COG0145 COG0424 COG0656 COG3424 COG3508 COG0500 COG3662 COG0346
COG1535 COG3207 COG5285 COG4689 COG5517 COG2175 COG1021 COG3384 COG3670 COG2124 COG3320 COG2312 COG2050
COG0123 COG0179 COG0189 COG0304 COG1020 COG1169 COG1647 COG1942 COG2130 COG2146 COG2162 COG2366 COG2368 COG2931 COG2977 COG3135 COG3208 COG3251 COG3319 COG3435 COG3458 COG3473 COG3485 COG3486 COG3491 COG3648 COG3733 COG3805 COG3917 COG3960 COG4125 COG4172 COG4174 COG4239 COG4308 COG4576 COG4577 COG4663 COG4869 COG5394 COG5490

Fig. S10

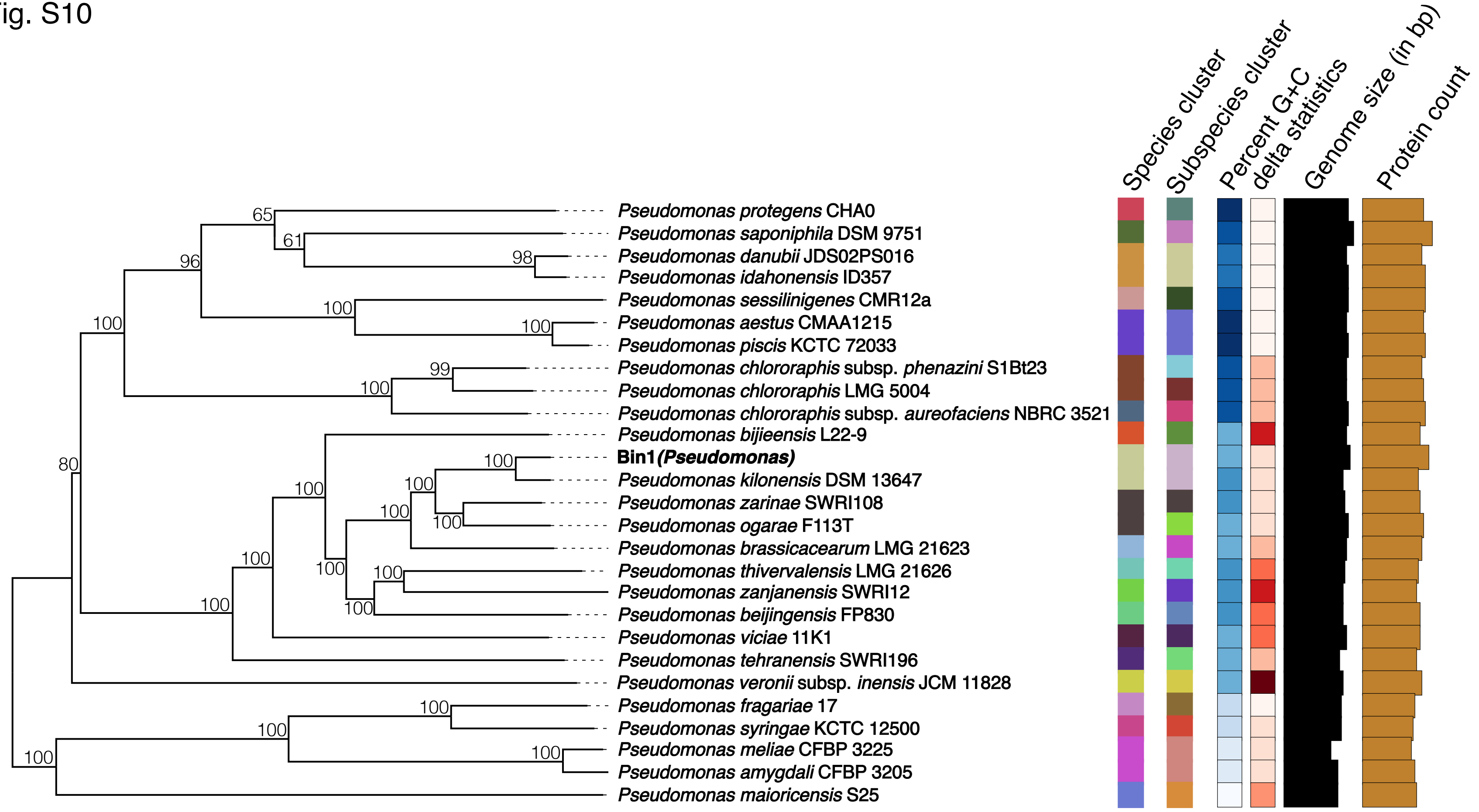

Species cluster
Subspecies cluster
Percent G+C
delta statistics
Genome size (in bp)
Protein count
Pseudomonas protegens CHA0
Pseudomonas saponiphila DSM 9751
Pseudomonas danubii JDS02PS016
Pseudomonas idahonensis ID357
Pseudomonas sessilinigenes CMR12a
Pseudomonas aestus CMAA1215
Pseudomonas piscis KCTC 72033
Pseudomonas chlororaphis subsp. phenazini S1Bt23
Pseudomonas chlororaphis LMG 5004
Pseudomonas chlororaphis subsp. aureofaciens NBRC 3521
Pseudomonas bijieensis L22-9
Bin1 (Pseudomonas)
Pseudomonas kilonensis DSM 13647
Pseudomonas zarinae SWRI108
Pseudomonas ogarae F113T
Pseudomonas brassicacearum LMG 21623
Pseudomonas thivervalensis LMG 21626
Pseudomonas zanjanensis SWRI12
Pseudomonas beijingensis FP830
Pseudomonas viciae 11K1
Pseudomonas tehranensis SWRI196
Pseudomonas veronii subsp. inensis JCM 11828
Pseudomonas fragariae 17
Pseudomonas syringae KCTC 12500
Pseudomonas meliae CFBP 3225
Pseudomonas amygdali CFBP 3205
Pseudomonas maioricensis S25

Fig. S11

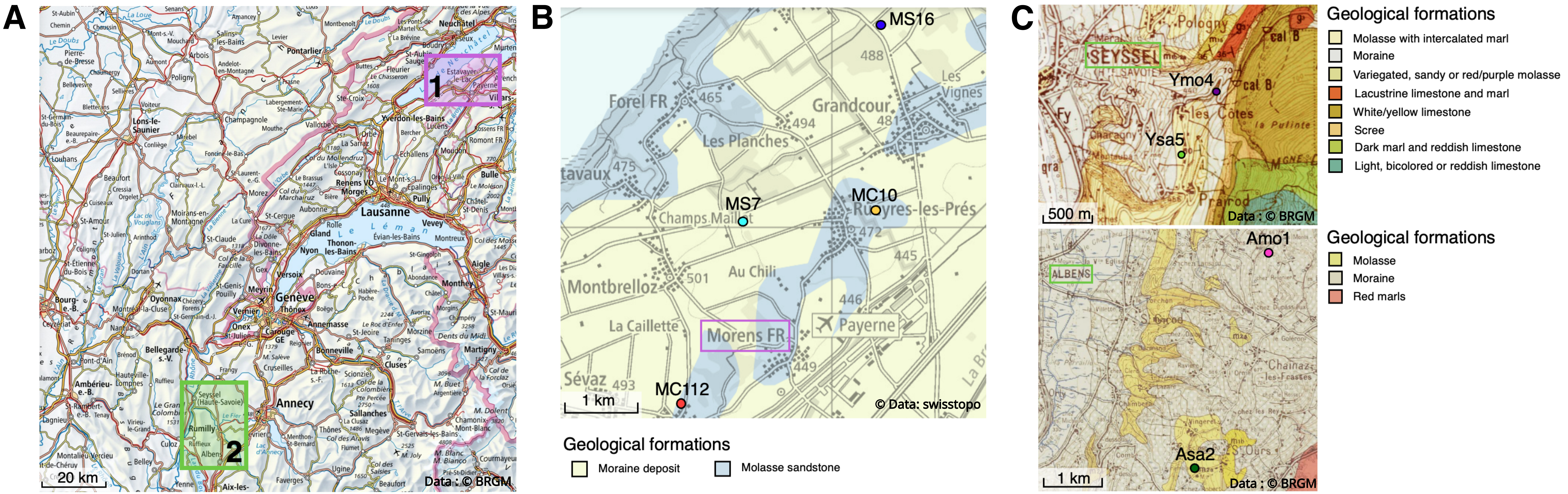
A
1
2
20 km
Data : © BRGM
B
MS16
MS7
MC10
MC112
Morens FR
Payerne
1 km
© Data: swisstopo
Geological formations
Moraine deposit
Molasse sandstone
C
SEYSSEL
Ymo4
Ysa5
500 m
Data : © BRGM
Geological formations
Molasse with intercalated marl
Moraine
Variegated, sandy or red/purple molasse
Lacustrine limestone and marl
White/yellow limestone
Scree
Dark marl and reddish limestone
Light, bicolored or reddish limestone
ALBENS
Amo1
Asa2
1 km
Data : © BRGM
Geological formations
Molasse
Moraine
Red marls
