# Supplementary material for: Biogeography influences plant–microbe interactions and natural soil suppressiveness to black root rot disease of tobacco
Source: Genome Biol. 2025 Dec 28;27:16. doi: 10.1186/s13059-025-03911-0 (PMC12857086; doi:10.1186/s13059-025-03911-0)
Supplement: Supplementary file 2 — Additional file 2: Table S1. PERMANOVA results for the soil metabolome, with two models. Table S2. PERMANOVA results for the ITS metabarcoding, with three models. Table S3. PERMANOVA results for the 16S metabarcoding, with all soils and three models and separate soils for which the effect of pathogen inoculation was significant. Table S4. Comparison of most abundant phyla in the metagenomes of suppressive and conducive soils. Table S5. Comparison of most abundant classes in the metagenomes of suppressive and conducive soil samples. Table S6. PERMANOVA results for the abundance of COGs in the metagenome. Table S7. Comparison of the abundance of COG main categories between soils and number of differentially abundant COGs within the categories. Table S8. Comparison of the abundance and differential abundance analysis of individual COGs belonging to the ‘Q’ main COG category. Table S9. Details on the bins reconstructed with the coassembled tobacco rhizosphere metagenomes. Table S10. AntiSMASH annotation for bins that are > 50% complete and < 10% contaminated. Table S11. Subject strains used for the comparison with the Pseudomonas bin 1 with dDDH values and G + C difference when compared to Bin 1. Table S12. PERMANOVA results for the metabolome of tobacco shoots. Table S13. Main physicochemical characteristics of Swiss and Savoie soils. Table S14. Quality control for the shotgun metagenome assembly and binning. [file 13059_2025_3911_MOESM2_ESM.pdf]

Additional file 2

Biogeography influences plant-microbe interactions and natural soil suppressiveness to black root rot disease of tobacco

Alix Catry[1], Danis Abrouk[1], Nicolas Fierling[1], Ana Isabel Serrano Mendoza[1], Marjolaine Rey[1], Pilar Vesga[2], Clara M. Heiman[2], Daniel Garrido-Sanz[2], Marie-Lara Bouffaud[3], François Buscot[3], Adriana Giongo[4], Kornelia Smalla[4], Gilles Comte[1], Christoph Keel[2], Daniel Muller[1] and Yvan Moënne-Loccoz[1,5]*

[1]Université Claude Bernard Lyon 1, CNRS, INRAE, VetAgro Sup, UMR5557 Ecologie Microbienne, 43 bd du 11 novembre 1918, F-69622 Villeurbanne, France

[2]University of Lausanne, Department of Fundamental Microbiology, Quartier UNIL-Sorge, CH-1015 Lausanne, Switzerland

[3]Department of Soil Ecology, Helmholtz Centre for Environmental Research - UFZ, Theodor-Lieser-Str. 4, D-06120 Halle/Saale, Germany

[4] Institute for Epidemiology and Pathogen Diagnostics, Julius Kühn Institute (JKI) – Federal Research Centre for Cultivated Plants, Messeweg 11-12, D-38104 Braunschweig, Germany

[5]Institut Universitaire de France (IUF), F-75005 Paris, France

Supplementary tables

Table S1. PERMANOVA results for the soil metabolome, with two models.

Model	Source of variation	df	F	*P*	η^2 (%)
(Origin × Geology)/ Soil	Geography	1	6.79	<0.001	7.83
	Geology	1	5.90	<0.001	6.81
	Geography:Geology	1	3.79	<0.001	4.37
	Geography:Geology:Soil	4	3.56	<0.001	16.4
	Residual	56			
	Total	63			
Geography × Status	Geography	1	5.79	<0.001	7.83
	Status	1	3.24	0.002	4.37
	Geology:Status	1	5.04	<0.001	6.81
	Residual	60			
	Total	63			

Table S2. PERMANOVA results for the ITS metabarcoding, with three models.

Model	Source of variation	df	R^2	F	*P*
Soil × Inoculation	Soil	7	0.68	6.79	<0.001
	Inoculation	1	0.03	5.90	<0.001
	Soil:Inoculation	7	0.03	3.79	0.18
	Residual	70	0.25		
	Total	85	1		
Geology × Status	Geology	1	0.11	13.96	<0.001
	Status	1	0.09	10.76	<0.001
	Geology:Status	1	0.15	18.53	<0.001
	Residual	82	0.65		
	Total	85	1		
Geography × Geology	Geography	1	0.15	19.00	<0.001
	Geology	1	0.11	14.69	<0.001
	Geography:Geology	1	0.08	10.56	<0.001
	Residual	82	0.66		
	Total	85	1		

Table S3. PERMANOVA results for the 16S metabarcoding, with (i) all soils and three models and (ii) separate soils for which the effect of pathogen inoculation was significant.

Soil	Model	Source of variation	df	R^2	F	*P*
All	Soil × Inoculation	Soil	7	0.63	18.93	<0.001
		Inoculation	1	0.007	1.45	0.14
		Soil:Inoculation	7	0.05	1.39	0.049
		Residual	67	0.31		
		Total	82	1		
All	Geology × Status	Geology	1	0.10	11.53	<0.001
		Status	1	0.05	6.18	<0.001
		Geology:Status	1	0.19	22.89	<0.001
		Residual	79	0.66		
		Total	82	1		
All	Geography × Geology	Geography	1	0.19	23.21	<0.001
		Status	1	0.09	11.34	<0.001
		Geography:Geology	1	0.05	6.06	<0.001
		Residual	79	0.66		
		Total	82	1		
MS16	Inoculation	Inoculation	1	0.17	1.82	0.034
		Residual	9	0.83		
		Total	10	1		
Ysa5	Inoculation	Inoculation	1	0.15	1.46	0.046
		Residual	8	0.85		
		Total	9	1		
Amo1	Inoculation	Inoculation	1	0.17	1.88	0.03
		Residual	9	0.83		
		Total	10	1		

Table S4. Comparison of most abundant phyla in the metagenomes of suppressive and conducive soils.

Phylum	MS16 vs MC10		Ysa5 vs Ymo4		MS16 vs Ysa5	
	**Adjusted *P* value**[1]	**Difference**[2]	**Adjusted *P* value**	**Difference**	**Adjusted *P* value**	**Difference**
Nitrososphaerota	0.0079	-77.6%	0.090		0.0040	-83.4%
Actinomycetota	0.012	-21.6%	0.59		0.021	-20.2%
Acidobacteriota	0.0079	+23.9%	0.12		0.021	+14.7%
Pseudomonadota	0.20		0.43		0.0040	+14.1%
Verrucomicrobiota	0.0079	+356%	0.090		0.0040	+423%
Chloroflexota	0.0079	-48.2%	0.59		0.0040	-34.9%
Bacteroidota	0.012	-31.7%	0.59		0.037	-36.9%
Myxococcota	0.66		0.090		0.071	
Planctomycetota	0.037	-17.2%	0.090		0.13	
Gemmatimonadota	0.0079	-16.7%	0.38		0.0040	-50.0%
Candidatus Rokubacteria	0.0079	+66%	0.090		0.0040	-51.6%

[1]Computed with Wilcoxon signed-rank tests (FDR correction) by comparing TPM values between soils.

[2]A positive value indicates that the phylum is significantly more abundant in the first than in the second soil. Non-significant differences were not computed.

Table S5. Comparison of most abundant classes in the metagenomes of suppressive and conducive soil samples.

Phylum	Class	MS16 vs MC10		Ysa5 vs Ymo4		MS16 vs Ysa5	
		Adj. *P* value[1]	Difference (%)[2]	Adj. *P* value	Difference (%)	Adj. *P* value	Difference (%)
Pseudomonadota	Alphaproteobacteria	0.024	14.6	0.036	11.0	0.0027	21.3
Actinomycetota	Actinomycetes	0.33		0.036	38.4	0.0094	-24.6
Pseudomonadota	Betaproteobacteria	0.036	20.6	0.73		0.0027	27.1
Nitrososphaerota	Nitrososphaeria	0.0080	-73.9	0.052		0.0027	-82.3
Actinomycetota	Thermoleophilia	0.0080	-44.8	0.73		0.0027	-41.7
Actinomycetota	Acidimicrobiia	0.0080	-55.4	0.016	-27.1	0.0027	-40.4
Pseudomonadota	Gammaproteobacteria	0.0080	-56.7	0.052		0.0049	-47.3
No phylum in NCBI	Deltaproteobacteria	0.012	-33.2	0.016	45.1	0.0049	-32.4
Planctomycetota	Planctomycetia	0.012	-23.6	0.076		0.48	
Bacteroidota	Chitinophagia	0.036	-32.5	0.94		0.0027	-66.4
Actinomycetota	Rubrobacteria	0.0080	-93.3	0.46		0.0027	-94.1
Planctomycetota	Phycisphaerae	0.059		0.016	-47.1	0.0027	43.7
Myxococcota	Myxococcia	0.012	50.7	0.094		0.0027	49.2
Acidobacteriota	Terriglobia	0.0080	915.7	0.094		0.0027	1181.4
Thermodesulfobacteriota	Desulfuromonadia	0.0080	65.2	0.016	98.4	0.0027	1314.2
Bacteroidota	Cytophagia	0.0080	-93.9	0.094		0.0027	-95.6
Nitrospirota	Nitrospiria	0.0080	-66.0	0.016	54.9	0.0027	-72.2
Bacteroidota	Bacteroidia	0.0080	-49.6	0.18		0.0027	1420.2
Bacillota	Bacilli	0.26		0.016	297	0.0027	-67.8
Chloroflexota	Ktedonobacteria	0.012	-46.9	0.094		0.0027	-63.3
Gemmatimonadota	Gemmatimonadetes	0.0080	-84.5	0.025	45.2	0.0027	-89.9
Bacteroidota	Flavobacteriia	0.14		0.025	187	0.0027	-77.5
Bacteroidota	Saprospiria	0.0080	-98.9	0.23		0.0027	-97.2
Bacillota	Clostridia	0.036	-39.6	0.38		0.25	
Acidobacteriota	Vicinamibacteria	0.0080	-89.8	0.016	-33.6	0.0027	-85.0
Thermomicrobiota	Thermomicrobia	0.0080	-94.9	0.55		0.0027	-95.9

[1]Computed with Wilcoxon signed-rank tests (FDR correction) by comparing TPM values between soils.

[2]A positive value indicates that the class is significantly more abundant in the first than in the second soil. Non-significant differences were not computed.

Table S6. PERMANOVA results for the abundance of COGs in the metagenome.

Model	Source	R^2	F	*P*
Geography × Status × Inoculation	**Geography**	**0.230**	**8.59**	**5e-04 (***)**
	**Status**	**0.131**	**4.89**	**0.0077 (**)**
	Inoculation	0.056	2.1	0.126
	**Geography:Status**	**0.154**	**5.76**	**0.0046 (**)**
	Geography:Inoculation	0.017	0.647	0.554
	Status:Inoculation	0.007	0.256	0.867
	Geography:Status:Inoculation	0.004	0.133	0.952
	Residual	0.401		
Geography × Geology × Inoculation	**Geography**	**0.230**	**8.59**	**5e-04 (***)**
	**Geology**	**0.148**	**5.52**	**0.0047 (**)**
	Inoculation	0.052	1.94	0.134
	**Geography: Geology**	**0.141**	**5.28**	**0.0054 (**)**
	Geography:Inoculation	0.017	0.647	0.554
	Geology:Inoculation	0.004	0.156	0.942
	Geography: Geology:Inoculation	0.006	0.233	0.88
	Residual	0.401		

Table S7. Comparison of the abundance of COG main categories between soils and number of differentially abundant COGs within the categories.

COG Group	COG Category	Category description	MS16 vs MC10		Ysa5 vs Ymo4		MS16 vs Ysa5		Swiss vs Savoie soils	
			Number of DA COG[1]	logFC[2]	Number of DA COG	logFC	Number of DA COG	logFC	Number of DA COG	logFC
1	J	Translation, ribosomal structure and biogenesis	49	-0.619	143	-1.049	142	-1.914	270	-5.805
1	A	RNA processing and modification	0	-2.792	0	-6.081	0	-8.056	4	-16.044
1	K	Transcription	22	-1.386	59	-2.363	65	-3.715	120	-4.634
1	L	Replication, recombination and repair	24	1.087	58	1.773	76	0.375	129	0.556
1	B	Chromatin structure and dynamics	1	6.243	3	-3.850	3	1.085	6	3.986
2	D	Cell cycle control, cell division, chromosome partitioning	10	1.031	27	2.813	27	0.286	39	0.199
2	V	Defense mechanisms	20	1.189	35	3.767	41	3.214	104	4.674
2	T	Signal transduction mechanisms	41	1.661	80	3.002	74	2.089	139	4.117
2	M	Cell wall/membrane/envelope biogenesis	62	3.157	109	5.125	105	4.408	177	5.612
2	N	Cell motility	12	1.042	30	7.565	21	3.231	56	7.741
2	Z	Cytoskeleton	1	8.300	4	16.005	1	12.911	4	11.853
2	W	Extracellular structures	2	0.902	9	4.126	9	3.825	21	4.775
2	U	Intracellular trafficking, secretion, and vesicular transport	18	-0.353	41	3.944	39	1.759	86	2.329
2	O	Posttranslational modification, protein turnover, chaperones	48	2.263	79	3.544	80	3.680	146	4.717
2	X	Mobilome: prophages, transposons	7	2.653	16	6.321	18	6.187	66	14.751
3	C	Energy production and conversion	52	2.200	112	2.669	115	2.651	188	3.334
3	G	Carbohydrate transport and metabolism	51	2.513	112	4.102	105	2.623	169	3.222
3	E	Amino acid transport and metabolism	53	1.737	125	2.193	128	2.393	195	1.652
3	F	Nucleotide transport and metabolism	11	1.199	45	0.756	51	1.570	83	0.239
3	H	Coenzyme transport and metabolism	25	0.227	77	-0.461	97	-0.420	179	-1.814
3	I	Lipid transport and metabolism	26	2.548	69	3.114	69	3.182	114	4.153
3	P	Inorganic ion transport and metabolism	43	0.309	88	1.174	82	0.073	167	-0.667
3	Q	Secondary metabolites biosynthesis, transport and catabolism	17	2.197	41	2.283	36	3.007	68	4.429
4	R	General function prediction only	58	0.569	146	1.680	146	0.763	326	-0.024
4	S	Function unknown	34	-2.022	95	-0.163	86	-1.620	265	0.140

[1]Computed with eBayes on a model fit generated by the fitZig function of the metagenomeSeq package; [2]log-transformed fold change

Table S8. Comparison of the abundance and differential abundance analysis of individual COGs belonging to the 'Q' main COG category.

COG ID	COG name	Protein	MS16 vs Ysa5		CH vs Savoie	
			logFC[1]	Adj. *P* value[2]	logFC	Adj. *P* value
COG0123	Acetoin utilization deacetylase AcuC or a related deacetylase	AcuC	4.02	0.0489	3.59	0.00351
COG0145	*N*-methylhydantoinase A/oxoprolinase/acetone carboxylase, beta subunit	HyuA	4.16	0.064	6.5	0.000518
COG0146	*N*-methylhydantoinase B/oxoprolinase/acetone carboxylase, alpha subunit	HyuB	4.91	0.0321	7.73	0.000127
COG0179	2-keto-4-pentenoate hydratase/2-oxohepta-3-ene-1,7-dioic acid hydratase (catechol pathway)	YcgM	4.98	0.0176	8.1	3.48e-06
COG0189	Glutathione synthase, LysX or RimK-type ligase, ATP-grasp superfamily	LysX	-0.585	0.726	-5.85	0.00538
COG0304	3-oxoacyl-(acyl-carrier-protein) synthase	FabB	9.65	0.00107	13.4	1.87e-06
COG0346	Catechol 2,3-dioxygenase or related enzyme, vicinal oxygen chelate (VOC) family	GloA	3.89	0.081	7.85	0.000106
COG0412	Dienelactone hydrolase	DLH	7.9	0.00131	12.9	2.21e-06
COG0424	7-methyl-GTP pyrophosphatase and related NTP pyrophosphatases, Maf/HAM1 superfamily	Maf	3.98	0.0308	4.18	0.00586
COG0500	SAM-dependent methyltransferase	SmtA	6.31	0.00418	9.61	3.48e-06
COG0656	Aldo/keto reductase, related to diketogulonate reductase	ARA1	1.04	0.574	9.18	0.000422
COG1020	EntF, seryl-AMP synthase component of non-ribosomal peptide synthetase	EntF	16.4	0.00143	16	2.33e-06
COG1021	EntE, 2,3-dihydroxybenzoate-AMP synthase component of non-ribosomal peptide synthetase	EntE	-3.74	0.412	15.2	0.00258
COG1169	Isochorismate synthase EntC	MenF	-12.6	0.0072	-13.1	6.37e-05
COG1228	Imidazolonepropionase or related amidohydrolase	HutI	15.4	0.00112	14.5	1.27e-06
COG1233	Phytoene dehydrogenase-related protein		4.72	0.023	5.52	0.000254
COG1535	Isochorismate hydrolase	EntB1	5.72	0.779	-25.1	0.00311
COG1568	Aminopropyltransferase BpsA, *N*(4)-bis(aminopropyl)spermidine biosynthesis	BpsA	-7.98	0.498	13.8	0.0467
COG1647	Esterase/lipase	YvaK	-5.23	0.15	0.922	0.655
COG1775	Benzoyl-CoA reductase/2-hydroxyglutaryl-CoA dehydratase subunit, BcrC/BadD/HgdB	HgdB	13.8	0.0203	22.5	0.000442
COG1942	Phenylpyruvate tautomerase PptA, 4-oxalocrotonate tautomerase family	PptA	-10.1	0.0407	-3.69	0.369
COG2015	Alkyl sulfatase BDS1 and related hydrolases, metallo-beta-lactamase superfamily	BDS1	1.84	0.629	7.72	0.00682
COG2050	Acyl-CoA thioesterase PaaI, contains HGG motif	PaaI	2.51	0.415	6.73	0.00106
COG2124	Cytochrome P450	CypX	3.16	0.265	7.57	0.000515
COG2130	NADPH-dependent curcumin reductase CurA	CurA	3.92	0.107	11	0.00018
COG2146	Ferredoxin subunit of nitrite reductase or a ring-hydroxylating dioxygenase	NirD	-0.51	0.854	0.343	0.789
COG2162	Arylamine *N*-acetyltransferase	NhoA	3.69	0.437	6.09	0.0203
COG2175	Taurine dioxygenase, alpha-ketoglutarate-dependent	TauD	9.19	0.00694	18.4	1.76e-05
COG2312	Erythromycin esterase homolog	YbfO	4.25	0.149	3.7	0.00525

COG2318	Bacillithiol/mycothiol S-transferase BstA/DinB, DinB/YfiT family (unrelated to *E. coli* DinB)	DinB	11.3	0.0021	8.73	7.75e-05
COG2350	YciI superfamily enzyme, includes 5-CHQ dehydrochlorinase, contains active-site pHis	YciI	-5.15	0.237	-6.27	0.0357
COG2366	Acyl-homoserine lactone (AHL) acylase PvdQ	PvdQ	10.2	0.00692	2.9	0.218
COG2368	Aromatic ring hydroxylase	YoaI	2.66	0.426	8.61	0.000135
COG2514	Catechol-2,3-dioxygenase	CatE	-0.756	0.776	-2.62	0.0369
COG2931	Ca^{2+}-binding protein, RTX toxin-related		15.1	0.00162	10.4	0.00687
COG2977	4'-phosphopantetheinyl transferase EntD (siderophore biosynthesis)	EntD	-4.4	0.404	1.64	0.599
COG3127	Predicted ABC-type transport system involved in lysophospholipase L1 biosynthesis, permease component	YbbP	10.4	0.00107	8.25	0.00135
COG3135	Predicted benzoate:H^+ symporter BenE	BenE	-2.84	0.456	-11.1	0.000489
COG3207	Pyoverdine/dityrosine biosynthesis protein Dit1	Dit1	13.3	0.595	-0.455	0.969
COG3208	Surfactin synthase thioesterase subunit	GrsT	10.8	0.0359	16.8	0.000957
COG3251	MbtH family protein, regulates adenylation domains of NRPSs	MbtH	2.47	0.72	5.27	0.0357
COG3315	O-Methyltransferase involved in polyketide biosynthesis	YktD	2.28	0.422	13	0.00128
COG3319	Thioesterase domain of type I polyketide synthase or non-ribosomal peptide synthetase	EntF2	7.48	0.0397	16	0.000339
COG3320	Thioester reductase domain of alpha aminoadipate reductase Lys2 and NRPSs	Lys2b	6.31	0.0465	2.55	0.0702
COG3321	Acyl transferase domain in polyketide synthase (PKS) enzymes	PksD	14.3	0.00357	14.6	2e-06
COG3384	Aromatic ring-opening dioxygenase, catalytic subunit, LigB family	LigB	6.86	0.0517	4.71	0.00407
COG3396	1,2-phenylacetyl-CoA epoxidase, catalytic subunit	YdbO	7.16	0.0111	7.48	4.26e-05
COG3424	Predicted naringenin-chalcone synthase	BH0617	2.72	0.359	10.8	3.4e-05
COG3435	Gentisate 1,2-dioxygenase		3.84	0.259	6.82	0.000382
COG3458	Cephalosporin-C deacetylase or related acetyl esterase	Axe1	9.04	0.0204	9.45	0.000149
COG3460	1,2-phenylacetyl-CoA epoxidase, PaaB subunit	PaaB	1.26	0.839	0.508	0.848
COG3473	Maleate cis-trans isomerase		4.5	0.206	2.56	0.134
COG3485	Protocatechuate 3,4-dioxygenase beta subunit	PcaH	2.56	0.317	1.66	0.172
COG3486	Lysine/ornithine *N*-monooxygenase	IucD	-17.8	0.224	-35.9	0.000199
COG3491	Isopenicillin N synthase and related dioxygenases	PcbC	4.32	0.214	10.4	0.000154
COG3508	Homogentisate 1,2-dioxygenase	HmgA	7.2	0.0514	11.3	1.46e-05
COG3527	Alpha-acetolactate decarboxylase	AlsD	-16.3	0.125	-6.93	0.0587
COG3648	Uricase (urate oxidase)	UriC	-0.425	0.916	-6.17	0.0202
COG3653	*N*-acyl-D-aspartate/D-glutamate deacylase		14.3	0.00107	11.7	1.2e-05
COG3662	Rubber oxygenase, MpaB/RoxA/DUF2236 family	MpaB	-4.3	0.216	0.988	0.668
COG3670	Carotenoid cleavage dioxygenase or a related enzyme		3.59	0.369	-7.64	0.0954

COG3733	Cu^{2+}-containing amine oxidase	TynA	-2.88	0.391	7.84	0.00994
COG3795	5-chloro-2-hydroxyhydroquinone dehydrochlorinase TtfG, YCII superfamily	YCII	6.85	0.0196	4.55	0.0119
COG3805	Aromatic ring-cleaving dioxygenase	DodA	5.99	0.118	12.9	0.000739
COG3885	Aromatic ring-opening dioxygenase, LigB subunit		8.89	0.0788	7.26	0.000776
COG3917	2-hydroxychromene-2-carboxylate isomerase	NahD	3.27	0.102	9.97	0.00101
COG3937	Polyhydroxyalkanoate synthesis regulator phasin	PhaF	-6.04	0.244	-1.7	0.713
COG3960	Glyoxylate carboligase	Gcl	-8.69	0.13	-0.861	0.852
COG3971	2-keto-4-pentenoate hydratase	MhpD	0.676	0.779	4.9	0.00228
COG4125	Putative drug efflux pump, chlorhexidine efflux protein family	BTP	-2.3	0.917	-53	0.00661
COG4172	ABC-type microcin C transport system, duplicated ATPase component YejF	YejF	-2.52	0.477	1.08	0.374
COG4174	ABC-type microcin C transport system, permease component YejB	YejB	1.27	0.705	4.04	0.0145
COG4181	Predicted ABC-type transport system involved in lysophospholipase L1 biosynthesis, ATPase component	YbbA	7.12	0.0163	4.27	0.0512
COG4239	ABC-type microcin C transport system, permease component YejE	YejE	0.45	0.882	2.77	0.0566
COG4242	Cyanophycinase and related exopeptidases	CphB	9.19	0.023	1.44	0.569
COG4308	Limonene-1,2-epoxide hydrolase LimA/EphG	LimA	2.6	0.7	11.8	0.00895
COG4365	Putative cysteine ligase BshC/YllA (bacillithiol biosynthesis)	BshC	18.1	0.00365	11.2	0.000481
COG4457	Virulence factor SrfB-related protein	SrfB	15.3	0.0422	2.79	0.677
COG4458	Virulence factor SrfC-related protein	SrfC	1.5	0.88	-2.77	0.68
COG4542	PduX protein involved in propanediol utilization and related proteins	PduX	4.78	0.713	6.36	0.24
COG4569	Acetaldehyde dehydrogenase (acetylating)	MhpF	0.237	0.971	4.03	0.129
COG4576	Carboxysome shell and ethanolamine utilization microcompartment protein CcmK/EutM	CcmL	14.1	0.00662	7.95	0.0167
COG4577	Carboxysome shell and ethanolamine utilization microcompartment protein CcmL/EutN	CcmK	15.5	0.0024	9.75	0.00183
COG4647	Acetone carboxylase, gamma subunit	AcxC	-8.97	0.444	-12.5	0.00989
COG4663	TRAP-type mannitol/chloroaromatic compound transport system, periplasmic component	FcbT1	6.81	0.00632	2.89	0.0734
COG4664	TRAP-type mannitol/chloroaromatic compound transport system, large permease component	FcbT3	7.06	0.0184	0.0922	0.97
COG4665	TRAP-type mannitol/chloroaromatic compound transport system, small permease component	FcbT2	5.31	0.171	-4.44	0.157
COG4689	Acetoacetate decarboxylase	Adc	-3.28	0.513	13.3	0.0167
COG4829	Muconolactone delta-isomerase	CatC1	-25.7	0.0129	-0.61	0.93
COG4869	Propanediol utilization protein	PduL	22.2	0.00141	42.9	2.72e-06
COG4909	Propanediol dehydratase, large subunit	PduC	-7.13	0.267	-5.14	0.0128
COG4910	Propanediol dehydratase, small subunit	PduE	-11.2	0.132	-10.1	0.000276
COG5285	Ectoine hydroxylase-related dioxygenase, phytanoyl-CoA dioxygenase (PhyH) family	PhyH	5.01	0.102	6.09	0.000946

COG5310	Homospermidine synthase		3.26	0.507	5.13	0.059
COG5394	Polyhydroxyalkanoate (PHA) synthesis regulator protein, binds DNA and PHA		3.63	0.147	11.4	8.42e-06
COG5490	Polyhydroxyalkanoate inclusion-associated protein PhaP/PhaF, phasin family	Phasin	-6.99	0.146	8.43	0.0738
COG5517	3-phenylpropionate/cinnamic acid dioxygenase, small subunit	HcaF	4.83	0.195	13.8	0.000257

[1] log-transformed fold change; [2] Computed with eBayes on a model fit generated by the fitZig function of the metagenomeSeq package.

Table S9. Details on the bins reconstructed with the coassembled tobacco rhizosphere metagenomes.

Bin ID	Bin taxonomy	Presence 16S[1]	Length	GC percentage	Number of contigs	Complete-ness (%)[2]	Contamina-tion (%)[2]
Bin1	k_Bacteria;p_Proteobacteria;c_Gammaproteobacteria;o_Pseudomonadales;f_Pseudomonadaceae;g_*Pseudomonas*;	Yes	7072145	60.47	456	99.68	2.04
Bin6	k_Bacteria;p_Bacteroidetes;	No	3874263	40.98	334	94.1	9.34
Bin7	k_Bacteria;p_Acidobacteria;	No	6930324	70.19	709	93.65	8.78
Bin13	k_Bacteria;p_Proteobacteria;c_Alphaproteobacteria;o_Sphingomonadales;f_Sphingomonadaceae;g_*Sphingobium*;	Yes	4640993	61.73	346	91.38	9.49
Bin17	k_Bacteria;p_Proteobacteria;c_Alphaproteobacteria;o_Sphingomonadales;	Yes	2320301	62.26	202	88.03	0.81
Bin18	k_Bacteria;p_Actinobacteria;c_Actinobacteria;o_Actinomycetales;f_Micrococcaceae;g_*Arthrobacter*;	Yes	4217553	62.16	248	87.85	2.47
Bin21	k_Bacteria;p_Gemmatimonadota;	No	3280048	65.93	430	85.11	4.03
Bin33	k_Bacteria;p_Bacteroidetes;c_Bacteroidia;o_Bacteroidales;	No	3659447	43.61	237	81.46	3.57
Bin34	k_Bacteria;p_Proteobacteria;c_Betaproteobacteria;o_Rhodocyclales;f_Rhodocyclaceae;	No	3241617	63.85	166	81.33	4.17
Bin41	k_Bacteria;p_Actinobacteria;c_Actinobacteria;o_Actinomycetales;f_Micrococcaceae;g_*Arthrobacter*;	No	3951228	62.16	1175	79.51	4.2
Bin43	k_Bacteria;p_Acidobacteria;	Yes	4140814	57.1	226	79.29	3.62
Bin53	k_Bacteria;p_Actinobacteria;c_Actinobacteria;	No	2953295	71.41	446	75.71	4.15
Bin54	k_Bacteria;p_Actinobacteria;c_Actinobacteria;o_Actinomycetales;f_Micrococcaceae;g_*Arthrobacter*;	No	4477580	65.74	414	75.65	7.75
Bin57	k_Bacteria;p_Verrucomicrobia;c_Verrucomicrobiae;o_Verrucomicrobiales;	No	4244055	56.44	812	74.4	5.85
Bin59	k_Bacteria;p_Actinobacteria;c_Actinobacteria;o_Actinomycetales;	Yes	2083410	67.86	324	73.25	2.65
Bin65	k_Bacteria;p_Acidobacteriota;	No	3481165	69.22	412	72.26	2.07
Bin67	k_Bacteria;p_Acidobacteria;	No	4626723	54.86	885	71.81	4.71
Bin69	k_Bacteria;p_Gemmatimonadota;	No	1860047	61.72	427	71.39	0.94
Bin70	k_Bacteria;p_Proteobacteria;c_Betaproteobacteria;o_Rhodocyclales;f_Rhodocyclaceae;	No	3741381	66.37	227	71.34	2.59
Bin73	k_Bacteria;p_Proteobacteria;c_Alphaproteobacteria;o_Rhizobiales;	No	2032324	64.32	420	70.94	8.02
Bin74	k_Bacteria;p_Proteobacteria;c_Gammaproteobacteria;o_Xanthomonadales;f_Xanthomonadaceae;	Yes	2982469	64.31	870	70.88	8.75
Bin77	k_Bacteria;p_Actinobacteria;c_Actinobacteria;o_Actinomycetales;	No	2611336	72.68	403	70.16	2.63
Bin79	k_Bacteria;p_Nitrospirae;c_Nitrospira;o_Nitrospirales;f_Nitrospiraceae;	No	2626993	56.63	357	69.8	5.17
Bin82	k_Bacteria;p_*Candidatus* Rokubacteria;	Yes	2403090	67.86	564	68.34	2.41
Bin84	k_Bacteria;p_Bacteroidetes;c_Bacteroidia;o_Bacteroidales;	No	3231725	44.77	585	67.94	2.27
Bin88	k_Bacteria;p_Proteobacteria;c_Deltaproteobacteria;o_Myxococcales;f_Myxococcaceae;g_*Anaeromyxobacter*;	No	3371771	73.33	515	66.41	8.09
Bin93	k_Bacteria;p_Actinobacteria;c_Actinobacteria;	No	2140222	64.35	474	65.1	7.68
Bin97	k_Bacteria;p_Acidobacteria;	No	3987546	65.53	285	64.65	6.53

Bin101	k_Bacteria;p_Chloroflexi;	No	3173776	56.02	650	64.09	6.51
Bin102	k_Bacteria;p_Acidobacteria;	No	2683318	53.48	591	63.93	9.33
Bin103	k_Bacteria;p_Actinobacteria;c_Actinobacteria;	No	2094214	69.06	397	63.42	7.7
Bin110	k_Bacteria;p_Actinobacteria;c_Actinobacteria;o_Actinomycetales;f_Streptomycetaceae;g_*Streptomyces*;	No	5884374	70.02	2965	62.07	5.82
Bin118	k_Bacteria;p_Planctomycetes;c_Planctomycetia;o_Planctomycetales;f_Planctomycetaceae;	Yes	3040886	64.4	604	60.44	4.06
Bin120	k_Bacteria;p_Actinobacteria;c_Actinobacteria;	Yes	2194796	67.77	507	60.31	8.63
Bin121	k_Bacteria;p_Bacteroidetes;c_Sphingobacteriia;o_Sphingobacteriales;f_Chitinophagaceae;	Yes	2837892	42.86	629	60.31	3.51
Bin122	k_Bacteria;p_Gemmatimonadota;	No	3485597	66.35	658	60.16	4.31
Bin123	k_Bacteria;p_Verrucomicrobia;c_Verrucomicrobiae;o_Verrucomicrobiales;f_Verrucomicrobiaceae;	Yes	2117901	58.85	270	59.63	3.95
Bin124	k_Bacteria;p_Verrucomicrobia;c_Verrucomicrobiae;o_Verrucomicrobiales;f_Verrucomicrobiaceae;	Yes	2524814	56.89	605	59.63	9.19
Bin129	k_Bacteria;p_Actinobacteria;c_Actinobacteria;	No	1760400	69.04	196	58.19	7.14
Bin132	k_Bacteria;p_Proteobacteria;c_Betaproteobacteria;o_Burkholderiales;f_Oxalobacteraceae;g_*Herbaspirillum*;	No	3011020	63.18	1495	57.53	3.16
Bin133	k_Bacteria;p_Actinobacteria;c_Actinobacteria;	Yes	2386322	70.46	429	57	9.55
Bin135	k_Bacteria;p_Planctomycetota;	No	2864795	58.69	700	56.82	8.23
Bin137	k_Bacteria;p_Bacteroidetes;c_Sphingobacteriia;o_Sphingobacteriales;f_Chitinophagaceae;	No	3392798	40.62	559	55.68	5.45
Bin138	k_Bacteria;p_Bacteroidetes;c_Sphingobacteriia;o_Sphingobacteriales;f_Chitinophagaceae;	No	2602680	39.91	652	55.23	9.53
Bin139	k_Bacteria;p_Proteobacteria;c_Alphaproteobacteria;	No	4068294	64.75	864	54.39	5.74
Bin141	k_Bacteria;p_Acidobacteria;	No	3156916	55.48	718	54.19	2.91
Bin144	k_Bacteria;p_Proteobacteria;c_Alphaproteobacteria;o_Rhizobiales;	No	2163113	64.07	327	52.72	2.24
Bin148	k_Bacteria;p_Proteobacteria;c_Betaproteobacteria;o_Burkholderiales;	No	1775151	60.66	549	51.72	2.52
Bin149	k_Bacteria;p_Verrucomicrobia;c_Verrucomicrobiae;o_Verrucomicrobiales;f_Verrucomicrobiaceae;	No	1573592	58.02	330	51.71	4.76
Bin151	k_Bacteria;p_Chloroflexi;	No	3559390	51.89	743	51.36	5.37
Bin153	k_Bacteria;p_Planctomycetes;c_Planctomycetia;o_Planctomycetales;f_Planctomycetaceae;	No	3635929	66.13	2099	50.8	7.63
Bin155	k_Archaea;p_Thaumarchaeota;c_Nitrosopumilales;o_Nitrosopumilales;	No	1175159	29.34	266	50.21	4

[1]'Yes' if at least one sequence in the bin was assigned to 16S rRNA, 'No' otherwise.

[2]As computed by CheckM (Parks *et al.*, 2015). Parks DH, Imelfort M, Skennerton CT, Hugenholtz P, Tyson GW. CheckM : Assessing the quality of microbial genomes recovered from isolates, single cells, and metagenomes. Genome Res. 2015;25:1043-55.

Table S10. AntiSMASH annotation for bins that are >50% complete and <10% contaminated.

Bin ID	Type	Most similar known cluster	Similarity (%)
Bin1	NRPS	bicornutin A1/bicornutin A2	100
Bin1	NRPS-like	fragin	37
Bin1	T3PKS	2,4-diacetylphloroglucinol	100
Bin1	hserlactone	cepacin A	12
Bin1	NRPS	Pf-5 pyoverdine	8
Bin1	NRPS	taiwachelin	11
Bin1	NRPS, NRP-metallophore	cepaciachelin	25
Bin1	hydrogen-cyanide	hydrogen cyanide	100
Bin1	redox-cofactor	lankacidin C	13
Bin1	NRPS	azotobactin D	41
Bin1	arylpolyene	APE Vf	40
Bin1	NRPS	pyoverdine SMX-1	19
Bin6	arylpolyene	flexirubin	48
Bin13	terpene	zeaxanthin	100
Bin13	NI-siderophore	staphyloferrin B	16
Bin17	terpene	carotenoid	40
Bin18	betalactone	microansamycin	7
Bin18	NI-siderophore	desferrioxamine E	100
Bin18	NAPAA	stenothricin	31
Bin18	terpene	carotenoid	28
Bin21	NRPS	oxazolepoxidomycin A	20
Bin33	terpene	lipopolysaccharide	5
Bin34	terpene	vatiamide A/B/C/D/E/F	6
Bin41	NAPAA	epsilon-Poly-L-lysine	100
Bin41	terpene	carotenoid	28
Bin53	other	chejuenolide A/B	7
Bin54	NI-siderophore	desferrioxamine E	100
Bin54	terpene	carotenoid	50
Bin57	NRPS, T1PKS	puwainaphycin F, minutissamide A/B/C/D	15
Bin59	terpene	isorenieratene	71
Bin59	T3PKS	alkylresorcinol	100
Bin59	T3PKS	alkylresorcinol	100
Bin67	NRPS	heinamide A1/A2/A3/B1/B2/B3/B4/B5	36
Bin67	NRPS-like	aranazole A/B/C/D	12
Bin67	NRPS	nostopeptolide A2	37
Bin70	betalactone	plipastatin	15
Bin70	hserlactone	gamexpeptide A/B/E, luminmide B/D/E/G	10
Bin70	RRE-containing	polyhydroxyalkanoate	75
Bin74	NRPS-like	icosalide A/b	100
Bin74	NRPS	bovienimide A	100
Bin84	arylpolyene	flexirubin	16
Bin84	arylpolyene	flexirubin	8
Bin84	resorcinol	flexirubin	8
Bin84	arylpolyene	flexirubin	12
Bin88	NRPS-like	VEPE/AEPE/TG-1	80

Bin88	T3PKS	alkylpyrone-407/alkylpyrone-393	6
Bin97	NRPS	kolossin	100
Bin110	RiPP-like	bottromycin D	18
Bin110	transAT-PKS-like	kirromycin	11
Bin110	NRPS	cycloserine	9
Bin110	NRPS-like	kirromycin	7
Bin110	blactam	alanylclavam/2-hydroxymethylclavam/2-formyloxymethylclavam/clavam-2-carboxylate/clavulanic acid	33
Bin110	indole	5-isoprenylindole-3-carboxylate beta-D-glycosyl ester	14
Bin110	NRPS	rhizomide A/B/C	100
Bin110	other	tambjamine BE-18591	7
Bin110	NAPAA	epsilon-Poly-L-lysine	100
Bin124	NRPS	rhizomide A/B/C	100
Bin133	terpene	isorenieratene	28
Bin141	T1PKS	1-heptadecene	100

Table S11. Subject strains used for the comparison with the *Pseudomonas* bin 1 with dDDH values and G+C difference when compared to Bin 1.

Subject strain	Assembly accession	dDDH (d_0, in %) [C.I.]	dDDH (d_4, %) [C.I.]	dDDH (d_6, %) [C.I.]	G+C content difference (%)
Pseudomonas kilonensis DSM 13647	GCA_001269885	84.8 [81.1 - 87.9]	89 [86.5 - 91.0]	88.4 [85.5 - 90.8]	0.39
Pseudomonas zarinae SWRI108	GCA_014268695	69.7 [65.7 - 73.3]	66.4 [63.5 - 69.3]	71.3 [67.8 - 74.5]	0.39
Pseudomonas ogarae F113T	GCF_000237065	64.7 [60.9 - 68.3]	65 [62.0 - 67.8]	66.5 [63.1 - 69.8]	0.31
Pseudomonas brassicacearum LMG 21623	GCF_900103245	65.1 [61.3 - 68.7]	59.1 [56.3 - 61.9]	65.7 [62.3 - 68.9]	0.33
Pseudomonas beijingensis FP830	GCF_030687295	64.5 [60.7 - 68.1]	47.9 [45.3 - 50.5]	62.1 [58.8 - 65.3]	0.54
Pseudomonas thivervalensis LMG 21626	GCA_001637285	63 [59.2 - 66.6]	47.4 [44.8 - 50.0]	60.7 [57.4 - 63.8]	0.72
Pseudomonas bijieensis L22-9	GCA_013347965	62 [58.3 - 65.6]	46.3 [43.7 - 48.9]	59.5 [56.3 - 62.7]	0.38
Pseudomonas zanjanensis SWRI12	GCA_014268745	59.4 [55.7 - 62.9]	46.1 [43.5 - 48.7]	57.3 [54.1 - 60.4]	0.75
Pseudomonas viciae 11K1	GCA_004786035	57.3 [53.7 - 60.8]	39.8 [37.3 - 42.4]	53.5 [50.4 - 56.6]	0.14
Pseudomonas tehranensis SWRI196	GCA_014268615	54 [50.4 - 57.4]	35.3 [32.9 - 37.8]	49.3 [46.2 - 52.3]	0
Pseudomonas chlororaphis subsp. *phenazini* S1Bt23	GCA_034044175	30.3 [26.9 - 33.9]	27.3 [24.9 - 29.8]	28.5 [25.6 - 31.6]	2.4
Pseudomonas chlororaphis LMG 5004	GCA_001269625	30.4 [27.0 - 34.0]	27.2 [24.9 - 29.7]	28.6 [25.7 - 31.7]	2.54
Pseudomonas chlororaphis subsp. *aureofaciens* NBRC 3521	GCA_000813225	30 [26.6 - 33.6]	27.2 [24.9 - 29.7]	28.2 [25.3 - 31.3]	2.28
Pseudomonas protegens CHA0	GCA_000397205	27.6 [24.3 - 31.3]	26.3 [23.9 - 28.7]	26.2 [23.3 - 29.3]	2.92
Pseudomonas saponiphila DSM 9751	GCA_900105185	25.9 [22.6 - 29.5]	26.3 [24.0 - 28.8]	24.8 [21.9 - 27.9]	2.32
Pseudomonas danubii JDS02PS016	GCA_042980055	26.9 [23.5 - 30.5]	26.1 [23.7 - 28.5]	25.6 [22.7 - 28.7]	1.83
Pseudomonas idahonensis ID357	GCA_028657095	26.6 [23.2 - 30.2]	26 [23.7 - 28.5]	25.3 [22.4 - 28.4]	1.65
Pseudomonas aestus CMAA1215	GCA_000474765	25.4 [22.0 - 29.0]	25.8 [23.5 - 28.3]	24.3 [21.4 - 27.4]	3.33
Pseudomonas piscis KCTC 72033	GCA_009380155	26 [22.7 - 29.6]	25.7 [23.4 - 28.2]	24.8 [21.9 - 27.9]	3.1
Pseudomonas veronii subsp. *inensis* JCM 11828	GCA_039522465	26.1 [22.8 - 29.8]	25.6 [23.3 - 28.1]	24.9 [22.0 - 28.0]	0.18
Pseudomonas sessilinigenes CMR12a	GCA_003850565	25 [21.7 - 28.7]	25.5 [23.2 - 28.0]	23.9 [21.1 - 27.0]	2.33
Pseudomonas syringae KCTC 12500	GCA_000507185	18.6 [15.5 - 22.2]	23 [20.7 - 25.5]	18.4 [15.7 - 21.4]	1.55
Pseudomonas fragariae 17	GCA_032681325	18.9 [15.7 - 22.4]	23 [20.7 - 25.4]	18.6 [15.9 - 21.6]	1.25
Pseudomonas maioricensis S25	GCF_022790535	17.5 [14.5 - 21.1]	22.9 [20.6 - 25.3]	17.4 [14.8 - 20.4]	3.29
Pseudomonas meliae CFBP 3225	GCF_000935675	18.5 [15.4 - 22.1]	22.8 [20.5 - 25.2]	18.2 [15.6 - 21.2]	2.07
Pseudomonas amygdali CFBP 3205	GCA_000935645	18.3 [15.2 - 21.9]	22.7 [20.4 - 25.1]	18.1 [15.4 - 21.1]	2.19

Table S12. PERMANOVA results for the metabolome of tobacco shoots.

Source	df	F	*P*	Signif. code	η^2 (%)
Geography	1	7.20	<0.001	***	5.11
Geology	1	4.76	<0.001	***	3.38
Inoculation	1	6.12	<0.001	***	4.34
Geography:Geology	1	5.06	<0.001	***	3.59
Geography:Inoculation	1	6.07	<0.001	***	4.31
Geology:Inoculation	1	2.55	0.014	*	1.81
Geography:Geology:Soil	4	4.42	<0.001	***	12.6
Geography:Geology:Inoculation	1	1.12	0.32	NS	0.797
Geography:Geology:Soil:Inoculation	4	1.83	0.006	**	5.19
Residual	83				
Total	98				

Table S13. Main physicochemical characteristics of Swiss and Savoie soils (all cambisols).

Soil	MS7	MS16	MC10	MC112	Amo1	Ymo4	Asa2	Ysa5
Clay (%)	18.9	10.9	13.5	17.9	15.6	11.5	12.8	13.0
Silt (%)	20.4	25.4	25.5	24.8	25.2	20.1	14.1	21.9
Sand (%)	60.7	63.7	61	57.3	59.2	68.4	73.1	65.1
CEC[1] (cmol/kg)	12.03	11.20	48.38	15.83	22.32	48.79	26.81	48.09
SAB[2] (%)	56.51	81.43	98.93	65.09	75.10	98.67	96.24	99.19
pH in H_2O	6.05	6.82	7.91	5.84	5.96	7.74	7.30	8.01
Organic matter (%)	2.48	2.12	3.92	2.98	5.19	3.68	1.65	2.77
Total N (%)	0.12	0.11	0.20	0.15	0.26	0.18	0.08	0.14
P (mg/100 g dry soil)[3]	5.66	6.12	15.00	2.28	2.91	9.01	14.17	27.92
K (mg/100 g dry soil)[4]	18.84	14.69	20.84	10.79	7.55	12.12	15.11	27.89
Total Fe (%)[5]	1.407	1.282	1.518	1.808	2.06	1.69	1.85	1.95

Analysis was done by the Fruit Research Institute in Čačak (Serbia). All soils displayed a sandy-loam texture.

[1]CEC: Cation Exchange Capacity, determined using the Kappen method.

[2]SAB: Saturation with Adsorbed Bases (sum of exchangeable cations adsorbed with CEC)

[3]Readily-available phosphorus was extracted with ammonium lactate.

[4]Readily-available potassium was extracted with ammonium lactate.

[5]Total Fe was extracted with HCl:HNO_3 in a 1:3 ratio.

Table S14. Quality control for the shotgun metagenome assembly (contigs statistics) and binning (bin statistics).

**Contigs statistics**	**Value**
Number of contigs	33,662,575
Total length (bp)	20,487,011,590
Longest contig (bp)	195,748
Shortest contig (bp)	200
N50	627
N90	344
Contigs taxonomy distribution	
Contigs at superkingdom (k) rank	29,555,506 (87.8%), in 4 superkingdoms
Contigs at phylum (p) rank	23505357 (69.8%), in 232 phyla
Contigs at class (c) rank	12097830 (35.9%), in 274 classes
Contigs at order (o) rank	7431965 (22.1%), in 599 orders
Contigs at family (f) rank	5074739 (15.1%), in 1211 families
Contigs at genus (g) rank	2717826 (8.1%), in 3569 genera
Contigs at species (s) rank	2150266 (6.4%), in 5971 species
Number of congruent contigs	33,647,264 (100.0%)
Number of contigs with disparity > 0	15,312 (0.0%)
Number of contigs with disparity ≥ 0.25	13,630 (0.0%)
**Bin statistics**	**Value**
Number of bins	301
Completeness distribution	
Complete ≥ 50%	155
Complete ≥ 75%	56
Complete ≥ 90%	15
Contamination distribution	
Contamination < 5%	1
Contamination < 10%	183
Contamination ≥ 50%	27
Number of good quality bins (> 90% complete, < 5% contaminated)	1
Number of medium-quality bins (> 50% complete, < 10% contaminated)	52
